# Fine-mapping identifies two additional breast cancer susceptibility loci at 9q31.2

**DOI:** 10.1093/hmg/ddv035

**Published:** 2015-02-04

**Authors:** Nick Orr, Frank Dudbridge, Nicola Dryden, Sarah Maguire, Daniela Novo, Eleni Perrakis, Nichola Johnson, Maya Ghoussaini, John L. Hopper, Melissa C. Southey, Carmel Apicella, Jennifer Stone, Marjanka K. Schmidt, Annegien Broeks, Laura J. Van't Veer, Frans B. Hogervorst, Peter A. Fasching, Lothar Haeberle, Arif B. Ekici, Matthias W. Beckmann, Lorna Gibson, Zoe Aitken, Helen Warren, Elinor Sawyer, Ian Tomlinson, Michael J. Kerin, Nicola Miller, Barbara Burwinkel, Frederik Marme, Andreas Schneeweiss, Chistof Sohn, Pascal Guénel, Thérèse Truong, Emilie Cordina-Duverger, Marie Sanchez, Stig E. Bojesen, Børge G. Nordestgaard, Sune F. Nielsen, Henrik Flyger, Javier Benitez, Maria Pilar Zamora, Jose Ignacio Arias Perez, Primitiva Menéndez, Hoda Anton-Culver, Susan L. Neuhausen, Hermann Brenner, Aida Karina Dieffenbach, Volker Arndt, Christa Stegmaier, Ute Hamann, Hiltrud Brauch, Christina Justenhoven, Thomas Brüning, Yon-Dschun Ko, Heli Nevanlinna, Kristiina Aittomäki, Carl Blomqvist, Sofia Khan, Natalia Bogdanova, Thilo Dörk, Annika Lindblom, Sara Margolin, Arto Mannermaa, Vesa Kataja, Veli-Matti Kosma, Jaana M. Hartikainen, Georgia Chenevix-Trench, Jonathan Beesley, Diether Lambrechts, Matthieu Moisse, Guiseppe Floris, Benoit Beuselinck, Jenny Chang-Claude, Anja Rudolph, Petra Seibold, Dieter Flesch-Janys, Paolo Radice, Paolo Peterlongo, Bernard Peissel, Valeria Pensotti, Fergus J. Couch, Janet E. Olson, Seth Slettedahl, Celine Vachon, Graham G. Giles, Roger L. Milne, Catriona McLean, Christopher A. Haiman, Brian E. Henderson, Fredrick Schumacher, Loic Le Marchand, Jacques Simard, Mark S. Goldberg, France Labrèche, Martine Dumont, Vessela Kristensen, Grethe Grenaker Alnæs, Silje Nord, Anne-Lise Borresen-Dale, Wei Zheng, Sandra Deming-Halverson, Martha Shrubsole, Jirong Long, Robert Winqvist, Katri Pylkäs, Arja Jukkola-Vuorinen, Mervi Grip, Irene L. Andrulis, Julia A. Knight, Gord Glendon, Sandrine Tchatchou, Peter Devilee, Robertus A. E. M. Tollenaar, Caroline M. Seynaeve, Christi J. Van Asperen, Montserrat Garcia-Closas, Jonine Figueroa, Stephen J. Chanock, Jolanta Lissowska, Kamila Czene, Hatef Darabi, Mikael Eriksson, Daniel Klevebring, Maartje J. Hooning, Antoinette Hollestelle, Carolien H. M. van Deurzen, Mieke Kriege, Per Hall, Jingmei Li, Jianjun Liu, Keith Humphreys, Angela Cox, Simon S. Cross, Malcolm W. R. Reed, Paul D. P. Pharoah, Alison M. Dunning, Mitul Shah, Barbara J. Perkins, Anna Jakubowska, Jan Lubinski, Katarzyna Jaworska-Bieniek, Katarzyna Durda, Alan Ashworth, Anthony Swerdlow, Michael Jones, Minouk J. Schoemaker, Alfons Meindl, Rita K. Schmutzler, Curtis Olswold, Susan Slager, Amanda E. Toland, Drakoulis Yannoukakos, Kenneth Muir, Artitaya Lophatananon, Sarah Stewart-Brown, Pornthep Siriwanarangsan, Keitaro Matsuo, Hidema Ito, Hiroji Iwata, Junko Ishiguro, Anna H. Wu, Chiu-chen Tseng, David Van Den Berg, Daniel O. Stram, Soo Hwang Teo, Cheng Har Yip, Peter Kang, Mohammad Kamran Ikram, Xiao-Ou Shu, Wei Lu, Yu-Tang Gao, Hui Cai, Daehee Kang, Ji-Yeob Choi, Sue K. Park, Dong-Young Noh, Mikael Hartman, Hui Miao, Wei Yen Lim, Soo Chin Lee, Suleeporn Sangrajrang, Valerie Gaborieau, Paul Brennan, James Mckay, Pei-Ei Wu, Ming-Feng Hou, Jyh-Cherng Yu, Chen-Yang Shen, William Blot, Qiuyin Cai, Lisa B. Signorello, Craig Luccarini, Caroline Bayes, Shahana Ahmed, Mel Maranian, Catherine S. Healey, Anna González-Neira, Guillermo Pita, M. Rosario Alonso, Nuria Álvarez, Daniel Herrero, Daniel C. Tessier, Daniel Vincent, Francois Bacot, David J. Hunter, Sara Lindstrom, Joe Dennis, Kyriaki Michailidou, Manjeet K. Bolla, Douglas F. Easton, Isabel dos Santos Silva, Olivia Fletcher, Julian Peto

**Affiliations:** 1 The Breakthrough Breast CancerResearch Centre,; 2 Division of Breast Cancer Research and; 3 Division of Genetics and Epidemiology, The Institute of Cancer Research, London, UK,; 4 Department of Non-Communicable DiseaseEpidemiology, London School of Hygiene and Tropical Medicine, London, UK,; 5 Centre for Cancer Genetic Epidemiology, Department of Oncology and; 6 Centre for Cancer Genetic Epidemiology, Department of Public Health and Primary Care, University of Cambridge, Cambridge, UK,; 7 Centre for Epidemiology and Biostatistics, Melbourne School of Population and Global Health and; 8 Department of Pathology, The University of Melbourne, Melbourne, VIC 3010, Australia,; 9 Centre for Genetic Origins of Health and Disease, University of Western Australia, Perth, WA, Australia,; 10 Netherlands Cancer Institute, Antoni van Leeuwenhoek Hospital, 1066 CX Amsterdam, The Netherlands,; 11 University Breast Center Franconia, Department of Gynecology and Obstetrics, University Hospital Erlangen and; 12 Institute of Human Genetics, University Hospital Erlangen, Friedrich-Alexander University Erlangen-Nuremberg, Comprehensive Cancer Center Erlangen-EMN, 91054 Erlangen, Germany,; 13 David Geffen School of Medicine, Department of Medicine Division of Hematology and Oncology, University of California at Los Angeles, Los Angeles, CA 90095, USA,; 14 Department of Clinical Pharmacology, William Harvey Research Institute, Barts and The London School of Medicine and; 15 NIHR Barts Cardiovascular Biomedical Research Unit, Queen Mary University of London, London, UK,; 16 Division of Cancer Studies, Kings College London, Guy's Hospital, London, UK,; 17 Wellcome Trust Centre for Human Genetics and Oxford Biomedical Research Centre, University of Oxford, UK,; 18 Clinical Science Institute, University Hospital Galway, Galway, Ireland,; 19 Department of Obstetrics and Gynecology and; 20 National Center for Tumor Diseases, University of Heidelberg, 69120 Heidelberg, Germany,; 21 Molecular Epidemiology Group, German Cancer Research Center (DKFZ), 69120 Heidelberg, Germany,; 22 Inserm (National Institute of Health and Medical Research), CESP (Center for Research in Epidemiology and Population Health), U1018, Environmental Epidemiology of Cancer, Villejuif, France,; 23 University Paris-Sud, UMRS 1018, Villejuif, France,; 24 Copenhagen General Population Study and; 25 Department of Clinical Biochemistry, Herlev Hospital, Copenhagen University Hospital, 2730 Herlev, Denmark,; 26 Department of Breast Surgery, Herlev Hospital, Copenhagen University Hospital, Copenhagen, Denmark,; 27 Human Genetics Group, Human Cancer Genetics Program, Spanish National Cancer Research Centre (CNIO), 28029 Madrid, Spain,; 28 Centro de Investigación en Red de Enfermedades Raras (CIBERER), 46010 Valencia, Spain,; 29 Servicio de Oncología Médica, Hospital Universitario La Paz, 28046 Madrid, Spain,; 30 Servicio de Cirugía General y Especialidades and; 31 Servicio de Anatomía Patológica, Hospital Monte Naranco, 33012 Oviedo, Spain,; 32 Department of Epidemiology, University of California Irvine, Irvine, CA, USA,; 33 Beckman Research Institute of City of Hope, Duarte, CA, USA,; 34 Division of Clinical Epidemiology and Aging Research,; 35 German Cancer Consortium (DKTK),; 36 Molecular Genetics of Breast Cancer and; 37 Division of Cancer Epidemiology, German Cancer Research Center (DKFZ), Heidelberg, Germany,; 38 Saarland Cancer Registry, Saarbrücken, Germany,; 39 Dr Margarete Fischer-Bosch-Institute of Clinical Pharmacology, Stuttgart, Germany,; 40 University of Tübingen, Tübingen, Germany,; 41 Institute for Prevention and Occupational Medicine of the German Social Accident Insurance, Institute of the Ruhr—University Bochum (IPA), Bochum, Germany,; 42 Department of Internal Medicine, Evangelische Kliniken Bonn gGmbH, Johanniter Krankenhaus, Bonn, Germany,; 43 Institute of Pathology, Medical Faculty of the University of Bonn, Bonn, Germany,; 44 Institute of Occupational Medicine and Maritime Medicine, University Medical Center Hamburg-Eppendorf, Hamburg, Germany,; 45 Department of Obstetrics and Gynecology,; 46 Department of Clinical Genetics and; 47 Department of Oncology, University of Helsinki and Helsinki University Hospital, Helsinki, Finland,; 48 Department of Radiation Oncology and; 49 Department of Obstetrics and Gynaecology, Hannover Medical School, Hannover, Germany,; 50 Department of Molecular Medicine and Surgery and; 51 Department of Oncology-Pathology, Karolinska Institutet, Stockholm, Sweden,; 52 School of Medicine, Institute of Clinical Medicine, Pathology and Forensic Medicine and; 53 School of Medicine, Institute of Clinical Medicine, Oncology, University of Eastern Finland, Kuopio, Finland,; 54 Imaging Center, Department of Clinical Pathology and; 55 Biocenter Kuopio, Cancer Center of Eastern Finland, Kuopio University Hospital, Kuopio, Finland,; 56 Central Finland Health Care District, Jyväskylä Central Hospital, Jyväskylä, Finland,; 57 QIMR Berghofer Medical Research Institute, Brisbane, QLD, Australia,; 58 Peter MacCallum Cancer Center, Melbourne, VIC, Australia,; 59 Vesalius Research Center (VRC), VIB, Leuven, Belgium,; 60 Laboratory for Translational Genetics, Department of Oncology, University of Leuven, Leuven, Belgium,; 61 University Hospital Gasthuisberg, Leuven, Belgium,; 62 Department of Cancer Epidemiology/Clinical Cancer Registry and; 63 Institute for Medical Biometrics and Epidemiology, University Clinic Hamburg-Eppendorf, Hamburg, Germany,; 64 Unit of Molecular Bases of Genetic Risk and Genetic Testing, Department of Preventive and Predictive Medicine and; 65 Unit of Medical Genetics, Department of Preventive and Predictive Medicine, Fondazione IRCCS Istituto Nazionale dei Tumori (INT), Milan, Italy,; 66 IFOM, Fondazione Istituto FIRC di Oncologia Molecolare, Milan, Italy,; 67 Cogentech Cancer Genetic Test Laboratory, Milan, Italy,; 68 Department of Laboratory Medicine and Pathology and; 69 Department of Health Sciences Research, Mayo Clinic, Rochester, MN, USA,; 70 Cancer Epidemiology Centre, Cancer Council Victoria, Melbourne, VIC, Australia,; 71 Anatomical Pathology, The Alfred Hospital, Melbourne, VIC, Australia,; 72 Department of Preventive Medicine, Keck School of Medicine, University of Southern California, Los Angeles, CA, USA,; 73 Epidemiology Program, Cancer Research Center, University of Hawaii, Honolulu, HI, USA,; 74 Cancer Genomics Laboratory, Centre Hospitalier Universitaire de Québec Research Center and Laval University, Quebec City, QC, Canada,; 75 Department of Medicine, McGill University, Montreal, QC, Canada,; 76 Division of Clinical Epidemiology, McGill University Health Centre, Royal Victoria Hospital, Montreal, QC, Canada,; 77 Département de Médecine Sociale et Préventive, Département de Santé Environnementale et Santé au travail, Université de Montréal, Montreal, QC, Canada,; 78 Department of Genetics, Institute for Cancer Research, Oslo University Hospital, Radiumhospitalet, N-0310 Oslo, Norway,; 79 Institute of Clinical Medicine, University of Oslo (UiO), 0450 Oslo, Norway,; 80 Division of Epidemiology, Department of Medicine, Vanderbilt Epidemiology Center, Vanderbilt-Ingram Cancer Center, Vanderbilt University School of Medicine, Nashville, TN, USA,; 81 Laboratory of Cancer Genetics and Tumor Biology, Department of Clinical Genetics and Biocenter Oulu,; 82 Department of Oncology and; 83 Department of Surgery, Oulu University Hospital, University of Oulu, Oulu, Finland,; 84 Ontario Cancer Genetics Network and; 85 Prosserman Centre for Health Research, Lunenfeld-Tanenbaum Research Institute of Mount Sinai Hospital, Toronto, ON, Canada,; 86 Department of Molecular Genetics and; 87 Division of Epidemiology, Dalla Lana School of Public Health, University of Toronto, Toronto, ON, Canada,; 88 Lunenfeld-Tanenbaum Research Institute of Mount Sinai Hospital, Toronto, ON, Canada,; 89 Department of Human Genetics and Department of Pathology and; 90 Department of Surgical Oncology, Leiden University Medical Center, 2300 RC, Leiden, The Netherlands,; 91 Department of Medical Oncology, Family Cancer Clinic, Erasmus MC Cancer Institute, Rotterdam, The Netherlands,; 92 Department of Clinical Genetics, Leiden University Medical Center, P.O. Box 9600, 2300 RC, Leiden, The Netherlands,; 93 Division of Cancer Epidemiology and Genetics, National Cancer Institute, Rockville, MD, USA,; 94 Department of Cancer Epidemiology and Prevention, M. Sklodowska-Curie Memorial Cancer Center & Institute of Oncology, Warsaw, Poland,; 95 Department of Medical Epidemiology and Biostatistics, Karolinska Institutet, Stockholm 17177, Sweden,; 96 Department of Pathology, Erasmus University Medical Center, Rotterdam, The Netherlands,; 97 Human Genetics Division, Genome Institute of Singapore, Singapore 138672, Singapore,; 98 Sheffield Cancer Research, Department of Oncology, University of Sheffield, Sheffield, UK,; 99 Academic Unit of Pathology, Department of Neuroscience, University of Sheffield, Sheffield, UK,; 100 Brighton and Sussex Medical School, University of Sussex, Brighton, East Sussex, UK,; 101 Department of Genetics and Pathology, Pomeranian Medical University, 70-115 Szczecin, Poland,; 102 Division of Gynaecology and Obstetrics, Technische Universität München, 81675 Munich, Germany,; 103 Center for Hereditary Breast and Ovarian Cancer, Center for Integrated Oncology (CIO) and Center for Molecular Medicine Cologne (CMMC), Medical Faculty, University of Cologne and University Hospital Cologne, Cologne, Germany,; 104 Department of Molecular Virology, Immunology and Medical Genetics, Comprehensive Cancer Center, The Ohio State University, Columbus, OH, USA,; 105 Molecular Diagnostics Laboratory, IRRP, National Centre for Scientific Research ‘Demokritos’, Aghia Paraskevi Attikis, Athens, Greece,; 106 Division of Health Sciences, Warwick Medical School, Warwick University, Coventry, UK,; 107 Institute of Population Health, University of Manchester, Manchester, UK,; 108 Ministry of Public Health, Nonthaburi, Thailand,; 109 Department of Preventive Medicine, Kyushu University Faculty of Medical Sciences, Fukuoka, Japan,; 110 Division of Epidemiology and Prevention, Aichi Cancer Center Research Institute, Nagoya, Japan,; 111 Department of Breast Oncology, Aichi Cancer Center Hospital, Nagoya, Japan,; 112 Cancer Research Initiatives Foundation, Sime Darby Medical Centre, Subang Jaya, Selangor, Malaysia,; 113 Breast Cancer Research Unit, University Malaya Cancer Research Institute, University Malaya Medical Centre, Kuala Lumpur, Malaysia,; 114 Singapore Eye Research Institute, National University of Singapore, 168751 Singapore, Singapore,; 115 Shanghai Center for Disease Control and Prevention, Shanghai, China,; 116 Department of Epidemiology, Shanghai Cancer Institute, Shanghai, China,; 117 Department of Preventive Medicine and; 118 Department of Surgery, Seoul National University College of Medicine, Seoul, Korea,; 119 Department of Biomedical Sciences, Seoul National University Graduate School, Seoul, Korea,; 120 Cancer Research Institute, Seoul National University, Seoul, Korea,; 121 Saw Swee Hock School of Public Health and; 122 Department of Surgery, Yong Loo Lin School of Medicine, National University of Singapore, Singapore, Singapore,; 123 National University Health System, Singapore, Singapore,; 124 Department of Haematology-Oncology, National University Health System, Singapore, Singapore,; 125 Cancer Science Institute of Singapore, National University Singapore, Singapore, Singapore,; 126 National Cancer Institute, Bangkok, Thailand,; 127 International Agency for Research on Cancer, Lyon, France,; 128 Taiwan Biobank and; 129 Institute of Biomedical Sciences, Academia Sinica, Taipei 115, Taiwan,; 130 Cancer Center and Department of Surgery, Kaohsiung Medical University Chung-Ho Memorial Hospital, Kaohsiung 804, Taiwan,; 131 Department of Surgery, Tri-Service General Hospital and National Defense Medical Center, Taipei 114, Taiwan,; 132 School of Public Health, China Medical University, Taichung 404, Taiwan,; 133 International Epidemiology Institute, Rockville, MD, USA,; 134 Dana-Farber/Harvard Cancer Center, Boston, MA, USA,; 135 Department of Epidemiology and; 136 Program in Molecular and Genetic Epidemiology, Harvard School of Public Health, Boston, MA, USA,; 137 Human Genotyping-CEGEN Unit, Human Cancer Genetics Program, Spanish National Cancer Research Centre (CNIO), Madrid, Spain and; 138 Centre d'innovation Génome Québec et Université McGill, Montréal, QC, Canada

## Abstract

We recently identified a novel susceptibility variant, rs865686, for estrogen-receptor positive breast cancer at 9q31.2. Here, we report a fine-mapping analysis of the 9q31.2 susceptibility locus using 43 160 cases and 42 600 controls of European ancestry ascertained from 52 studies and a further 5795 cases and 6624 controls of Asian ancestry from nine studies. Single nucleotide polymorphism (SNP) rs676256 was most strongly associated with risk in Europeans (odds ratios [OR] = 0.90 [0.88–0.92]; *P*-value = 1.58 × 10^−25^). This SNP is one of a cluster of highly correlated variants, including rs865686, that spans ∼14.5 kb. We identified two additional independent association signals demarcated by SNPs rs10816625 (OR = 1.12 [1.08–1.17]; *P*-value = 7.89 × 10^−09^) and rs13294895 (OR = 1.09 [1.06–1.12]; *P*-value = 2.97 × 10^−11^). SNP rs10816625, but not rs13294895, was also associated with risk of breast cancer in Asian individuals (OR = 1.12 [1.06–1.18]; *P*-value = 2.77 × 10^−05^). Functional genomic annotation using data derived from breast cancer cell-line models indicates that these SNPs localise to putative enhancer elements that bind known drivers of hormone-dependent breast cancer, including ER-α, FOXA1 and GATA-3. *In vitro* analyses indicate that rs10816625 and rs13294895 have allele-specific effects on enhancer activity and suggest chromatin interactions with the *KLF4* gene locus. These results demonstrate the power of dense genotyping in large studies to identify independent susceptibility variants. Analysis of associations using subjects with different ancestry, combined with bioinformatic and genomic characterisation, can provide strong evidence for the likely causative alleles and their functional basis.

## Introduction

Breast cancer is the most common female cancer worldwide, in both developed and less developed regions, including Asia and Africa. An estimated 1.38 million new breast cancer cases were diagnosed worldwide in 2008, and this burden is likely to increase in the coming decades as a result of population ageing and adoption of western lifestyles (1).

Susceptibility to breast cancer involves contributions from genetic, environmental, lifestyle and hormonal factors. Pathogenic mutations in the DNA-repair genes *BRCA1* and *BRCA2* confer high lifetime risks of the disease and are responsible for the majority of cases that occur in families with many affected members but account for only 20% of the excess familial relative risk (FRR) of the disease (2). Rare germline variants in genes including *CHEK2*, *PALB2* and *ATM* each confer moderately increased relative risks (RR) of breast cancer but make only small contributions to the excess FRR (3–5). Genome-wide association studies (GWAS) have identified 79 single nucleotide polymorphisms (SNPs) that influence breast cancer susceptibility and explain a further 15% of the FRR (6–19). Statistical modelling suggests that several thousands of additional breast cancer susceptibility SNPs remain undetected (9). Genetic variants can be incorporated into risk prediction models that can stratify women by level of risk. The power of such models will improve as more variants are identified (20). One productive approach to identifying additional susceptibility variants is through fine-mapping of regions known to harbour susceptibility alleles.

The 9q31.2 breast cancer susceptibility locus, delineated by rs865686, was identified by a GWAS that utilised genetically enriched cases from the UK with either bilateral breast cancer or with a family history of the disease (7). A replication study using samples from the Breast Cancer Association Consortium (BCAC) indicated that the association with rs865686 was restricted to estrogen-receptor (ER) positive breast cancer (21). SNP rs865686 localises to a gene desert and consequently the mechanism of association is assumed to be through long-range regulation of target gene expression. The nearest neighbouring genes to rs865686 include Kruppel-like factor 4 (*KLF4*), RAD23 homologue B (*RAD23B*; both >600 kb proximal), actin-like 7B (*ACTL7B*) and inhibitor of kappa light polypeptide gene enhancer in B-cells, kinase complex-associated protein (*IKBKAP*; both >700 kb distal).

We performed a fine-mapping study, using over 85 000 European and 12 000 Asian ancestry samples from BCAC, in order to localise the causal variant underlying the association between rs865686 and susceptibility to breast cancer. In addition we assessed whether other independent breast cancer susceptibility SNPs could be detected at the 9q31.2 locus.

## Results

We successfully genotyped a total of 424 SNPs spanning 110 740 582–111 100 826 bp (NCBI HG37) on chromosome 9. These SNPs captured ∼94% and 86% of common 1000 Genomes Project (1KGP) variants at *r*^2^ ≥ 0.8 in European and Asian populations, respectively. Association analyses were performed using 85 760 subjects of European ancestry, 12 491 subjects of Asian ancestry and 1978 subjects of African ancestry (Supplementary Material, Table S1). We report only the results from the European and Asian studies, as there were too few samples for meaningful analyses of women of African ancestry. However, the full results from the European, Asian and African studies are presented in Supplementary Material, Table S2A–C. We used statistical imputation of unobserved genotypes to increase the density of our fine-mapping analysis; a total of 2035 SNPs and insertion/deletion (indel) polymorphisms were inferred using 1000 Genomes Project (1KGP) reference data, from which 1529 variants were imputed with high certainty (Impute2 (22) information measure ≥0.5) and included in subsequent association analyses. Because no imputed variant was more significantly associated with breast cancer risk than the highest ranked, directly genotyped SNPs, they were not considered in the following analyses unless explicitly stated.

**Figure 1.**
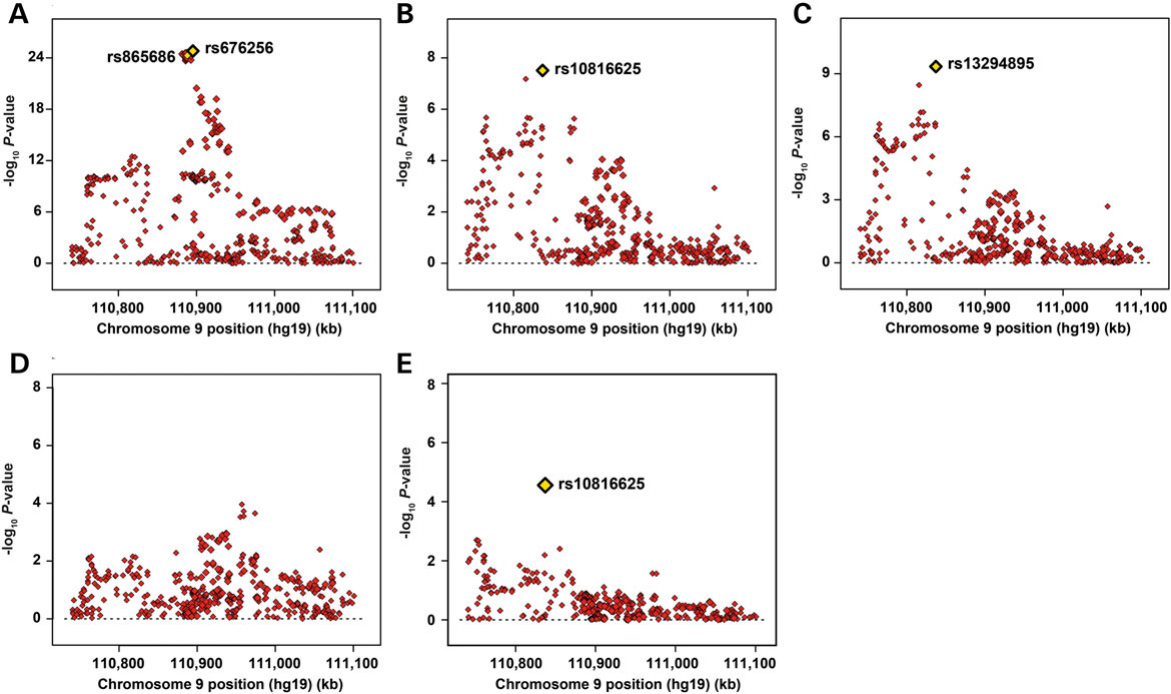
Regional association plots for 9q31.2 fine-mapping SNPs in European and Asian ancestry individuals. (**A**–**D**) Individual steps from a forward stepwise regression analysis using data from the Caucasian studies, in which the most strongly associated SNP from a given model is included as a covariate in the subsequent model. Chromosome position is indicated on the *x*-axis, and –log_10_ *P*-value on the *y*-axis. The models represented are adjusted for study and seven ancestry-informative principal components. Each directly genotyped SNP is represented as a single red diamond and the most significant SNP that attained genome-wide significance from each step of the stepwise regression is indicated by a yellow diamond. (**E**) Regional association plot for the 9q31.2 fine-mapping SNPs in subjects with Asian ancestry tested using a model adjusted for study and two ancestry-informative principal components.

The most significantly associated SNP was rs676256 (odds ratio [OR] = 0.90 [0.88–0.92]; *P* = 1.58 × 10^−25^; Fig. 1A and Table 1; Supplementary Material, Table S2A). SNP rs676256 was one of a 14.4 kb cluster of 38 genotyped or imputed correlated SNPs (*r*^2^ > 0.8 in controls of European ancestry) that also included SNP rs865686. Of the 38 SNPs correlated with rs676256 at *r*^2^ ≥ 0.8, 27 had likelihood ratios >1:100 relative to rs676256 (Supplementary Material, Table S3); hence it is likely that at least one of the 28 SNPs in this independent set of correlated highly associated variants (iCHAV) is causal (23).

**Table 1.** Association of rs10816625, rs13294895 and rs676256 with risk of breast cancer amongst women of European and Asian ancestry

Locus	Population	Control MAF	Control genotype counts			Case MAF	Case genotype counts			*P*-value^a^	OR^b^	95% CI^b^
rs10816625			AA	AG	GG		AA	AG	GG			
9q31.2	Caucasians	0.06	37579	4851	169	0.07	37 434	5560	164	7.89 × 10^−09^	1.12	1.08–1.17
110 837 073	Asians	0.38	2633	2976	1013	0.42	2023	2714	1057	2.77 × 10^−05^	1.12	1.06–1.18
rs13294895			GG	AG	AA		GG	AG	AA			
9q31.2	Caucasians	0.20	28 954	12 372	1272	0.19	28 625	13 029	1506	2.97 × 10^−11^	1.09	1.06–1.12
110 837 176	Asians	0.03	6244	372	8	0.03	5495	288	10	0.66	1.04	0.89–1.21
rs676256			AA	AG	GG		AA	AG	GG			
9q31.2	Caucasians	0.38	16166	20183	6250	0.36	18 011	19 670	5472	1.58 × 10^−25^	0.90	0.88–0.92
110 895 353	Asians	0.05	6036	567	21	0.04	5329	455	11	0.3	0.94	0.82–1.06

^a^*P*-values from single SNP test of association, computed from a likelihood-ratio test with one degree-of-freedom.

^b^Odds ratios and 95% confidence intervals for SNP association with breast cancer estimated using logistic regression, adjusting for study and significant principal components and assuming multiplicativity on the odds scale for heterozygote and minor-allele homozygote ORs.

To determine whether additional SNPs at 9q31.2 confer risks of breast cancer independently of rs676256, we fitted a series of stepwise logistic regression models (Fig. 1B–D), stopping when no additional SNPs reached genome-wide significance (Fig. 1D). We identified SNPs rs10816625 (stepwise OR = 1.12 [1.07–1.16]; *P* = 3.49 × 10^−08^; Fig. 1B) and rs13294895 (stepwise OR = 1.08 [1.06–1.11]; *P* = 4.56 × 10^−10^; Fig. 1C). The *P*-values and effect estimates for all three susceptibility SNPs, adjusted by study and ancestry-informative principal components, but not adjusted for the other SNPs, are shown in Table 1. All three SNPs remained strongly associated with breast cancer risk when modelled jointly (rs10816625: OR = 1.13 [1.09–1.18]; *P* = 5.04 × 10^−10^; rs13294895: OR = 1.08 [1.06–1.11]; *P* = 4.80 × 10^−10^; rs676256: OR = 0.91 [0.89–0.93]; *P* = 2.31 × 10^−21^). There was little evidence of between-study effect heterogeneity for each SNP (rs10816625: Cochran's Q *P*-value = 0.48, *I*^2^ = 0; rs13294895: Cochran's Q *P*-value = 0.86, *I*^2^ = 0; rs676256: Cochran's Q *P*-value = 0.27, *I*^2^ = 0.11). rs676256 is essentially uncorrelated with either rs10816625 or rs13294895 (rs676256|rs10816625: *r*^2^ = 2.5 × 10^−04^, *D*′ = 0.08; rs676256|rs13294895: *r*^2^ = 0.013, *D*′ = 0.31). rs10816625 and rs13294895, which are within 103 bp of each other, lie in the same LD block (*D*′ = 1). The risk alleles rarely occur together: analysis of computationally phased genotype data estimated only 160 haplotypes carrying the risk alleles of both rs10816625 and rs13294895 from a total of over 183 000, corresponding to an estimated population frequency of 0.09% (compared with 1.2% expected under equilibrium). However, given the relative rarity of the risk alleles, there is little correlation between the SNPs (*r*^2^ = 0.014). SNPs rs10816625 and rs13294895 were uncorrelated with any other variant at *r*^2^ ≥ 0.8.

In Asians, rs10816625 was notable for being the only SNP that showed evidence of association with breast cancer risk, albeit not at genome-wide levels of significance (OR = 1.12 [1.06–1.18]; *P* = 2.77 × 10^−05^; Fig. 1E and Table 1; Supplementary Material, Table S2B). SNP rs10816625 has a relatively low minor-allele frequency (MAF; 6%) in European populations but is common in Asian populations (MAF averaged across controls from nine Asian studies = 38%). There was no evidence of inter-study heterogeneity for rs10816625 in the contributing Asian studies (Cochran's Q *P*-value = 0.51, *I*^2^ = 0). Although SNPs rs676256 (OR = 0.94 [0.82–1.06]; *P* = 0.3; Table 1), rs865686 (OR = 0.93 [0.84–1.02]; *P* = 0.13) and rs13294895 (OR = 1.04 [0.89–1.21]; *P* = 0.66) were not significantly associated with breast cancer risk in the Asian studies, their OR estimates were consistent with those of European women; power to detect associations of these SNPs was low because the minor allele frequencies were much lower than for Europeans. No SNPs were significantly associated with breast cancer risk in the African studies (Supplementary Material, Table S2C).

All three SNPs were associated with ER-positive (rs10816625: OR = 1.14 [1.09–1.19], *P* = 2.39 × 10^−08^; rs13294895: OR = 1.11 [1.08–1.14], *P* = 3.54 × 10^−12^; rs676256: OR = 0.87 [0.85–0.89], *P* = 1.66 × 10^−30^; Table 2) but not ER-negative (rs10816625: OR = 1.04 [0.96–1.13], *P* = 0.29, *P*_het_ = 0.05; rs13294895: OR = 1.03 [0.98–1.08], *P* = 0.25, *P*_het_ = 0.003; rs676256: OR = 0.98 [0.94–1.02], *P* = 0.31, *P*_het_ = 2.08 × 10^−08^; Table 2) breast cancer in subjects with European ancestry. A similar pattern was observed for progesterone receptor (PR) expression, with the exception that SNP rs676256 also showed a nominally significant association with PR-negative tumours (OR = 0.95 [0.91–0.98], *P* = 0.002; Table 2). Because tumour ER and PR status are strongly correlated, we modelled ER and PR co-expression using polytomous logistic regression. This revealed a similar association between rs676256 and risk of ER-positive/PR-positive breast cancer (OR = 0.87 [0.84–0.89]; *P* = 1.33 × 10^−24^; Table 3), ER-positive/PR-negative breast cancer (OR = 0.90 [0.86–0.95]; *P* = 1.20 × 10^−04^) and ER-negative/PR-positive breast cancer (OR = 0.89 [0.80–1.00]; *P* = 0.04). We further explored the association of rs676256 with ER-negative/PR-positive breast cancer using case-only analysis for PR, adjusted for ER (*P* = 0.06). SNP rs10816625 was significantly associated with only ER-positive/PR-positive breast cancer; rs13294895 was significantly associated with ER-positive/PR-positive breast cancer and nominally associated with ER-positive/PR-negative disease (Table 3).

**Table 2.** Association of rs10816625, rs13294895 and rs676256 with risk of breast cancer in European and Asian women stratified by ER status, PR status and HER2 status

Locus	Population	Controls	Cases	OR^a^	95% CI	*P*-value^b^	OR^a^	95% CI	*P*-value^b^	*P*_het_^c^
	Caucasian			ER+ tumours			ER− tumours			
rs10816625		41 324	25 851 | 6128	1.14	1.09–1.19	2.39 × 10^−08^	1.04	0.96–1.13	0.29	0.05
rs13294895		41 323	25 851 | 6130	1.11	1.08–1.14	3.54 × 10^−12^	1.03	0.98–1.08	0.25	0.003
rs676256		41 324	25 847 | 6128	0.87	0.85–0.89	1.66 × 10^−30^	0.98	0.94–1.02	0.31	2.08 × 10^−08^
				PR+ tumours			PR− tumours			
rs10816625		41 618	19 207 | 8470	1.16	1.10–1.22	1.36 × 10^−08^	1.06	0.99–1.13	0.11	0.02
rs13294895		41 617	19 207 | 8472	1.11	1.08–1.15	1.74 × 10^−10^	1.05	1.00–1.10	0.03	0.01
rs676256		41 619	19 207 | 8472	0.87	0.84–0.89	2.15 × 10^−27^	0.95	0.91–0.98	0.002	2.73 × 10^−06^
				HER2− tumours			HER2+ tumours			
rs10816625		31 756	12 872 | 2503	1.10	1.04–1.17	0.002	1.21	1.08–1.35	9.66 × 10^−04^	0.09
rs13294895		31 755	12 874 | 2503	1.10	1.06–1.14	3.29 × 10^−06^	1.07	1.00–1.16	0.06	0.53
rs676256		31 756	12 869 | 2502	0.87	0.85–0.90	2.75 × 10^−16^	0.92	0.87–0.98	0.008	0.14
	Asian			ER+ tumours			ER− tumours			
rs10816625		6622	3183 | 1547	1.13	1.06–1.21	1.30 × 10^−04^	1.14	1.05–1.24	0.002	0.84
rs13294895		6624	3183 | 1546	1.04	0.87–1.26	0.65	0.92	0.71–1.18	0.5	0.25
rs676256		6624	3184 | 1547	0.94	0.80–1.10	0.42	0.98	0.80–1.19	0.82	0.76
				PR+ tumours			PR− tumours			
rs10816625		5733	2711 | 1621	1.12	1.04–1.20	0.0012	1.15	1.06–1.25	5.45 × 10^−04^	0.5
rs13294895		5753	2711 | 1621	1.04	0.85–1.27	0.72	0.98	0.77–1.25	0.88	0.55
rs676256		5735	2712 | 1621	1.01	0.86–1.19	0.89	0.85	0.69–1.05	0.14	0.15
				HER2– tumours			HER2+ tumours			
rs10816625		3852	1058 | 785	1.17	1.05–1.30	0.0032	1.17	1.04–1.32	0.01	0.78
rs13294895		3853	1057 | 784	1.00	0.75–1.33	0.98	1.03	0.73–1.43	0.88	0.81
rs676256		3853	1058 | 785	1.00	0.80–1.26	0.98	0.87	0.66–1.16	0.34	0.27

^a^Stratum-specific ORs estimated using polytomous logistic regression.

^b^Stratum-specific *P*-values computed using Wald tests.

^c^*P*-value for heterogeneity in effect estimates between strata calculated using case-only logistic regression.

**Table 3.** Association of rs10816625, rs13294895 and rs676256 with risk of breast cancer in European women stratified by combined ER/PR status

Locus	Controls	Cases	ER/PR	OR^a^	95% CI	*P*-value^b^	*P*_het_^c^
rs10816625	38 144	17 132	ER+/PR+	1.17	1.11–1.24	4.76 × 10^−09^	
		3380	ER+/PR−	1.06	0.96–1.18	0.27	
		714	ER−/PR+	1.12	0.90–1.38	0.30	
		4436	ER−/PR−	1.07	0.98–1.18	0.12	0.03
rs13294895	38 143	17 132	ER+/PR+	1.13	1.09–1.16	6.38 × 10^−08^	
		3380	ER+/PR−	1.07	1.01–1.15	0.03	
		714	ER−/PR+	1.00	0.87–1.15	0.97	
		4438	ER−/PR−	1.05	0.99–1.11	0.12	0.01
rs676256	38 144	17 128	ER+/PR+	0.87	0.84–0.89	1.33 × 10^−24^	
		3380	ER+/PR−	0.90	0.86–0.95	1.20 × 10^−04^	
		714	ER−/PR+	0.89	0.80–1.00	0.04	
		4436	ER−/PR−	0.98	0.94–1.03	0.47	4.01 × 10^−06^

^a^Stratum-specific ORs estimated using separate logistic regression models comparing cases from each ER/PR combination with all controls.

^b^Stratum-specific *P*-values computed using Wald tests.

^c^*P*-value from χ^2^-test of heterogeneity of odds ratios.

There was little evidence for heterogeneity in the effects conferred by SNPs rs10816625, rs13294895 and rs676256 according to human epidermal growth factor receptor 2 (HER2) expression (Table 2). We also observed no evidence of heterogeneity in effects conferred by rs10816625 according to either tumour ER or PR status in subjects with Asian ancestry (Table 2).

Because all three SNPs reported in our fine-mapping analysis of Europeans were primarily associated with ER-positive, but not ER-negative tumours, we restricted further stratified analyses of additional breast cancer risk factors to cases with ER-positive disease. However, the results from analyses of all breast cancers combined and from ER-negative breast cancers are presented in Supplementary Material, Tables S4–S7. In Europeans, but not Asians, the effect of rs10816625 was stronger in cases with node-negative (OR = 1.19 [1.12–1.25], *P* = 4.55 × 10^−09^; Table 4) than in those with node-positive disease (OR = 1.07 [0.99–1.14], *P* = 0.07, *P*_het_ = 5.98 × 10^−03^; Table 4). There was no significant evidence of interaction according to tumour morphology (Table 5). We observed evidence of a linearly increasing trend in the OR by grade for rs10816625 in Asians only (*P*_trend_ = 4.91 × 10^−04^; Table 6). We previously reported a trend in per-allele OR for rs865686 with increasing age at diagnosis in ER-positive breast cancer, with a stronger association at younger ages (21). Here we report that the same was true for rs676256 in women of European ancestry (*P*_trend_ = 0.02; Table 7); we saw no compelling evidence of a similar age interaction for rs10816625 or rs13294895 (Table 7). Because the 9q31.2 breast cancer locus was initially discovered in a study enriched for bilateral and familial cases we estimated ORs for each SNP in sporadic, familial and bilateral cases (Supplementary Material, Table S8). There were no statistically significant differences in ORs between sporadic and either bilateral or familial cases.

**Table 4.** Association of rs10816625, rs13294895 and rs676256 with risk of ER-positive breast cancer stratified by lymph node status

Locus	Population	Controls	Cases	OR^a^	95% CI	*P*-value^b^	OR^a^	95% CI	*P*-value^b^	*P*_het_^c^
	Caucasian			Node-negative tumours			Node-positive tumours			
rs10816625		40 313	13 093 | 8235	1.19	1.12–1.25	4.55 × 10^−09^	1.07	0.99–1.14	0.07	5.98 × 10^−03^
rs13294895		40 313	13 093 | 8235	1.10	1.06–1.15	1.36 × 10^−07^	1.13	1.08–1.18	7.90 × 10^−08^	0.43
rs676256		40 313	13 090 | 8234	0.86	0.84–0.89	5.42 × 10^−22^	0.90	0.87–0.93	1.17 × 10^−08^	0.04
	Asian			Node-negative tumours			Node-positive tumours			
rs10816625		4741	1084 | 740	1.13	1.02–1.25	0.02	1.11	0.98–1.24	0.03	0.77
rs13294895		4742	1083 | 740	1.16	0.88–1.53	0.29	1.07	0.78–1.49	0.66	0.72
rs676256		4742	1084 | 740	1.02	0.81–1.29	0.85	1.01	0.77–1.31	0.97	0.94

^a^Stratum-specific ORs estimated using polytomous logistic regression.

^b^Stratum-specific *P*-values computed using Wald tests.

^c^*P*-value for heterogeneity in effect estimates between strata calculated using case-only logistic regression.

**Table 5.** Association of rs10816625, rs13294895 and rs676256 with ER-positive breast cancer stratified by morphology

Locus	Population	Controls	Cases	OR^a^	95% CI	*P*-value^b^	OR^a^	95% CI	*P*-value^b^	*P*_het_^c^
	Caucasian			Ductal tumours			Lobular tumours			
rs10816625		34 151	15 007 | 3199	1.12	1.05–1.18	1.25 × 10^−04^	1.17	1.06–1.30	1.91 × 10^−03^	0.35
rs13294895		34 149	15 007 | 3199	1.10	1.06–1.14	5.51 × 10^−07^	1.12	1.05–1.20	5.43 × 10^−04^	0.42
rs676256		34 150	15 004 | 3199	0.88	0.85–0.90	1.16 × 10^−18^	0.84	0.80–0.89	5.64 × 10^−10^	0.17
	Asian			Ductal tumours			Lobular tumours			
rs10816625		3852	1800 | 85	1.12	1.03–1.22	8.50 × 10^−03^	1.29	0.94–1.77	0.11	0.32
rs13294895		3853	1799 | 85	1.16	0.92–1.46	0.22	1.16	0.47–2.87	0.74	0.96
rs676256		3853	1800 | 85	0.91	0.74–1.12	0.38	1.58	0.84–2.96	0.16	0.13

^a^Stratum-specific ORs estimated using polytomous logistic regression.

^b^Stratum-specific *P*-values computed using Wald tests.

^c^*P*-value for heterogeneity in effect estimates between strata calculated using case-only logistic regression.

**Table 6.** Association of rs10816625, rs13294895 and rs676256 with ER-positive breast cancer stratified by tumour grade

Locus	Population	Controls	Cases^a^	Grade	OR^b^	95% CI	*P*-value^c^	*P*_trend_^d^
rs10816625	Caucasian	39 762	5233	1	1.16	1.07–1.26	4.26 × 10^−04^	
			11 432	2	1.14	1.07–1.16	1.91 × 10^−05^	
			4 655	3	1.09	1.00–1.19	0.05	0.26
rs13294895		39 763	5233	1	1.08	1.02–1.14	0.005	
			11 432	2	1.11	1.07–1.16	4.35 × 10^−08^	
			4655	3	1.10	1.04–1.17	5.33 × 10^−04^	0.60
rs676256		39 763	5232	1	0.88	0.84–0.92	2.27 × 10^−09^	
			11 429	2	0.87	0.84–0.89	1.13 × 10^−19^	
			4655	3	0.88	0.84–0.92	6.40 × 10^−08^	0.96
rs10816625	Asian	4488	331	1	1.02	0.86–1.20	0.85	
			961	2	1.10	0.98–1.22	0.09	
			427	3	1.42	1.22–1.65	4.88 × 10^−06^	4.91 × 10^−04^
rs13294895		4489	331	1	0.85	0.51–1.43	0.54	
			961	2	1.17	0.86–1.57	0.32	
			427	3	1.25	0.84–1.87	0.27	0.46
rs676256		4489	331	1	1.07	0.75–1.53	0.72	
			961	2	1.04	0.81–1.33	0.75	
			427	3	0.68	0.46–1.02	0.06	0.06

^a^Maximum total number of cases for each stratum.

^b^Stratum-specific ORs estimated using polytomous logistic regression.

^c^Stratum-specific *P*-values computed using Wald tests.

^d^*P*-value for linear trend in effect estimates across strata calculated using case-only logistic regression.

**Table 7.** Association of rs10816625, rs13294895 and rs676256 with ER-positive breast cancer in Europeans, stratified by age at diagnosis

Locus	Controls	Cases^a^	Age Group	OR^b^	95% CI	*P*-value^c^	*P*_trend_^d^
rs10816625	30 239	988	<40	1.18	0.99–1.41	0.06	
		3858	40–49	1.20	1.09–1.32	1.39 × 10^−4^	
		6865	50–59	1.14	1.06–1.23	6.93 × 10^−4^	
		6173	60–69	1.13	1.04–1.22	0.003	
		2679	≥70	1.10	0.99–1.24	0.08	0.25
rs13294895	30 239	988	<40	1.07	0.95–1.20	0.26	
		3858	40–49	1.15	1.08–1.22	7.84 × 10^−06^	
		6865	50–59	1.12	1.07–1.18	2.42 × 10^−06^	
		6173	60–69	1.11	1.05–1.16	6.70 × 10^−05^	
		2679	≥70	1.04	0.97–1.12	0.25	0.13
rs676256	30 240	987	<40	0.89	0.81–0.98	0.02	
		3858	40–49	0.82	0.78–0.86	5.13 × 10^−14^	
		6864	50–59	0.86	0.83–0.90	1.03 × 10^−13^	
		6171	60–69	0.89	0.86–0.93	7.56 × 10^−08^	
		2679	≥70	0.92	0.87–0.98	0.006	0.02

^a^Maximum total number of cases for each stratum.

^b^Stratum-specific ORs estimated using polytomous logistic regression.

^c^Stratum-specific *P*-values computed using Wald tests.

^d^*P*-value for linear trend in effect estimates across strata calculated using case-only logistic regression.

**Figure 2.**
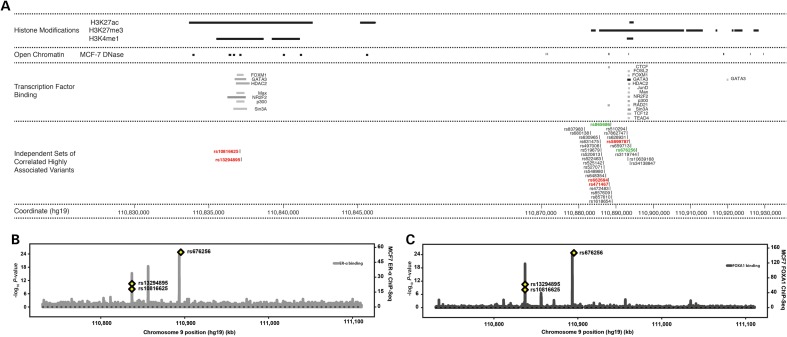
Plots of genomic annotations with putative functional significance at the 9q31.2 fine-mapping region. (**A**) Publically available histone modification, DNase hypersensitivity and transcription factor binding data from MCF7 cells were mapped on to the breast cancer associated regions identified by fine-mapping. For SNPs rs10826625 and rs13294895, the iCHAVs were defined as SNPs having *r*^2^ ≥ 0.8 with either SNP; for rs676256 it was defined as all SNPs with *r*^2^ ≥ 0.8 and likelihood ratios >1:100 relative to rs676256. There were no other SNPs in the iCHAVs for rs10816625 and rs13294895. The rs676256 iCHAV comprised 28 SNPs. SNPs whose identifiers are shown in red type were of putative functional significance (see Materials and Methods). Where the lead SNP was not deemed to be of putative functional significance, it is indicated in green, as is the index 9q31.2 SNP, rs865686. (**B**) Regional binding profiles for ER-α in MCF7 cells shown plotted across the fine-mapping region using data from (31). The locations of the lead SNPs are indicated with yellow diamonds. (**C**) Regional binding profiles for FOXA1 in MCF7 cells shown plotted across the fine-mapping region using data from (31). The locations of the lead SNPs are indicated with yellow diamonds.

In an effort to identify putative causal variants underlying each of the three associations, we performed a bioinformatic analysis. We used data from the ENCODE project (24) and elsewhere (25) to explore the co-localisation of the association signals with features indicative of functional genomic elements in breast cancer models, including evidence of transcription factor binding, DNase hypersensitivity and relevant histone modification marks. Both SNPs rs10816625 and rs13294895 localise to a region of putative regulatory significance in MCF7 cells, demarcated by histone H3 lysine 27 acetylation (H3K27ac) and histone H3 lysine 4 mono-methylation (H3K4me1), both of which are characteristic features of active enhancers (Fig. 2A) (26,27). There was less evidence for either histone modification mark in human mammary epithelial cells (HMEC; not shown). Both SNPs are located directly under the binding sites for a number of breast cancer-relevant transcription factors, including forkhead box M1 (FOXM1) and GATA binding protein 3 (GATA3; Fig. 2A) (28,29).

To reduce the number of candidate functional polymorphisms for the rs676256 iCHAV, we applied a heuristic scoring system to prioritise variants that localise to regions with cistromic and epigenetic activity (30). We identified three variants in this iCHAV that co-localise with potentially relevant genomic features (Fig. 2A). Specifically, all three variants lie in regions of open chromatin in MCF7 cells (Fig. 2A). SNPs rs662694 (110 887 996 bp; OR = 0.88 [0.87–0.90]; *P* = 5.64 × 10^−25^) and rs471467 (110 888 113 bp; OR = 0.88 [0.87–0.90]; *P* = 3.30 × 10^−25^) localise to a CTCF binding site, which suggests insulator activity, while insertion–deletion (indel) polymorphism rs5899787 (110 893 551–2 bp; OR = 0.88 [0.87–0.90]; *P* = 1.67 × 10^−24^) lies in a region with features of a poised enhancer, namely enrichment of histone H3 lysine 27 trimethylation (H3K27me3) and has evidence of FOXM1 and GATA3 binding in MCF7 cells (Fig. 2A).

Estrogen receptor-α (ER-α) and forkhead box A1 (FOXA1) are key drivers of ER-positive breast cancer. Because there are currently limited ENCODE data on either of these factors, we explored their binding at the 9q31.2 susceptibility locus in MCF7 cells using data from Hurtado *et al.* (31). We found that the three lead SNPs localise to binding sites for both transcription factors (Fig. 2B and C). SNPs rs10816625 and rs13294895 map directly under ER-α and FOXA1 binding peaks which co-localise to the putative active enhancer described above. rs5899787, from the rs676256 iCHAV, also maps directly under an ER-α and FOXA1 binding peak; none of the other SNPs in the rs676256 iCHAV map to this, or any other ER-α and FOXA1 peaks.

**Figure 3.**
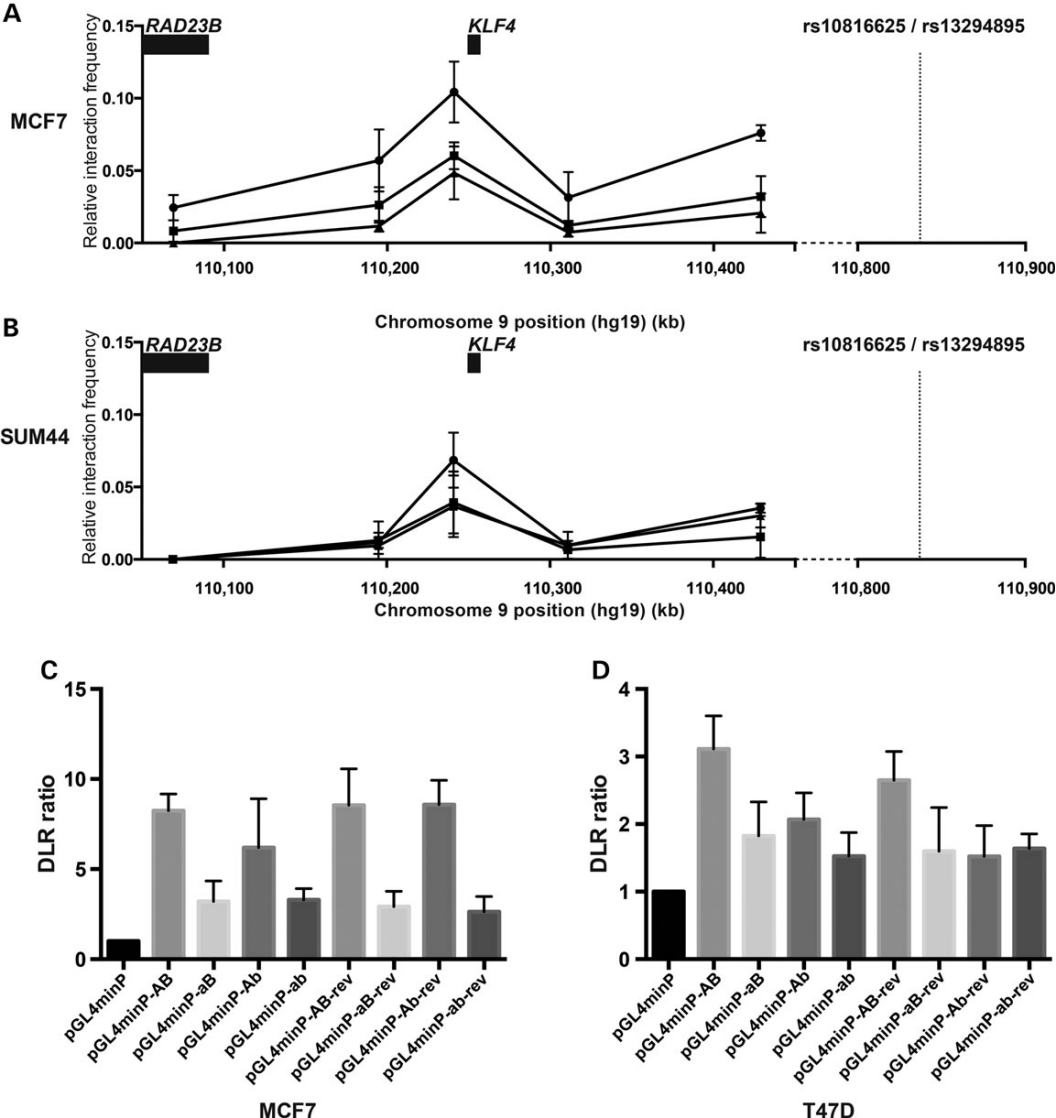
Chromatin conformation capture and reporter gene analysis of SNPs rs10816625 and rs13294895. (**A**) Chromatin interaction data from *Hin*dIII 3C libraries generated using MCF7 cells that indicates interactions between a fragment containing rs10816625 and rs13294895 (dashed line) and fragments surrounding *KLF4*. Results from three replicate libraries are plotted; each quantitative PCR reaction was performed in triplicate. Error bars represent standard mean errors. (**B**) Chromatin interaction data from *Hin*dIII 3C libraries generated using SUM44 cells. (**C**) Dual luciferase assays for reporter constructs containing the common alleles of both rs10816625 and rs13294895 (pGL4minP-AB), risk allele of rs10816625 (pGL4minP-aB), risk allele of rs13294895 (pGL4minP-Ab) and risk alleles of both SNPs (pGL4minP-ab) transiently transfected into MCF7 cells. Ratios were normalised to a minimal promoter construct (pGL4minP). Each transfection was repeated five times and constructs were generated in both forward and reverse orientations. (**D**) Dual luciferase assays for reporter constructs containing the common alleles of both rs10816625 and rs13294895 (pGL4minP-AB), risk allele of rs10816625 (pGL4minP-aB), risk allele of rs13294895 (pGL4minP-Ab) and risk alleles of both SNPs (pGL4minP-ab) transiently transfected into T47D cells.

A recent integrative analysis of data from The Cancer Genome Atlas suggested that the original 9q31.2 risk locus influences transcript levels of *KLF4* (32). We investigated, using chromosome conformation capture (3C) in *Hin*dIII digested MCF7 (Fig. 3A) and SUM44 (Fig. 3B) 3C libraries, whether the locus containing SNPs rs10816625 and rs13294895 also interacts with *KLF4* through long-range chromatin interaction. We detected elevated interaction frequencies between *Hin*dIII fragments containing SNPs rs10816625 and rs13294895 and those containing *KLF4*; interactions with *Hin*dIII fragments either side of *KLF4* were lower in comparison. Moreover no interaction was detected between the fragment containing SNPs rs10816625 and rs13294895 with *RAD23B*.

To determine whether either locus had enhancer activity we performed a series of dual luciferase assays using a minimal promoter vector, pGL4minP. To explore the rs10816625/rs13294895 locus we inserted a 1 kb fragment containing the common alleles of both variants, plus flanking DNA, into pGL4minP (pGL4minP-AB). We observed an increased level of activity of the minimal promoter in the pGL4minP-AB construct relative to pGL4minP in both MCF7 (8.2-fold increase; *P* = 6.12 × 10^−05^; Fig. 3C) and T47D cells (3.1-fold increase; *P* = 6.66 × 10^−04^; Fig. 3D). To determine whether the risk alleles of rs10816625 and rs13294895 disrupted this enhancer activity we generated three additional constructs, carrying a single risk allele of either rs10816625 (pGL4minP-aB) or rs13294895 (pGL4minP-Ab), or carrying risk alleles of both SNPs (pGL4minP-ab). We observed significant evidence for a difference in the means of the dual luciferase ratios of these constructs in MCF7 and T47D cells (*P* < 7 × 10^−04^; Fig. 3C and D). In T47D cells we found a statistically significant difference between pGL4minP-AB and either pGL4minP-aB (*P* = 5.45 × 10^−03^), pGL4minP-Ab (*P* = 0.04) or pGL4minP-ab (*P* = 4.97 × 10^−04^; Fig. 3D). In MCF7 cells there was a statistically significant difference between pGL4minP-AB and pGL4minP-aB (*P* = 6.62 × 10^−05^), but not pGL4minP-Ab (Fig. 3C). There was no significant difference between the construct containing both risk alleles and constructs containing one risk allele in T47D cells (Fig. 3D). We performed a similar series of analyses to explore the putative poised enhancer centred on SNP rs5899787. Relative to pGL4minP, we observed a reduction in reporter gene expression but saw no evidence to support an allele-specific effect (data not shown).

## Discussion

In a combined analysis of data from 50 case–control studies comprising more than 100 000 women, we have refined the localisation of the breast cancer association signal on chromosome 9q31.2 to a set of 28 highly correlated variants in a 14.5 kb region in which SNP rs676256 was the most strongly associated variant. Furthermore we have demonstrated the presence of two novel independent susceptibility alleles at 9q31.2, SNPs rs10816625 and rs13294895, both of which are strong candidates to be causal variants. Breast cancer is a heterogeneous disease comprising multiple subtypes that can be classified according to histological, immunophenotypic and molecular characteristics. Although the majority of known breast cancer susceptibility loci are preferentially associated with ER-positive tumours (33), a number of recent subtype-specific studies have detected genetic associations unique to ER-negative tumours, suggesting distinct underlying aetiologies for each subtype (17,34,35). The index 9q31.2 breast cancer susceptibility association, demarcated by SNP rs865686 (7), was largely restricted to ER-positive breast cancer (21) and this was confirmed for rs676256 in the European samples analysed in this study. SNPs rs10816625 and rs13294895 were also associated with ER-positive, but not ER-negative, breast cancer in Europeans, albeit with more modest statistical evidence of heterogeneity than for rs672656.

The majority of susceptibility loci for breast and other cancers have been detected using studies of predominantly European ancestry. However, confirmation of associations in populations with different ethnicity from those used for discovery can add weight to their validity (36). Approximately 10% of the samples genotyped in our fine-mapping study were from subjects of Asian ancestry. In Asians, rs10816625 had a higher MAF than in Europeans and was the only SNP that was significantly associated with breast cancer risk; the OR was similar to that in Europeans. Neither rs676256 nor rs13294895 were significantly associated with risk in Asians, but the MAFs were much smaller than in Europeans and the ORs did not differ by ethnicity. SNP rs10816625 resides on a strong hotspot of recombination in Europeans and exhibits low pairwise correlation with all but two other SNPs, each of which has a *P*-value for association with breast cancer several orders of magnitude larger than that of rs10816625. These observations provide evidence that rs10816625 was causally associated with breast cancer.

The third breast cancer susceptibility SNP that we detected, rs13294895, localises to within ∼100 bp of rs10816625. Analysis of computationally phased haplotypes indicates that their risk alleles rarely occur together, consistent with having arisen independently on the same ancestral haplotype with little subsequent recombination.

We used bioinformatic annotation of the regions demarcated by SNPs rs10816625, rs13294895 and rs676256 to identify a set of variants that had putative regulatory potential and, as such, were candidates to be the causal alleles underlying the observed associations. SNPs rs10816625 and rs13294895 localise to a region with a histone modification signature that suggests it is an active enhancer in MCF7 cells. We also saw evidence that supports binding of ER-α, FOXA1 and GATA3 at this locus, directly over the sites of rs10816625 and rs13294895. ER-α is an established driver of luminal breast cancer and FOXA1 is a pioneer factor that physically interacts with compacted chromatin, facilitating binding of ER-α, and is necessary for ER-α mediated transcription (31,37). GATA3 is thought to play a key role in making enhancer elements accessible to ER-α and its expression is highly correlated with both ER-α and FOXA1 in breast tumours (38,39). Of note, Cowper-Sal·lari *et al* have recently demonstrated that breast cancer susceptibility loci are enriched for ER-α and FOXA1 binding events (40). Our *in vitro* data support the hypothesis that this locus possesses enhancer activity and indicate that the risk alleles of rs10816625 and rs13294895 can diminish its activity, indicating that these are independent risk susceptibility variants acting through the same mechanism.

Li *et al.* have recently suggested the original 9q31.2 breast cancer susceptibility locus acts via regulation of the transcription factor KLF4 (32). In their article these authors identified *KLF4* as the target of the 9q31.2 locus on the basis of a trans-eQTL analysis in which they first identified the set of eQTL genes associated with rs471467 (a perfect proxy for rs865686) and then looked for enrichment of transcription factor binding sites within ENCODE defined enhancer elements of these genes. We have demonstrated an excess of long-range chromatin interactions between the rs10816625/rs13294895 region and the *KLF4* gene locus. Our results and those of Li *et al.* suggest therefore that *KLF4* is the target of multiple 9q31.2 breast cancer susceptibility SNPs. In contrast to recent eQTL analysis by Li and colleagues implicating *RAD23B* as the target of the prostate cancer susceptibility SNP rs817826, we found no evidence that these breast cancer SNPs interacted with *RAD23B* (41). KLF4 has both oncogenic and tumour suppressive roles depending on the tissue in which it is expressed (42). It is thought to be expressed at low levels in normal breast epithelium, but is overexpressed in a large proportion of both ductal carcinoma in situ and invasive breast cancer (43). Our reporter assays targeting the rs10816625/rs13294895 SNPs suggest that lower levels of expression of KLF4 are associated with increased breast cancer risk.

In contrast to the rs10816625/rs13294895 locus, refinement of the association signal at the rs676256 locus was complicated by the large number of variants in high LD with the lead SNP. Of the 28 highly correlated variants in this iCHAV, analysis of ENCODE data identified three that fall into two distinct functional regions. SNPs rs662694 and rs471467 localise to a predicted insulator region, defined by CTCF binding and H3K27me3 marks (44). SNP rs5899787 was located in a region that shared similar functionally significant features to those of the rs10816625/rs13294895 locus. It localises directly to a second site of strong ER-α and FoxA1 co-localisation and had strong evidence of GATA-3 binding in the ENCODE data. Our data suggested that a construct containing the common allele of rs5899787 suppressed the activity of the minimal promoter in our reporter gene system, but we saw no evidence for an allele-specific effect. Further work will be required to determine the identity and mode of action of the causative variant (or variants) at this locus.

Including the variants identified in our study, 81 common germline polymorphisms conferring susceptibility to breast cancer have now been identified. Our study, and those of others, demonstrate the power of fine-mapping in large studies both for the detection of novel independent susceptibility SNPs and determining a minimal set of likely causal variants (15,16).

## Materials and Methods

### Sample selection

Samples (*n* = 103 991) were selected from 52 studies participating in BCAC and genotyped as part of the COGS project (9). Most contributing studies were either population or hospital-based case–control studies, while some were nested in cohorts or selected for family history, age or tumour characteristics. Full details of contributing studies can be found in Supplementary Material, Table S1. Four studies, Demokritos (DEMOKRITOS), Ohio State University (OSU), Städtisches Klinikum Karlsruhe Deutsches Krebsforschungszentrum Study (SKKDKFZS) and the Roswell Park Cancer Institute Study (RPCI) were genotyped as part of the Triple Negative Breast Cancer Case–control Consortium, but are analysed here in their component studies. Analyses were restricted to cases with invasive breast cancer. All analyses reported were stratified according to ancestry of the study participants, categorised as having predominantly European (*n* = 43 160 cases; 42 600 controls), Asian (*n* = 5795 cases; 6624 controls) or African ancestry (*n* = 1046 cases; 932 controls), determined by a principal components analysis of 37 000 uncorrelated SNPs ancestry-informative markers, described elsewhere (9). All BCAC studies had local ethical approval.

### Genotyping and quality control

A total of 447 fine-mapping SNPs were selected to interrogate the 9q31.2 locus. The fine-mapping region was defined as the region that included including all SNPs correlated with the index SNP, rs865686, at *r*^2^ > 0.1. For genotyping we first selected all SNPs with an Illumina Design Score >0.8 and *r*^2^ with rs865686 >0.1. We then selected an additional set of SNPs designed to tag all remaining SNPs in the interval at *r*^2^ > 0.9. Genotyping was performed using a custom-designed International Collaborative Oncology Gene-environment Study (iCOGS) genotyping array (Illumina, San Diego, CA). The iCOGS array comprised assays for 211 155 SNPs, primarily selected for replication analysis of loci putatively associated with breast, ovarian or prostate cancer and for fine-mapping of the known susceptibility loci for these cancers. Full details of the iCOGS array design, sample handling and post-genotyping QC processes are described in-depth elsewhere (9). Briefly, samples were excluded from the analytic dataset for any of the following reasons: gender discordance according to array data, call rate <95%, excess heterozygosity (*P* < 1 × 10^−06^), individuals not concordant with previous genotyping, discordant duplicate pairs, within-study duplicates with discordant phenotype data, or inter-study duplicates, first degree relatives, phenotypic exclusions and concordant replicates. Multi-dimensional scaling was used to infer ethnicity; individuals with greater than 15% mixed ancestry were excluded from analyses. Clustering of significantly associated, directly-genotyped SNPs was verified by manual inspection of genotype cluster plots (Supplementary Material, Fig. S1). Of the 447 target-SNPs selected for fine-mapping, 424 passed post-genotyping quality control measures; we excluded six SNPs that were monomorphic in Europeans and a further six that showed strongly significant deviation of genotype frequencies from Hardy–Weinberg proportions in controls (*P* < 1 × 10^−04^).

### Bioinformatics

We used publically available DNase hypersensitivity, transcription factor binding and histone modification ChIP-seq data from the ENCODE project (24) and elsewhere (27,31) to overlay functional annotations on the fine-mapping region and investigate enrichment of functional elements at associated loci. For the rs676256 locus we first identified a subset of polymorphisms that had *r*^2^ ≥ 0.8 with the lead SNP and then filtered the putative functional significance of variants by applying a heuristic score using RegulomeDB (http://regulome.stanford.edu/) to prioritise candidate functional variants prior to further investigation.

### Quantitative 3C

MCF7 and SUM44 3C libraries were generated using 2 × 10^7^ cells fixed with 2% paraformaldehyde for 5 min. 3C was carried out using the digestion and ligation steps of a Hi-C protocol (45), replacing the biotin dNTP fill-in with the addition of 56.7 µl of water. A control 3C library was generated as previously described (46) using minimally overlapping BAC clones (Children's Hospital Oakland Research Institute, Oakland CA; Life Technologies, Carlsbad, CA, USA) which covered the *Hin*dIII fragments between rs10816625 and the target region, combined in equimolar amounts. To optimise the Taqman PCR reactions and normalise the data, we generated a standard curve using the control templates. Taqman PCR was carried out using Taqman Universal PCR Mastermix no UNG (Life Technologies, Carlsbad CA) with 250 ng of 3C library. Three separate 3C libraries were prepared for each cell-line, then from each library three quantitative PCR reactions were performed for each restriction fragment. Interactions between rs10816625/rs13294895 and target loci were expressed as relative interaction frequencies compared with the control BAC library standard curve. BAC libraries and primer sequences are available on request.

### Dual luciferase assays

DNA fragments containing either rs10816625 and rs13294895 or rs5899787 were cloned into the multiple cloning site of pGL4.23[luc2/minP] (Promega, Madison, WI). Site-directed mutagenesis with the Quickchange Lightning Site Directed Mutagenesis Kit (Agilent Technologies, Berkshire, UK) was used to create constructs containing all combinations of rs10816625/rs13294895 common and risk alleles (rs10286625 common/rs13294895 common, pGL4minP-AB; rs10286625 risk/rs13294895 common, pGL4minP-aB; rs10286625 common/rs13294895 risk, pGL4minP-Ab; rs10286625 risk/rs13294895 risk, pGL4minP-ab). In addition, we created reverse orientation constructs for each insert to verify orientation independence. The allelic status of each construct was confirmed by Sanger sequencing. PCR primers for cloning and site-directed mutagenesis are available on request. We used gBlocks Gene Fragments (Integrated DNA Technologies, Leuven, Belgium) to create constructs (pGL4minP-A and pGL4minP-a) for the common and risk alleles of the rs5899787 SNP.

MCF7 and T47D cells (ATCC, Middlesex, UK) were seeded at a density of 1.6 × 10^04^ cells per well of a 96-well plate and transfected with 50 ng of pGL4.23[luc2/minP] or cloned constructs and 50 ng of pGL4.74[hRluc/TK] (Promega) using XtremeGENE HP transfection reagent (Roche, Basel, Switzerland). Luciferase levels were measured using a Victor luminometer (PerkinElmer, Waltham, MI) after 24 h using the Dual-Glo Luciferase Assay System (Promega). All transfections were repeated five times.

### Statistics

Analysis of the association between each SNP and risk of breast cancer was performed using unconditional logistic regression assuming a log-additive genetic model, adjusted for study and ancestry-informative principal components (*n* = 7 for European studies; *n* = 2 for Asian and African studies). *P*-values were calculated using a one-degree of freedom likelihood-ratio test. We also estimated the effects of each heterozygote and minor-allele homozygote genotype relative to the common homozygote in a two-degrees-of-freedom model (Supplementary Material, Table S2). Forward stepwise logistic regression was used to explore whether additional loci in the fine-mapping region were independently associated with breast cancer risk. *I*^2^ statistics were used to assess heterogeneity of the RR estimates between studies at significantly associated loci. We conducted analyses of SNP associations by tumour receptor status, morphology, lymph node involvement, grade and age for the European and Asian ancestry studies using polytomous logistic regression. Tumour information in BCAC was collected as previously described (47). There were too few samples with African ancestry to conduct stratified analyses. We also considered a polytomous logistic regression model comprising all four possible combinations of ER and PR status. Case-only analyses of tumour receptor status, morphology and lymph node involvement were used to assess heterogeneity between disease subtypes. Case-only allelic logistic regression using number of copies of each minor allele as response variable was used to test for linear trends in OR by grade and age at diagnosis.

We used a *t*-test to assess the difference in mean dual luciferase ratios for reporter gene constructs. One-way analysis of variance was used to assess equality of means of log-transformed dual luciferase ratios. Homogeneity of variances was assessed using Bartlett's test and QQ-plots of standardised residuals were visually inspected for evidence of departure from those expected under a normal distribution.

Post-hoc comparison of group means was carried out using Tukey's HSD test. All statistical analyses were conducted using R (www.R-project.org/) and the Genotype Libraries and Utilities package (GLU; code.google.com/p/glu-genetics).

## Supplementary Material

Supplementary Material is available at *HMG* online.

## Funding

BCAC is funded by Cancer Research UK (C1287/A10118, C1287/A12014) and by the European Community's Seventh Framework Programme under grant agreement number 223175 (grant number HEALTH-F2-2009-223175). Funding for the iCOGS infrastructure came from: the European Community's Seventh Framework Programme under grant agreement no. 223175 (HEALTH-F2-2009-223175) (COGS), Cancer Research UK (C1287/A10118, C1287/A 10710, C12292/A11174, C1281/A12014, C5047/A8384, C5047/A15007, C5047/A10692 and C8197/A16565), the National Institutes of Health (CA128978) and Post-Cancer GWAS initiative (1U19 CA148537, 1U19 CA148065 and 1U19 CA148112—the GAME-ON initiative), the Department of Defence (W81XWH-10-1-0341), the Canadian Institutes of Health Research (CIHR) for the CIHR Team in Familial Risks of Breast Cancer, Komen Foundation for the Cure, the Breast Cancer Research Foundation, and the Ovarian Cancer Research Fund. The Australian Breast Cancer Family Study (ABCFS) was supported by grant UM1 CA164920 from the National Cancer Institute (USA). The ABCS study was supported by the Dutch Cancer Society (grants NKI 2007-3839; 2009 4363); BBMRI-NL, which is a Research Infrastructure financed by the Dutch government (NWO 184.021.007) and the Dutch National Genomics Initiative. The work of the BBCC was partly funded by ELAN-Fond of the University Hospital of Erlangen. The BBCS is funded by Cancer Research UK and Breakthrough Breast Cancer and acknowledges NHS funding to the NIHR Biomedical Research Centre, and the National Cancer Research Network (NCRN). E.S. is supported by NIHR Comprehensive Biomedical Research Centre, Guy's & St. Thomas’ NHS Foundation Trust in partnership with King's College London, UK. I.T. is supported by the Oxford Biomedical Research Centre. The BSUCH study was supported by the Dietmar-Hopp Foundation, the Helmholtz Society and the German Cancer Research Center (DKFZ). The CECILE study was funded by Fondation de France, Institut National du Cancer (INCa), Ligue Nationale contre le Cancer, Ligue contre le Cancer Grand Ouest, Agence Nationale de Sécurité Sanitaire (ANSES) Agence Nationale de la Recherche (ANR). The CGPS was supported by the Chief Physician Johan Boserup and Lise Boserup Fund, the Danish Medical Research Council and Herlev Hospital. The CNIO-BCS was supported by the Genome Spain Foundation, the Red Temática de Investigación Cooperativa en Cáncer and grants from the Asociación Española Contra el Cáncer and the Fondo de Investigación Sanitario (PI11/00923 and PI081120). The Human Genotyping-CEGEN Unit (CNIO) is supported by the Instituto de Salud Carlos III. The CTS was initially supported by the California Breast Cancer Act of 1993 and the California Breast Cancer Research Fund (contract 97–10500) and is currently funded through the National Institutes of Health (R01 CA77398). Collection of cancer incidence data was supported by the California Department of Public Health as part of the statewide cancer reporting program mandated by California Health and Safety Code Section 103885. H.A.C receives support from the Lon V Smith Foundation (LVS39420). The ESTHER study was supported by a grant from the Baden Württemberg Ministry of Science, Research and Arts. Additional cases were recruited in the context of the VERDI study, which was supported by a grant from the German Cancer Aid (Deutsche Krebshilfe). The GC-HBOC was supported by Deutsche Krebshilfe (107 352). The GENICA was funded by the Federal Ministry of Education and Research (BMBF) Germany grants 01KW9975/5, 01KW9976/8, 01KW9977/0 and 01KW0114, the Robert Bosch Foundation, Stuttgart, Deutsches Krebsforschungszentrum (DKFZ), Heidelberg, the Institute for Prevention and Occupational Medicine of the German Social Accident Insurance, Institute of the Ruhr University Bochum (IPA), Bochum, as well as the Department of Internal Medicine, Evangelische Kliniken Bonn gGmbH, Johanniter Krankenhaus, Bonn, Germany. The HEBCS was financially supported by the Helsinki University Central Hospital Research Fund, Academy of Finland (266528), the Finnish Cancer Society, and The Nordic Cancer Union and the Sigrid Juselius Foundation. Financial support for KARBAC was provided through the regional agreement on medical training and clinical research (ALF) between Stockholm County Council and Karolinska Institutet, The Swedish Cancer Society and the Gustav V Jubilee foundation. The KBCP was financially supported by the special Government Funding (EVO) of Kuopio University Hospital grants, Cancer Fund of North Savo, the Finnish Cancer Organizations, and by the strategic funding of the University of Eastern Finland. ‘kConFab’ is supported by a grant from the National Breast Cancer Foundation, and previously by the National Health and Medical Research Council (NHMRC), the Queensland Cancer Fund, the Cancer Councils of New South Wales, Victoria, Tasmania and South Australia, and the Cancer Foundation of Western Australia. LMBC is supported by the ‘Stichting tegen Kanker’ (232–2008 and 196–2010). Diether Lambrechts is supported by the FWO and the KULPFV/10/016-SymBioSysII. The MARIE study was supported by the Deutsche Krebshilfe e.V. [70-2892-BR I], the Hamburg Cancer Society, the German Cancer Research Center and the Federal Ministry of Education and Research (BMBF), Germany (01KH0402). MBCSG is supported by grants from the Italian Association for Cancer Research (AIRC) and by funds from the Italian citizens who allocated the 5/1000 share of their tax payment in support of the Fondazione IRCCS Istituto Nazionale Tumori, according to Italian laws (INT—Institutional strategic projects ‘5 × 1000’). The MCBCS was supported by the NIH grants CA128978, CA116167 and CA176785 and NIH Specialized Program of Research Excellence (SPORE) in Breast Cancer (CA116201), and the Breast Cancer Research Foundation and a generous gift from the David F. and Margaret T. Grohne Family Foundation and the Ting Tsung and Wei Fong Chao Foundation. MCCS cohort recruitment was funded by VicHealth and Cancer Council Victoria. The MCCS was further supported by Australian NHMRC grants 209057, 251553 and 504711 and by infrastructure provided by Cancer Council Victoria. The MEC was support by NIH grants CA63464, CA54281, CA098758 and CA132839. The work of MTLGEBCS was supported by the Quebec Breast Cancer Foundation, the Canadian Institutes of Health Research for the ‘CIHR Team in Familial Risks of Breast Cancer’ program—grant # CRN-87521 and the Ministry of Economic Development, Innovation and Export Trade—grant # PSR-SIIRI-701. The NBCS was supported by grants from the Norwegian Research council, 155218/V40, 175240/S10 to A.L.B.D., FUGE-NFR 181600/V11 to V.N.K. and a Swizz Bridge Award to A.L.B.D. The NBHS was supported by NIH grant R01CA100374. Biological sample preparation was conducted by the Survey and Biospecimen Shared Resource, which is supported by P30 CA68485. The OBCS was supported by research grants from the Finnish Cancer Foundation, the Academy of Finland (grant number 250083, 122715 and Center of Excellence grant number 251314), the Finnish Cancer Foundation, the Sigrid Juselius Foundation, the University of Oulu, the University of Oulu Support Foundation and the special Governmental EVO funds for Oulu University Hospital-based research activities. OFBCR was supported by grant UM1 CA164920 from the National Cancer Institute (USA). The ORIGO study was supported by the Dutch Cancer Society (RUL 1997–1505) and the Biobanking and Biomolecular Resources Research Infrastructure (BBMRI-NL CP16). The PBCS was funded by Intramural Research Funds of the National Cancer Institute, Department of Health and Human Services, USA. The pKARMA study was supported by Märit and Hans Rausings Initiative Against Breast Cancer. The RBCS was funded by the Dutch Cancer Society (DDHK 2004-3124, DDHK 2009-4318). The SASBAC study was supported by funding from the Agency for Science, Technology and Research of (A*STAR), the US National Institute of Health (NIH) and the Susan G. Komen Breast Cancer Foundation. The SBCS was supported by Yorkshire Cancer Research S295, S299, S305PA. SEARCH is funded by a programme grant from Cancer Research UK (C490/A10124) and supported by the UK National Institute for Health Research Biomedical Research Centre at the University of Cambridge. The SZBCS was supported by Grant PBZ_KBN_122/P05/2004. SKKDKFZS is supported by the DKFZ. The TNBCC was supported by: a Specialized Program of Research Excellence (SPORE) in Breast Cancer (CA116201), a grant from the Breast Cancer Research Foundation, a generous gift from the David F. and Margaret T. Grohne Family Foundation and the Ting Tsung and Wei Fong Chao Foundation, the Stefanie Spielman Breast Cancer fund and the OSU Comprehensive Cancer Center, DBBR (a CCSG Share Resource by National Institutes of Health Grant P30 CA016056), the Hellenic Cooperative Oncology Group research grant (HR R_BG/04) and the Greek General Secretary for Research and Technology (GSRT) Program, Research Excellence II, the European Union (European Social Fund—ESF), and Greek national funds through the Operational Program ‘Education and Lifelong Learning’ of the National Strategic Reference Framework (NSRF)—ARISTEIA. The UKBGS is funded by Breakthrough Breast Cancer and the Institute of Cancer Research (ICR), London. ICR acknowledges NHS funding to the NIHR Biomedical Research Centre. The ACP study is funded by the Breast Cancer Research Trust, UK. The HERPACC was supported by a Grant-in-Aid for Scientific Research on Priority Areas from the Ministry of Education, Science, Sports, Culture and Technology of Japan, by a Grant-in-Aid for the Third Term Comprehensive 10-Year Strategy for Cancer Control from Ministry Health, Labour and Welfare of Japan, by Health and Labour Sciences Research Grants for Research on Applying Health Technology from Ministry Health, Labour and Welfare of Japan and by National Cancer Center Research and Development Fund. LAABC is supported by grants (1RB-0287, 3PB-0102, 5PB-0018 and 10PB-0098) from the California Breast Cancer Research Program. Incident breast cancer cases were collected by the USC Cancer Surveillance Program (CSP) which is supported under subcontract by the California Department of Health. The CSP is also part of the National Cancer Institute's Division of Cancer Prevention and Control Surveillance, Epidemiology, and End Results Program, under contract number N01CN25403. MYBRCA is funded by research grants from the Malaysian Ministry of Science, Technology and Innovation (MOSTI), Malaysian Ministry of Higher Education (UM.C/HlR/MOHE/06) and Cancer Research Initiatives Foundation (CARIF). Additional controls were recruited by the Singapore Eye Research Institute, which was supported by a grant from the Biomedical Research Council (BMRC08/1/35/19/550), Singapore and the National Medical Research Council, Singapore (NMRC/CG/SERI/2010). The SBCGS was supported primarily by NIH grants R01CA64277, R01CA148667 and R37CA70867. Biological sample preparation was conducted by the Survey and Biospecimen Shared Resource, which is supported by P30 CA68485. The scientific development and funding of this project were, in part, supported by the Genetic Associations and Mechanisms in Oncology (GAME-ON) Network U19 CA148065. SEBCS was supported by the BRL (Basic Research Laboratory) program through the National Research Foundation of Korea funded by the Ministry of Education, Science and Technology (2012-0000347). SGBCC is funded by the National Medical Research Council start-up Grant and Centre Grant (NMRC/CG/NCIS/2010). Additional controls were recruited by the Singapore Consortium of Cohort Studies-Multi-ethnic cohort (SCCS-MEC), which was funded by the Biomedical Research Council, grant number: 05/1/21/19/425. The TBCS was funded by The National Cancer Institute, Thailand. The TWBCS is supported by the Taiwan Biobank project of the Institute of Biomedical Sciences, Academia Sinica, Taiwan. The NBHS was supported by NIH grant R01CA100374. Biological sample preparation was conducted by the Survey and Biospecimen Shared Resource, which is supported by P30 CA68485. The SCCS is supported by a grant from the National Institutes of Health (R01 CA092447). The Arkansas Central Cancer Registry is fully funded by a grant from National Program of Cancer Registries, Centers for Disease Control and Prevention (CDC). Funding to pay the Open Access publication charges for this article was provided by the Charity Open Access Fund (COAF).

## Supplementary Material

Supplementary Data

## References

[1] Ferlay J. Shin H.R. Bray F. Forman D. Mathers C. Parkin D.M. (2010) Estimates of worldwide burden of cancer in 2008: GLOBOCAN 2008. Int. J. Cancer, 127, 2893–2917. 21351269 10.1002/ijc.25516

[2] Pharoah P.D. Antoniou A. Bobrow M. Zimmern R.L. Easton D.F. Ponder B.A. (2002) Polygenic susceptibility to breast cancer and implications for prevention. Nat. Genet., 31, 33–36. 11984562 10.1038/ng853

[3] Meijers-Heijboer H. van den Ouweland A. Klijn J. Wasielewski M. de Snoo A. Oldenburg R. Hollestelle A. Houben M. Crepin E. van Veghel-Plandsoen M. (2002) Low-penetrance susceptibility to breast cancer due to CHEK2(*)1100delC in noncarriers of BRCA1 or BRCA2 mutations. Nat. Genet., 31, 55–59. 11967536 10.1038/ng879

[4] Rahman N. Seal S. Thompson D. Kelly P. Renwick A. Elliott A. Reid S. Spanova K. Barfoot R. Chagtai T. (2007) PALB2, which encodes a BRCA2-interacting protein, is a breast cancer susceptibility gene. Nat. Genet., 39, 165–167. 17200668 10.1038/ng1959 PMC2871593

[5] Renwick A. Thompson D. Seal S. Kelly P. Chagtai T. Ahmed M. North B. Jayatilake H. Barfoot R. Spanova K. (2006) ATM mutations that cause ataxia-telangiectasia are breast cancer susceptibility alleles. Nat. Genet., 38, 873–875. 16832357 10.1038/ng1837

[6] Easton D.F. Pooley K.A. Dunning A.M. Pharoah P.D. Thompson D. Ballinger D.G. Struewing J.P. Morrison J. Field H. Luben R. (2007) Genome-wide association study identifies novel breast cancer susceptibility loci. Nature, 447, 1087–1093. 17529967 10.1038/nature05887 PMC2714974

[7] Fletcher O. Johnson N. Orr N. Hosking F.J. Gibson L.J. Walker K. Zelenika D. Gut I. Heath S. Palles C. (2011) Novel breast cancer susceptibility locus at 9q31.2: results of a genome-wide association study. J. Natl. Cancer Inst., 103, 425–435. 21263130 10.1093/jnci/djq563

[8] Ghoussaini M. Fletcher O. Michailidou K. Turnbull C. Schmidt M.K. Dicks E. Dennis J. Wang Q. Humphreys M.K. Luccarini C. (2012) Genome-wide association analysis identifies three new breast cancer susceptibility loci. Nat. Genet., 44, 312–318. 22267197 10.1038/ng.1049 PMC3653403

[9] Michailidou K. Hall P. Gonzalez-Neira A. Ghoussaini M. Dennis J. Milne R.L. Schmidt M.K. Chang-Claude J. Bojesen S.E. Bolla M.K. (2013) Large-scale genotyping identifies 41 new loci associated with breast cancer risk. Nat. Genet., 45, 353–361. 23535729 10.1038/ng.2563 PMC3771688

[10] Stacey S.N. Manolescu A. Sulem P. Rafnar T. Gudmundsson J. Gudjonsson S.A. Masson G. Jakobsdottir M. Thorlacius S. Helgason A. (2007) Common variants on chromosomes 2q35 and 16q12 confer susceptibility to estrogen receptor-positive breast cancer. Nat. Genet., 39, 865–869. 17529974 10.1038/ng2064

[11] Stacey S.N. Manolescu A. Sulem P. Thorlacius S. Gudjonsson S.A. Jonsson G.F. Jakobsdottir M. Bergthorsson J.T. Gudmundsson J. Aben K.K. (2008) Common variants on chromosome 5p12 confer susceptibility to estrogen receptor-positive breast cancer. Nat. Genet., 40, 703–706. 18438407 10.1038/ng.131

[12] Thomas G. Jacobs K.B. Kraft P. Yeager M. Wacholder S. Cox D.G. Hankinson S.E. Hutchinson A. Wang Z. Yu K. (2009) A multistage genome-wide association study in breast cancer identifies two new risk alleles at 1p11.2 and 14q24.1 (RAD51L1). Nat. Genet., 41, 579–584. 19330030 10.1038/ng.353 PMC2928646

[13] Turnbull C. Ahmed S. Morrison J. Pernet D. Renwick A. Maranian M. Seal S. Ghoussaini M. Hines S. Healey C.S. (2010) Genome-wide association study identifies five new breast cancer susceptibility loci. Nat. Genet., 42, 504–507. 20453838 10.1038/ng.586 PMC3632836

[14] Zheng W. Long J. Gao Y.T. Li C. Zheng Y. Xiang Y.B. Wen W. Levy S. Deming S.L. Haines J.L. (2009) Genome-wide association study identifies a new breast cancer susceptibility locus at 6q25.1. Nat. Genet., 41, 324–328. 19219042 10.1038/ng.318 PMC2754845

[15] Bojesen S.E. Pooley K.A. Johnatty S.E. Beesley J. Michailidou K. Tyrer J.P. Edwards S.L. Pickett H.A. Shen H.C. Smart C.E. (2013) Multiple independent variants at the TERT locus are associated with telomere length and risks of breast and ovarian cancer. Nat. Genet., 45, 371–384. 23535731 10.1038/ng.2566 PMC3670748

[16] French J.D. Ghoussaini M. Edwards S.L. Meyer K.B. Michailidou K. Ahmed S. Khan S. Maranian M.J. O'Reilly M. Hillman K.M. (2013) Functional variants at the 11q13 Risk Locus for breast cancer regulate cyclin D1 expression through long-range enhancers. Am. J. Hum. Genet., 92, 489–503. 23540573 10.1016/j.ajhg.2013.01.002 PMC3617380

[17] Garcia-Closas M. Couch F.J. Lindstrom S. Michailidou K. Schmidt M.K. Brook M.N. Orr N. Rhie S.K. Riboli E. Feigelson H.S. (2013) Genome-wide association studies identify four ER negative-specific breast cancer risk loci. Nat. Genet., 45, 392–398. 23535733 10.1038/ng.2561 PMC3771695

[18] Meyer K.B. O'Reilly M. Michailidou K. Carlebur S. Edwards S.L. French J.D. Prathalingham R. Dennis J. Bolla M.K. Wang Q. (2013) Fine-scale mapping of the FGFR2 breast cancer risk locus: putative functional variants differentially bind FOXA1 and E2F1. Am. J. Hum. Genet., 93, 1046–1060. 24290378 10.1016/j.ajhg.2013.10.026 PMC3852923

[19] Sawyer E. Roylance R. Petridis C. Brook M.N. Nowinski S. Papouli E. Fletcher O. Pinder S. Hanby A. Kohut K. (2014) Genetic predisposition to in situ and invasive lobular carcinoma of the breast. PLoS Genet., 10, e1004285. 24743323 10.1371/journal.pgen.1004285 PMC3990493

[20] Burton H. Chowdhury S. Dent T. Hall A. Pashayan N. Pharoah P. (2013) Public health implications from COGS and potential for risk stratification and screening. Nat. Genet., 45, 349–351. 23535723 10.1038/ng.2582

[21] Warren H. Dudbridge F. Fletcher O. Orr N. Johnson N. Hopper J.L. Apicella C. Southey M.C. Mahmoodi M. Schmidt M.K. (2012) 9q31.2-rs865686 as a susceptibility locus for estrogen receptor-positive breast cancer: evidence from the Breast Cancer Association Consortium. Cancer Epidemiol. Biomarkers Prev., 21, 1783–1791. 22859399 10.1158/1055-9965.EPI-12-0526 PMC3772723

[22] Howie B.N. Donnelly P. Marchini J. (2009) A flexible and accurate genotype imputation method for the next generation of genome-wide association studies. PLoS Genet., 5, e1000529. 19543373 10.1371/journal.pgen.1000529 PMC2689936

[23] Edwards S.L. Beesley J. French J.D. Dunning A.M. (2013) Beyond GWASs: illuminating the dark road from association to function. Am. J. Hum. Genet., 93, 779–797. 24210251 10.1016/j.ajhg.2013.10.012 PMC3824120

[24] Encode Project Consortium Bernstein B.E. Birney E. Dunham I. Green E.D. Gunter C. Snyder M. (2012) An integrated encyclopedia of DNA elements in the human genome. Nature, 489, 57–74. 22955616 10.1038/nature11247 PMC3439153

[25] Frietze S. Wang R. Yao L. Tak Y.G. Ye Z. Gaddis M. Witt H. Farnham P.J. Jin V.X. (2012) Cell type-specific binding patterns reveal that TCF7L2 can be tethered to the genome by association with GATA3. Genome Biol., 13, R52. 22951069 10.1186/gb-2012-13-9-r52 PMC3491396

[26] Ernst J. Kheradpour P. Mikkelsen T.S. Shoresh N. Ward L.D. Epstein C.B. Zhang X. Wang L. Issner R. Coyne M. (2011) Mapping and analysis of chromatin state dynamics in nine human cell types. Nature, 473, 43–49. 21441907 10.1038/nature09906 PMC3088773

[27] Kittler R. Zhou J. Hua S. Ma L. Liu Y. Pendleton E. Cheng C. Gerstein M. White K.P. (2013) A comprehensive nuclear receptor network for breast cancer cells. Cell Rep., 3, 538–551. 23375374 10.1016/j.celrep.2013.01.004

[28] Kouros-Mehr H. Slorach E.M. Sternlicht M.D. Werb Z. (2006) GATA-3 maintains the differentiation of the luminal cell fate in the mammary gland. Cell, 127, 1041–1055. 17129787 10.1016/j.cell.2006.09.048 PMC2646406

[29] Sanders D.A. Ross-Innes C.S. Beraldi D. Carroll J.S. Balasubramanian S. (2013) Genome-wide mapping of FOXM1 binding reveals co-binding with estrogen receptor alpha in breast cancer cells. Genome Biol., 14, R6. 23347430 10.1186/gb-2013-14-1-r6 PMC3663086

[30] Boyle A.P. Hong E.L. Hariharan M. Cheng Y. Schaub M.A. Kasowski M. Karczewski K.J. Park J. Hitz B.C. Weng S. (2012) Annotation of functional variation in personal genomes using RegulomeDB. Genome Res., 22, 1790–1797. 22955989 10.1101/gr.137323.112 PMC3431494

[31] Hurtado A. Holmes K.A. Ross-Innes C.S. Schmidt D. Carroll J.S. (2011) FOXA1 is a key determinant of estrogen receptor function and endocrine response. Nat. Genet., 43, 27–33. 21151129 10.1038/ng.730 PMC3024537

[32] Li Q. Seo J.H. Stranger B. McKenna A. Pe'er I. Laframboise T. Brown M. Tyekucheva S. Freedman M.L. (2013) Integrative eQTL-based analyses reveal the biology of breast cancer risk loci. Cell, 152, 633–641. 23374354 10.1016/j.cell.2012.12.034 PMC4165609

[33] Mavaddat N. Antoniou A.C. Easton D.F. Garcia-Closas M. (2010) Genetic susceptibility to breast cancer. Mol. Oncol., 4, 174–191. 20542480 10.1016/j.molonc.2010.04.011 PMC5527934

[34] Haiman C.A. Chen G.K. Vachon C.M. Canzian F. Dunning A. Millikan R.C. Wang X. Ademuyiwa F. Ahmed S. Ambrosone C.B. (2011) A common variant at the TERT-CLPTM1L locus is associated with estrogen receptor-negative breast cancer. Nat. Genet., 43, 1210–1214. 22037553 10.1038/ng.985 PMC3279120

[35] Siddiq A. Couch F.J. Chen G.K. Lindstrom S. Eccles D. Millikan R.C. Michailidou K. Stram D.O. Beckmann L. Rhie S.K. (2012) A meta-analysis of genome-wide association studies of breast cancer identifies two novel susceptibility loci at 6q14 and 20q11. Hum. Mol. Genet., 21, 5373–5384. 22976474 10.1093/hmg/dds381 PMC3510753

[36] NCI-NHGRI Working Group on Replication in Association Studies Chanock S.J. Manolio T. Boehnke M. Boerwinkle E. Hunter D.J. Thomas G. Hirschhorn J.N. Abecasis G. Altshuler D. (2007) Replicating genotype-phenotype associations. Nature, 447, 655–660. 17554299 10.1038/447655a

[37] Carroll J.S. Liu X.S. Brodsky A.S. Li W. Meyer C.A. Szary A.J. Eeckhoute J. Shao W. Hestermann E.V. Geistlinger T.R. (2005) Chromosome-wide mapping of estrogen receptor binding reveals long-range regulation requiring the forkhead protein FoxA1. Cell, 122, 33–43. 16009131 10.1016/j.cell.2005.05.008

[38] Eeckhoute J. Keeton E.K. Lupien M. Krum S.A. Carroll J.S. Brown M. (2007) Positive cross-regulatory loop ties GATA-3 to estrogen receptor alpha expression in breast cancer. Cancer Res., 67, 6477–6483. 17616709 10.1158/0008-5472.CAN-07-0746

[39] Theodorou V. Stark R. Menon S. Carroll J.S. (2013) GATA3 acts upstream of FOXA1 in mediating ESR1 binding by shaping enhancer accessibility. Genome Res., 23, 12–22. 23172872 10.1101/gr.139469.112 PMC3530671

[40] Cowper-Sal lari R. Zhang X. Wright J.B. Bailey S.D. Cole M.D. Eeckhoute J. Moore J.H. Lupien M. (2012) Breast cancer risk-associated SNPs modulate the affinity of chromatin for FOXA1 and alter gene expression. Nat. Genet., 44, 1191–1198. 23001124 10.1038/ng.2416 PMC3483423

[41] Li Q. Stram A. Chen C. Kar S. Gayther S. Pharoah P. Haiman C. Stranger B. Kraft P. Freedman M.L. (2014) Expression QTL-based analyses reveal candidate causal genes and loci across five tumor types. Hum. Mol. Genet., 23, 5294–5302. 24907074 10.1093/hmg/ddu228 PMC4215106

[42] Rowland B.D. Bernards R. Peeper D.S. (2005) The KLF4 tumour suppressor is a transcriptional repressor of p53 that acts as a context-dependent oncogene. Nat. Cell Biol., 7, 1074–1082. 16244670 10.1038/ncb1314

[43] Foster K.W. Frost A.R. McKie-Bell P. Lin C.Y. Engler J.A. Grizzle W.E. Ruppert J.M. (2000) Increase of GKLF messenger RNA and protein expression during progression of breast cancer. Cancer Res., 60, 6488–6495. 11103818

[44] Ernst J. Kellis M. (2010) Discovery and characterization of chromatin states for systematic annotation of the human genome. Nat. Biotechnol., 28, 817–825. 20657582 10.1038/nbt.1662 PMC2919626

[45] van Berkum N.L. Lieberman-Aiden E. Williams L. Imakaev M. Gnirke A. Mirny L.A. Dekker J. Lander E.S. (2010) Hi-C: a method to study the three-dimensional architecture of genomes. J. Vis. Exp., 39. 10.3791/1869 PMC3149993 20461051

[46] Miele A. Gheldof N. Tabuchi T.M. Dostie J. Dekker J. (2006) Mapping chromatin interactions by chromosome conformation capture. Curr. Protoc. Mol. Biol., Chapter 21, Unit 21.11. 10.1002/0471142727.mb2111s74 18265379

[47] Broeks A. Schmidt M.K. Sherman M.E. Couch F.J. Hopper J.L. Dite G.S. Apicella C. Smith L.D. Hammet F. Southey M.C. (2011) Low penetrance breast cancer susceptibility loci are associated with specific breast tumor subtypes: findings from the Breast Cancer Association Consortium. Hum. Mol. Genet., 20, 3289–3303. 21596841 10.1093/hmg/ddr228 PMC3140824

