# Supplementary material for: Fine-mapping identifies two additional breast cancer susceptibility loci at 9q31.2
Source: Hum Mol Genet. 2015 Feb 4;24(10):2966–84. doi: 10.1093/hmg/ddv035 (PMC4406292; doi:10.1093/hmg/ddv035)
Supplement: Supplementary Data [file supp_24_10_2966__index.html]

Fine-mapping identifies two additional breast cancer susceptibility loci at 9q31.2 — Fine-mapping identifies two additional breast cancer susceptibility loci at 9q31.2 — Supplementary Data 

# Fine-mapping identifies two additional breast cancer susceptibility loci at 9q31.2

## Supplementary Data

Supplementary Data

**Files in this Data Supplement:**

- Supplementary Data - Docx file
- Supplementary Tables - doc file
