# Supplementary figures and images for: Fine-mapping identifies two additional breast cancer susceptibility loci at 9q31.2
Source: Hum Mol Genet. 2015 Feb 4;24(10):2966–84. doi: 10.1093/hmg/ddv035 (PMC4406292; doi:10.1093/hmg/ddv035)

**Supplemental Figure 1. Genotype cluster plots for SNPs rs10816625, rs13294895 and rs676256.**


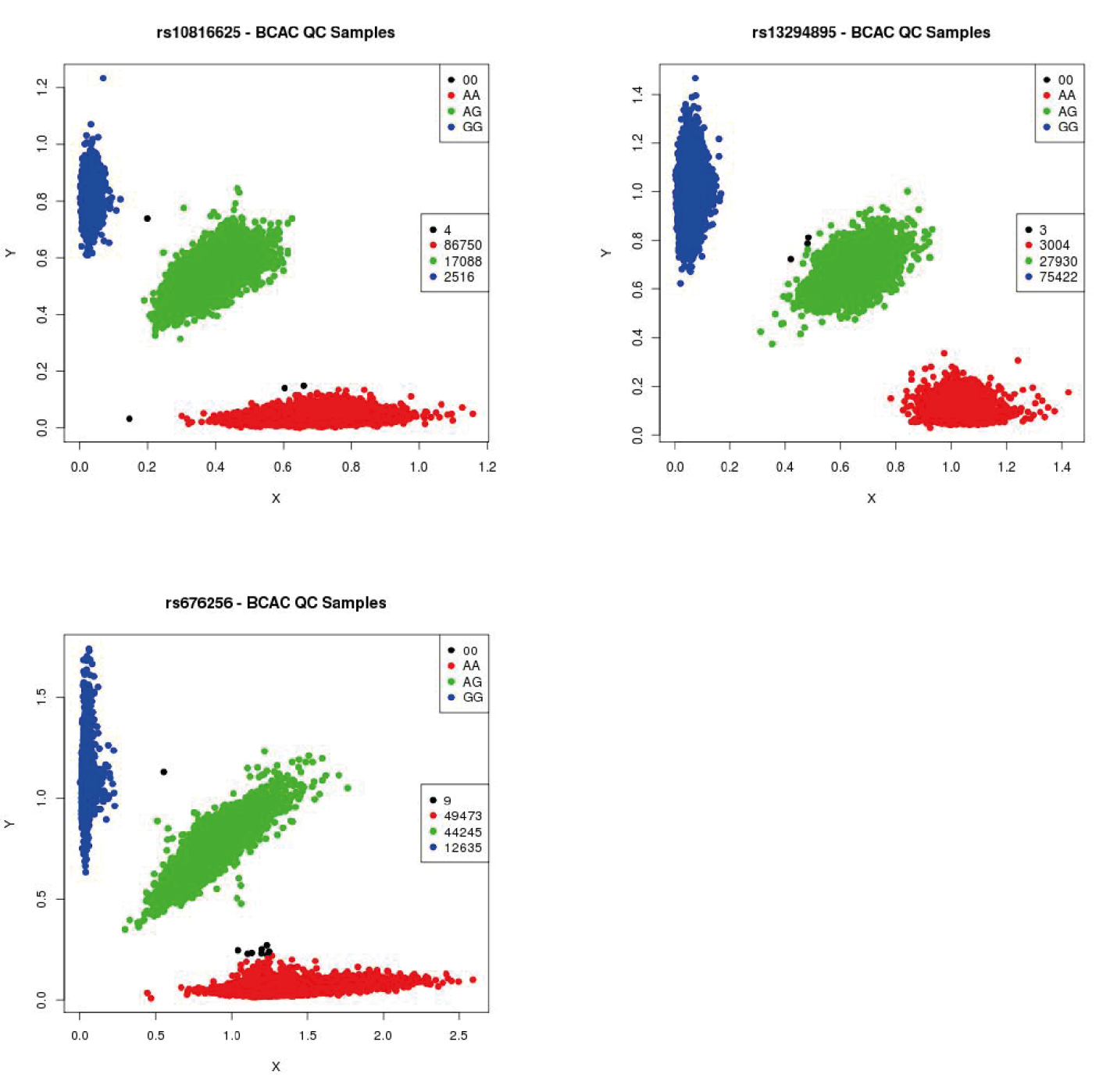

Supplement: Supplementary Data [file supp_ddv035_ddv035supp.docx]
