# Supplementary material for: Fine-mapping identifies two additional breast cancer susceptibility loci at 9q31.2
Source: Hum Mol Genet. 2015 Feb 4;24(10):2966–84. doi: 10.1093/hmg/ddv035 (PMC4406292; doi:10.1093/hmg/ddv035)
Supplement: Supplementary Data [file supp_ddv035_ddv035supp_tables.doc]

**Supplemental Table 1. Characteristics of individual contributing studies.**

| **Study Abbreviation** | **Study Name** | **Country** | **Case definition** | **Control definition** | **Number Cases** | **Number Controls** |
| --- | --- | --- | --- | --- | --- | --- |
| ABCFS | Australian Breast Cancer Family Study | Australia | All cases diagnosed < age 40 plus a random sample of those diagnosed ages 40-59 from cancer registries in Victoria and New South Wales, plus a limited number diagnosed aged 60-69; cases living in Melbourne recruited from 1992-99 and in Sydney from 1993-98. | Controls identified from the electoral rolls in Melbourne from 1992-98 and Sydney from 1993-99. Frequency matched to cases by age in 5 year categories. | 790 | 551 |
| ABCS | Amsterdam Breast Cancer Study | Netherlands | All cases (operable, invasive breast cancer) diagnosed between 1974-1994 in four Dutch hospitals. Familial non-BRCA1/2 cases <50 from Clinical Genetic Centre in the Netherlands Cancer Institute | Population-based female bloodbank controls of all ages. | 1,256 | 1,429 |
| ACP | Asia Cancer Program | Thailand | Cases < 71 years of age recruited between 1999-2000 and 2008-present at The National Cancer Institute (Central region); The Prince Songkla University Research Centre (South region); The HRH Princess Maha Chakri Sirindhorn Medical Centre (MSMC)-Srinakarinviroj University (Eastern region); and Khon-Kaen University Cancer Centre (North-eastern region). | Controls < 71 years of age recruited between 1999-2000 and 2008-present at The National Cancer Institute (Central region); The Prince Songkla University Research Centre (South region); The HRH Princess Maha Chakri Sirindhorn Medical Centre (MSMC)-Srinakarinviroj University (Eastern region); and Khon-Kaen University Cancer Centre (North-eastern region). | 418 | 636 |
| BBCC | Bavarian Breast Cancer Cases and Controls | Germany | Consecutive, unselected cases with invasive breast cancer recruited at the University Breast Centre, Franconia in Northern Bavaria during 2002-2010. | Healthy women with no diagnosis of cancer aged 55 or older. Invited by a newspaper advertisement in Northern Bavaria, and recruited during 2002-2010. | 554 | 458 |
| BBCS | British Breast Cancer Study | UK | Cases from English & Scottish Cancer Registries: all breast cancer cases who developed a first primary before age 65 in 1971 or later and who subsequently developed a second primary cancer; or unilateral breast cancer cases diagnosed before age 70 in 1971 or later. | A friend, sister-in-law, daughter-in-law or other non-blood relative of cases. Recruitment of cases and controls began in January 2001. | 1,446 | 1,397 |
| BIGGS | Breast Cancer in Galway Genetic Study | Ireland | Unselected cases recruited from West of Ireland since 2001. Cases were recruited from University College Hospital Galway and surrounding hospitals | Women > 60 years with no personal history of any cancer and no family History of breast or ovarian cancer were identified from retirement groups in the West of Ireland (same catchment area as cases) during the period 2001-2008. | 795 | 719 |
| BSUCH | Breast Cancer Study of the University of Heidelberg | Germany | All cases diagnosed with breast cancer in 2007-2009 at the University Women`s Clinic Heidelberg | Healthy, unrelated, ethnically matched female blood donors recruited in 2007 & 2009 by German Red Cross Blood Service of Baden-Württemberg-Hessen, Institute of Transfusion Medicine & Immunology, Mannheim. | 815 | 954 |
| CECILE | CECILE Breast Cancer Study | France | All cases diagnosed with breast cancer in 2005-2007 among women <75 years of age and residing in Ille-et-Vilaine (Rennes) or in Côte d'Or (Dijon) at diagnosis. Cases were recruited from the main cancer treatment center (Centre Eugène-Marquis in Rennes and Centre Georges-François-Leclerc in Dijon) and from private or public hospitals in each area. | General population control women residing in the same areas. Controls were recruited in 2005-2007 using a random digit dialling procedure and quotas by socioeconomic status, and were frequency-matched to the cases by 5-year age groups. | 900 | 999 |
| CGPS | Copenhagen General Population Study | Denmark | Consecutive, incident cases from 1 hospital with centralized care for a population of 400,000 women from 2001 to the present. | Community controls residing in the same region as cases and with no history of breast cancer were identified from the Copenhagen General Population Study recruited 2003-2007. All controls were known to still be breast cancer-free at the end of 2007. | 2,811 | 4,086 |
| CNIO-BCS | Spanish National Cancer Centre Breast Cancer Study | Spain | Two groups of cases: 1) 574 consecutive breast cancer patients, unselected for family history, from 3 public hospitals, 2 in Madrid and one in Oviedo, from 2000 to 2005. 2) 291 cases with at least one first degree relative also affected with breast cancer, recruited through the CNIO family cancer clinic in Madrid from 2000 to 2004. | Women attending the Menopause Research Centre between 2000 and 2004 and female members of the College of Lawyers attending a free, targeted medical check-up in 2005, all free of breast cancer and all in Madrid | 867 | 876 |
| CTS | California Teachers Study | USA | This is a nested case-control study conducted within a cohort of California teachers (113,590) who were under age 80 years at baseline, had no prior history of invasive or in situ breast cancer. Cases are women newly diagnosed with a histologically confirmed invasive primary adenocarcinoma of the breast at age 80 years or younger from 1998 to 2008. | Controls are a probability sample of at-risk cohort members, frequency matched to cases on age at baseline (5-year age groups), self-reported race/ethnicity (white, African American, Latina, Asian, other), and broad geographic region within California. Controls were recruited during 1998 to 2008 and selected without replacement, using an assigned reference date. | 68 | 71 |
| DEMOKRITOS | Demokritos | Greece | Triple negative breast cancer cases enrolled from 1997-2010 in hospitals serving geographical areas of Greece, including Athens metropolitan area, Thessaloniki, Ioannina, Patras, and Crete (Chania), in collaboration with the Hellenic Cooperative Oncology Group (HECOG). | Regional controls, identified between 2010-2011 from Athens and Thessaloniki, were population-based unaffected women of the same age range. | 413 | 95 |
| ESTHER | ESTHER Breast Cancer Study | Germany | Statewide recruitment of breast cancer cases in all hospitals in Saarland/Germany between 1996-1998 and in 2001-2003. | Statewide recruitment of participants of a routine health check-up in Saarland/Germany in 2000-2002. A stratified random sample, matched to the cases by five-year age groups, was selected as controls. | 471 | 502 |
| GENICA | Gene Environment Interaction and Breast Cancer in Germany | Germany | Incident breast cancer cases enrolled between 2000 and 2004 from the Greater Bonn area (by of the hospitals within the study region); all enrolled within 6 months of diagnosis | Selected from population registries from 31 communities in the greater Bonn area; matched to cases in 5-year age classes between 2001 and 2004 | 465 | 427 |
| HEBCS | Helsinki Breast Cancer Study | Finland | (1) Consecutive cases (883) from the Department of Oncology, Helsinki University Central Hospital 1997-8 and 2000, (2) Consecutive cases (986) from the Department of Surgery, Helsinki University Central Hospital 2001 – 2004, (3) Familial breast cancer patients (536) from the Helsinki University Central Hospital, Departments of Oncology and Clinical Genetics (1995-) | Healthy females from the same geographical region in Southern Finland in 2003. | 1,517 | 1,234 |
| HERPACC | Hospital-based Epidemiologic Research Program at Aichi Cancer Center | Japan | Incident breast cancer cases that firstly visited Aichi Cancer Center between 2001 and 2005 and were diagnosed within 1 year from the first visit. No previous history of any type of cancer. | Controls were selected from pool of non-cancer patients who firstly visited Aichi Cancer Center between 2001-2005. Non-cancer status is defined as "having no positive finding on any of clinical/laboratory/graphical examination within 1 year from their fist visit. No previous history of cancer is allowed. | 560 | 1,376 |
| HMBCS | Hannover-Minsk Breast Cancer Study | Belarus | Ascertainment at the Byelorussian Institute for Oncology and Medical Radiology Aleksandrov N.N. in Minsk or at one of 5 regional oncology centers in Gomel, Mogilev, Grodno, Brest or Vitebsk through the years 2002-2008. | Controls from the same population aged 18-72 years. Healthy (without personal history of cancer) female probands recruited from the same geographical regions as cases during the years 2002-2008. About 75% of controls were women invited for general medical examination at five regional gynaecology clinics (in Gomel, Mogilev, Grodno, Brest or Vitebsk) and cancer-free volunteers ascertained at the Institute for Inherited Diseases in Minsk; 20% were cancer-free female blood bank donors recruited at Republic Blood Bank, Minsk, Belarus; finally 5% of controls were healthy cancer-free relatives of some breast cancer patients. | 688 | 130 |
| KARBAC | Karolinska Breast Cancer Study | Sweden | 1. Familial cases from Department of Clinical Genetics, Karolinska University Hospital, Stockholm. 2. Consecutive cases from Department of Oncology, Huddinge & Söder Hospital, Stockholm 1998-2000 | Blood donors of mixed gender from same geographical region. Excess material was received from all blood donors over a 3 month period in 2004 (approximately 3000) and DNA was extracted from a random sample of 1500 | 722 | 662 |
| KBCP | Kuopio Breast Cancer Project | Finland | Women seen at Kuopio University Hospital between 1990 and 1995 because of breast lump, mammographic abnormality, or other breast symptom who were found to have breast cancer | Age and long-term area-of-residence matched controls selected from the National Population Register and interviewed in parallel with the cases | 411 | 251 |
| kConFab/AOCS | Kathleen Cuningham Foundation Consortium for research into Familial Breast Cancer/Australian Ovarian Cancer Study | Australia and New Zealand | Cases were from multiple-case breast and breast-ovarian families recruited though family cancer clinics from across Australia and New Zealand from 1998 to the present. Cases were selected for inclusion in BCAC studies if (i) family was negative for mutations in BRCA1 and BRCA2 (ii) case was the index for the family, defined as youngest breast cancer affected family member. | Female controls were ascertained by the Australian Ovarian Cancer Study, identified from the electoral rolls from all over Australia from 2002-2006. | 410 | 897 |
| LAABC | Los Angeles County Asian-American Breast Cancer Case-Control Study | USA | Incident cases recruited from 1995-2007 and identified from SEER cancer registries in Los Angeles County. Grouped by self-reported ethnicity. | Controls were recruited during 1995-2009 and selected from the same neighbourhood as where cancer cases resided at the time of diagnosis. Controls were frequency-matched to the cases on specific Asian ethnicities and 5-year age groups | 808 | 990 |
| LMBC | Leuven Multidisciplinary Breast Centre | Belgium | All patients diagnosed with breast cancer and seen in the Multidisciplinary Breast Center in Leuven (Gashuisberg) since June 2007 plus retrospective collection of cases diagnosed since 2000 | Healthy controls (blood donors) collected at the Red Cross and located in Gasthuisberg hospital (Oct-2007-March 2008) | 2,616 | 1,388 |
| MARIE | Mammary Carcinoma Risk Factor Investigation | Germany | Incident cases diagnosed from 2001-2005 in the study region Hamburg in Northern Germany, and from 2002-2005 in the study region Rhein-Neckar-Karlsruhe in Southern Germany. | 2 controls per case were randomly drawn from population registries and frequency matched by birth year and study region to the case. Controls were recruited from 2002 to 2006. | 1,656 | 1,778 |
| MBCSG | Milan Breast Cancer Study Group | Italy | Familial and/or early onset breast cancer patients (aged 22-87) negative for mutations in BRCA genes, ascertained in two large cancer centres in Milan from 2000 to date. | Healthy blood donors aged 18-71 years, recruited at two blood centres in Milan from 2004 (centre 1) and 2007 (centre 2) to date | 189 | 400 |
| MCBCS | Mayo Clinic Breast Cancer Study | USA | Incident cases residing in 6 states (MN, WI, IA, IL, ND, SD) seen at the Mayo Clinic in Rochester, MN from 2002-5 | Women without cancer presenting for general medical examination at the Mayo Clinic. Controls were recruited concurrently with cases and were frequency matched to cases on age, ethnicity and county/state | 1,546 | 1,931 |
| MCCS | Melbourne Collaborative Cohort Study | Australia | Incident cases diagnosed within the Melbourne Collaborative Cohort Study during the follow-up from baseline (1990-1994) to 2008 of the 24469 participating women | Random sample of the initial cohort | 614 | 511 |
| MEC | Multi-ethnic Cohort | USA | Incident cases identified from SEER cancer registries in Los Angeles County & State registries in California & Hawaii, USA from 1993-2002. Grouped by self-reported ethnicity. | Women without cancer from the same States, recruited concurrently with cases & frequency matched to cases by age at blood-draw & self-reported ethnicity. | 705 | 741 |
| MTLGEBCS | Montreal Gene-Environment Breast Cancer Study | Canada | All cases are postmenopausal women (47-75 years) living in Montreal with a primary invasive breast cancer and with no previous occurrence of any type of cancer. All cases were identified from 2007 to 2010 in 15 of 16 Montreal hospitals that treat breast cancer. | Random sample from the universal Provincial Voter Registration List, approximately frequency-matched to cases on age (5-year bins) and living in Montreal. | 489 | 436 |
| MYBRCA | Malaysian Breast Cancer Genetic Study | Malaysia | Breast cancer cases identified at the Breast Cancer Clinic in University Malaya Medical Centre Jan 2001-July 2010; cases are a mixture of prevalent and incident cases | Controls are cancer-free individuals (21-70 years) with no breast or ovarian cancer in first-degree relatives, randomly selected from women attending same hospital. | 770 | 610 |
| NBCS | Norwegian Breast Cancer Study | Norway | Incidence cases from three different hospitals: 1) Cases (114) mean age 64 (28-92) at Ullevål Univ. Hospital 1990-94, 2) cases (182) mean age 59 (26-75) referred to Norwegian Radium Hospital 1975-1986, 3) cases (124), mean age 56 (29-82) ) with stage I or II disease, in the Oslo micro-metastases study at Norwegian Radium Hospital between 1995-1998, 4) cases (71) mean age 67 (37–82) with locally advanced disease at Haukeland Univ. Hospital. | Control subjects were healthy women, age 55-71, residing in Tromsø (440), and Bergen (109) attending the Norwegian Breast Cancer Screening Program. | 22 | 70 |
| NBHS | Nashville Breast Health Study | USA | Through a rapid case-ascertainment system, we identified newly-diagnosed breast cancer cases through the Tennessee State Cancer Registry and five major hospitals in the city that provide medical care for breast cancer patients. Eligible cases were women diagnosed with invasive breast cancer or ductal carcinoma in situ, who were between the ages of 25 and 75, had no prior history of cancer other than non-melanoma skin cancer, had a resident telephone, spoke English, and who were able to provide consent to the study. Recruitment period was from 2001 to 2011. The recruitment for European Americans ended in 2008. | Controls were identified via random digit dialing (RDD) of households in the same geographic area as cases during 2001-2011. Eligibility criteria for controls were the same as cases with the exception that controls did not have a prior cancer diagnosis other than simple skin cancer. Controls were frequency matched to cases on 5-year age group, race, and county of residence. | 492 | 370 |
| OBCS | Oulu Breast Cancer Study | Finland | Consecutive incident cases diagnosed at the Oulu University Hospital between 2000 and 2004. | Healthy, consecutive, anonymous, female Finnish Red-Cross blood donors recruited in 2002 from the same geographical region in Northern Finland. | 500 | 414 |
| OFBCR | Ontario Familial Breast Cancer Registry | Canada | Cases diagnosed between 1 Jan 1996-31 Dec 1998 were identified from the Ontario Cancer Registry which registers >97% of all cases residing in the province at the time of diagnosis. All women with invasive breast cancer aged 20–54 years who met the OFBCR definition for high genetic risk (family history of specific cancers particularly breast and ovarian, early onset disease, Ashkenazi ethnicity or a diagnosis of multiple breast cancer) were asked to participate by completing risk factor questionnaires and providing a blood sample. A 25% random sample of individuals in this age category who did not meet the OFBCR definition, 35% of those aged 55–69 at high risk and 8.75% aged 55–69 at low risk were also asked to participate. This multi-step sampling scheme enriched the population for genetically predisposed individuals, which was an objective of the Ontario Familial Breast Cancer Registry. | Unrelated, unaffected population controls were recruited between 2003-2005 by calling randomly selected residential telephone numbers throughout the same geographical region. Eligible controls were women with no history of breast cancer and were frequency-matched by 5-year age group to the expected age distribution of cases. Approximately, 65% of identified eligible women returned questionnaires, and 63% of these donated a blood specimen. | 1,156 | 511 |
| ORIGO | Leiden University Medical Centre Breast Cancer Study | Netherlands | Consecutive cases diagnosed 1996-2006 in two hospitals of South-West Netherlands (Leiden & Rotterdam). No selection for family history; Rotterdam cases selected for diagnosis aged <70. Cases with in situ carcinomas eligible. | Three groups of controls: (1) Blood bank healthy donors from Southwest Netherlands recruited in 1996, 2000 or 2007; (2) People who married a person who was part of a family with high breast cancer risk (BRCA1/2/x). From the Southwest of the Netherlands, recruited 1990-1996; (3) Females tested at the local clinical genetics department for familial diseases, excluding familial cancer syndromes (no mutation found in gene(s) related to the disease being tested), recruited 1995-2007. | 335 | 327 |
| OSU | Ohio State University Study | USA | Incident triple negative invasive breast cancer cases from a collection of incident breast cancer cases diagnosed in Columbus, Ohio (2006-2011). | Population-matched controls accrued through primary care clinics in the OSU medical center system (2006-2011). | 207 | 203 |
| PBCS | NCI Polish Breast Cancer Study | Poland | Incident cases from 2000-2003 identified through a rapid identification system in participating hospitals covering ~ 90% of all eligible cases, and cancer registries in Warsaw and Łódź covering 100% of all eligible cases | Randomly selected from population lists of all residents of Poland, stratified and frequency matched to cases by case city and age in 5-year categories. Recruited 2000-2003. | 519 | 424 |
| pKARMA | Karolinska Mammography Project for Risk Prediction of Breast Cancer - prevalent cases | Sweden | Incident cases from Jan 2001 – Dec 2008 from the Stockholm/Gotland area. Identified through the Stockholm breast cancer registry. | Unmatched participants of the KARMA mammography screening study recruited between 2010 and 2011 from Helsingborg and Stockholm. | 4,553 | 5,537 |
| RBCS | Rotterdam Breast Cancer Study | Netherlands | Familial breast cancer patients selected from the clinical genetics center at Erasmus Medical Center; recruited 1994 - 2005 | Spouses or mutation-negative siblings of heterozygous Cystic Fibrosis mutation carriers selected from the clinical genetics center at Erasmus Medical Center; recruited 1996 - 2006 | 620 | 699 |
| RPCI | Roswell Park Cancer Institute Study | USA | Triple negative invasive breast cancer cases from incident cases recruited to the RPCI Data Bank and Biorepository from 2006-2010. | Healthy controls identified from employee volunteers, and women recruited from community events from 2006-2010. | 47 | 126 |
| SASBAC | Singapore and Sweden Breast Cancer Study | Sweden | Incident cases from October 1993 to March 1995 identified via the 6 regional cancer registries in Sweden, to which reporting is mandatory. | Controls were randomly selected from the total population registry in 5-year age groups to match the expected age-frequency distribution among cases. Patients and controls were recruited from Oct 1993 through April 1995. | 1,163 | 1,378 |
| SBCGS | Shanghai Breast Cancer Genetic Study | China | Newly diagnosed breast cancer cases recruited from 1996 -2009. Cases were identified mostly from the Shanghai Cancer Registry. Some cases were identified from the Shanghai Women’s Health Study. | Community controls randomly selected from the general population using the resident registry or from cancer-free cohort members in the Shanghai Women’s Health Study. The controls were recruited from the same geographical region as cases during 1996-2009. | 829 | 892 |
| SBCS | Sheffield Breast Cancer Study | UK | Women with pathologically confirmed breast cancer recruited from surgical outpatient clinics at the Royal Hallamshire Hospital, Sheffield, 1998 – 2005; cases are a mixture of prevalent and incident disease | Unselected women attending the Sheffield Mammography Screening Service between Sep 2000 - Aug 2004, if their mammograms showed no evidence of a breast lesion | 751 | 848 |
| SCCS | Southern Community Cohort Study | USA | Incident cases (N=222) from 2002-2009 identified from 12 state cancer registries in southeastern US. Prevalent cases (N=493) identified by self-reported history of breast cancer reported on cohort enrolment survey. | Controls for the incident cases were individually matched on age (+/- 2 years), self-reported race, menopausal status, enrolment site, and date of sample collection (+/- 6 months). Controls for the prevalent cases were frequency matched on age (+/- 1 year) and enrolment site. | 679 | 680 |
| SEARCH | Study of Epidemiology and Risk factors in Cancer Heredity | UK | 2 groups of cases identified through East Anglian Cancer Registry; 1) prevalent cases diagnosed 1991-1996 under 55 years of age at diagnosis, recruited 1996-2002; 2) incident cases diagnosed since 1996 under 70 years of age at diagnosis, recruited 1996-present. | Two groups of controls: (1) selected from the EPIC-Norfolk cohort study of 25,000 individuals age 45-74 recruited between 1992 and1994, based in the same geographic region as cases; (2) selected from GP practices from March 2003 to present, frequency matched to cases by age and geographic region | 9,097 | 8,069 |
| SEBCS | Seoul Breast Cancer Study | Korea | Consecutive, incident, cases from 2 hospitals in Seoul recruited 2001-2005 | Healthy community controls from same catchment area and participating in annual health check-up, 2001-2005. | 1,114 | 1,129 |
| SGBCC | Singapore Breast Cancer Cohort | Singapore | Living breast cancer patients diagnosed with primary *in situ* or invasive breast cancer at National University Hospital between 2006-2010. Cases are a mixture of prevalent and incident cases. | All community-dwelling individuals who are Singaporeans or Singaporean Permanent Residents, 21 years and older. Participants were recruited between 2006 and 2010 through word-of-mouth and personal recommendations. In some cases, recruiters also sought participants through "cold-calling" or through door-to-door invitations. Exclusion criteria were a medical history of cancer, acute myocardial infarction or stroke, or major psychiatric morbidity including schizophrenia, psychotic depression, and advanced Alzheimer’s Disease. | 384 | 502 |
| SKKDKFZS | Städtisches Klinikum Karlsruhe Deutsches Krebsforschungszentrum Study | Germany | Women diagnosed with primary *in situ* or invasive breast cancer at the Städtisches Klinikum Karlsruhe from March 1993 to July 2005. | Healthy, unrelated, ethnically matched female blood donors recruited in 2004 & 2007 by German Red Cross Blood Service of Baden-Württemberg-Hessen, Institute of Transfusion Medicine & Immunology, Mannheim. | 134 | 168 |
| SZBCS | IHCC-Szczecin Breast Cancer Study | Poland | Prospectively ascertained cases of invasive breast cancer patients diagnosed at the Regional Oncology Hospital (Szczecin) in the years 2002, 2003, 2006 and 2007 or the University Hospital from 2002 to 2007 in Szczecin, West-Pomerania, Poland. Patients with pure intraductal or intralobular cancer were excluded (DCIS or LCIS) but patients with DCIS with micro-invasion were included. | Unaffected, matched to cases for year of birth, sex and region; from families with negative cancer family history; controls were part of a population- based study of the 1.3 million inhabitants of West Pomerania performed in 2003 and 2004 designed to identify familial aggregations of cancer by our centre. | 303 | 315 |
| TBCS | IARC-Thai Breast Cancer Study | Thailand | Incident cases diagnosed at the National Cancer Institute (NCI) in Bangkok and Khon Kaen Hospital during the period May 2002-March 2004. | Controls were randomly selected healthy females visiting hospital patients with diseases other than breast or ovarian cancer at NCI Bangkok and Khon Kaen Hospital during the period May 2002-March 2004. | 138 | 253 |
| TWBCS | Taiwanese Breast Cancer Study | Taiwan | Incident cases diagnosed & treated at 2 major teaching hospitals in Taiwan between March 2002 and August 2005. | Controls cancer-free individuals, randomly selected from women attending health exam at same hospital during study period. Underwent 1-day health examination - any showing evidence cancer excluded. | 774 | 236 |
| UKBGS | UK Breakthrough Generations Study | UK | Cohort members who developed breast cancer or in situ breast cancer after entry into the Breakthrough Generations Study (cohort of >100,000 women followed up for breast cancer, recruited from the UK during 2003-2010). | Women who had not had breast cancer or in situ breast cancer selected by 1:1 matching to cases on date of birth, year of entry in to the study (2003-2010), source of recruitment, availability of blood sample and ethnicity. | 414 | 470 |

CTS, NBCS and SKKDKFZS are studies in BCAC but genotyped as part of the triple negative breast cancer consortium (TNBCC). Controls (n=140) for triple negative breast cancers of SKKDKFZS were selected from the German Consortium for Hereditary Breast & Ovarian Cancer.

**Supplemental Table 2A. Association statistics and effect estimates for 9q31.2 fine-mapping SNPs in breast cancer cases and controls with European ancestry.**

| SNP | Location | Alleles | MAF | Trend OR | *P*-trend | Het OR | Hom OR | *P*-geno |
| --- | --- | --- | --- | --- | --- | --- | --- | --- |
| rs2900441 | 110740582 | T,A | 0.37 | 0.98 (0.96-1) | 0.03 | 0.98 (0.95-1.01) | 0.96 (0.92-1) | 0.10 |
| rs837410 | 110740832 | G,A | 0.18 | 1.03 (1.01-1.06) | 0.01 | 1.02 (0.99-1.05) | 1.11 (1.03-1.2) | 0.02 |
| rs629480 | 110741144 | A,G | 0.12 | 1.02 (0.99-1.06) | 0.12 | 1.02 (0.98-1.05) | 1.1 (0.98-1.24) | 0.18 |
| rs79634968 | 110742192 | G,C | 0.10 | 1 (0.97-1.03) | 0.88 | 1 (0.96-1.03) | 0.99 (0.86-1.15) | 0.99 |
| rs657659 | 110742851 | A,G | 0.45 | 1 (0.98-1.02) | 0.96 | 1.02 (0.99-1.05) | 1 (0.96-1.04) | 0.35 |
| rs35871706 | 110745668 | G,A | 0.05 | 1.06 (1.01-1.1) | 0.01 | 1.05 (1-1.1) | 1.23 (0.96-1.59) | 0.03 |
| rs7024363 | 110746405 | A,C | 0.37 | 0.98 (0.96-1) | 0.03 | 0.98 (0.95-1.01) | 0.96 (0.92-1) | 0.10 |
| rs1556939 | 110749585 | C,G | 0.43 | 0.99 (0.97-1.01) | 0.22 | 0.99 (0.96-1.02) | 0.97 (0.94-1.01) | 0.44 |
| rs7029391 | 110751144 | A,G | 0.32 | 0.99 (0.97-1.01) | 0.17 | 0.99 (0.97-1.02) | 0.96 (0.92-1.01) | 0.29 |
| rs113203549 | 110751393 | G,A | 0.01 | 0.98 (0.88-1.08) | 0.64 | #N/A | #N/A | #N/A |
| rs480113 | 110751682 | G,A | 0.24 | 1.01 (0.99-1.03) | 0.32 | 1.01 (0.98-1.04) | 1.03 (0.97-1.09) | 0.57 |
| rs1009813 | 110752106 | G,A | 0.25 | 0.97 (0.95-1) | 0.02 | 0.98 (0.95-1.01) | 0.94 (0.89-1) | 0.06 |
| rs7851574 | 110753079 | A,G | 0.08 | 1.04 (1-1.08) | 0.03 | 1.05 (1.01-1.1) | 0.95 (0.8-1.13) | 0.03 |
| rs1934419 | 110754102 | A,G | 0.19 | 1.02 (0.99-1.04) | 0.19 | 1.01 (0.98-1.05) | 1.04 (0.97-1.12) | 0.41 |
| rs1769652 | 110754778 | A,G | 0.36 | 0.98 (0.96-1) | 0.12 | 0.99 (0.96-1.02) | 0.96 (0.92-1.01) | 0.27 |
| rs10979155 | 110754824 | A,G | 0.01 | 1.03 (0.95-1.13) | 0.47 | 1.04 (0.95-1.13) | 0.79 (0.28-2.23) | 0.65 |
| rs75358334 | 110755497 | G,A | 0.12 | 1 (0.97-1.03) | 0.82 | 1 (0.96-1.03) | 1.05 (0.94-1.18) | 0.67 |
| rs10979157 | 110757887 | G,A | 0.07 | 1.04 (1-1.08) | 0.05 | 1.05 (1.01-1.09) | 0.97 (0.78-1.19) | 0.08 |
| rs76449292 | 110758841 | C,G | 0.12 | 0.99 (0.96-1.02) | 0.46 | 0.98 (0.95-1.01) | 1.05 (0.94-1.17) | 0.28 |
| rs13288365 | 110759721 | G,C | 0.33 | 1.04 (1.02-1.07) | 3.68E-05 | 1.05 (1.02-1.08) | 1.08 (1.04-1.14) | 1.79E-04 |
| rs673985 | 110759922 | G,A | 0.36 | 0.94 (0.92-0.96) | 9.68E-09 | 0.96 (0.93-0.99) | 0.88 (0.84-0.92) | 2.43E-08 |
| rs10979158 | 110760000 | A,G | 0.48 | 0.94 (0.92-0.96) | 9.62E-10 | 0.95 (0.92-0.98) | 0.89 (0.85-0.92) | 7.23E-09 |
| rs4979617 | 110760144 | G,A | 0.48 | 0.94 (0.92-0.96) | 1.38E-09 | 0.94 (0.91-0.98) | 0.89 (0.85-0.92) | 1.06E-08 |
| rs4979618 | 110760316 | G,A | 0.44 | 1.06 (1.04-1.08) | 3.63E-09 | 1.06 (1.03-1.09) | 1.12 (1.08-1.17) | 2.74E-08 |
| rs4979620 | 110760910 | G,C | 0.44 | 1.07 (1.05-1.09) | 1.01E-10 | 1.07 (1.04-1.1) | 1.14 (1.09-1.18) | 8.28E-10 |
| rs12342578 | 110761002 | G,A | 0.09 | 1.05 (1.02-1.09) | 1.71E-03 | 1.06 (1.02-1.09) | 1.11 (0.96-1.29) | 7.32E-03 |
| rs12342611 | 110761104 | C,G | 0.48 | 0.94 (0.92-0.96) | 1.29E-10 | 0.94 (0.91-0.97) | 0.88 (0.85-0.92) | 1.07E-09 |
| rs12347482 | 110761427 | A,C | 0.44 | 1.07 (1.05-1.09) | 8.80E-11 | 1.07 (1.03-1.1) | 1.14 (1.09-1.18) | 7.31E-10 |
| rs12236795 | 110761513 | G,A | 0.43 | 1.07 (1.05-1.09) | 1.24E-10 | 1.07 (1.03-1.1) | 1.14 (1.09-1.18) | 1.03E-09 |
| rs4979621 | 110764599 | G,A | 0.42 | 1.07 (1.05-1.09) | 1.06E-10 | 1.07 (1.04-1.1) | 1.13 (1.09-1.18) | 8.19E-10 |
| rs4979622 | 110764799 | A,G | 0.44 | 1.06 (1.04-1.08) | 5.17E-10 | 1.07 (1.04-1.1) | 1.13 (1.09-1.17) | 3.90E-09 |
| rs4978665 | 110764844 | G,C | 0.24 | 1.07 (1.04-1.09) | 7.79E-09 | 1.09 (1.06-1.12) | 1.1 (1.04-1.17) | 1.21E-08 |
| rs10759270 | 110764981 | G,A | 0.10 | 1.05 (1.01-1.08) | 6.15E-03 | 1.05 (1.01-1.08) | 1.08 (0.95-1.24) | 0.02 |
| rs12554275 | 110765067 | C,G | 0.08 | 1.01 (0.97-1.04) | 0.64 | 1.01 (0.98-1.05) | 0.95 (0.81-1.12) | 0.63 |
| rs77413190 | 110765278 | A,G | 0.04 | 0.94 (0.89-0.98) | 6.22E-03 | 0.93 (0.88-0.97) | 1.13 (0.81-1.56) | 6.89E-03 |
| rs10512369 | 110765359 | G,A | 0.22 | 0.95 (0.93-0.98) | 7.03E-05 | 0.98 (0.95-1.01) | 0.85 (0.8-0.91) | 4.37E-06 |
| rs1416836 | 110766963 | G,A | 0.32 | 1.05 (1.03-1.07) | 1.07E-05 | 1.06 (1.03-1.09) | 1.08 (1.03-1.13) | 2.38E-05 |
| rs9299144 | 110767478 | A,G | 0.43 | 1.06 (1.04-1.08) | 6.62E-10 | 1.07 (1.04-1.1) | 1.13 (1.08-1.17) | 4.79E-09 |
| rs10120885 | 110768664 | G,C | 0.49 | 1.07 (1.05-1.09) | 6.71E-11 | 1.06 (1.03-1.1) | 1.14 (1.09-1.18) | 5.22E-10 |
| rs4979623 | 110769283 | G,A | 0.41 | 1.07 (1.05-1.09) | 9.07E-11 | 1.07 (1.04-1.1) | 1.14 (1.09-1.18) | 7.51E-10 |
| rs9695201 | 110769468 | A,G | 0.41 | 1.07 (1.05-1.09) | 1.83E-10 | 1.07 (1.04-1.1) | 1.14 (1.09-1.18) | 1.49E-09 |
| rs7860780 | 110772665 | A,G | 0.41 | 1.07 (1.05-1.09) | 1.23E-10 | 1.07 (1.04-1.1) | 1.14 (1.09-1.18) | 1.01E-09 |
| rs9695286 | 110773067 | A,G | 0.50 | 1.06 (1.04-1.09) | 1.43E-10 | 1.06 (1.03-1.1) | 1.13 (1.09-1.18) | 1.15E-09 |
| rs77796391 | 110774115 | G,A | 0.03 | 0.91 (0.86-0.97) | 4.58E-03 | 0.92 (0.87-0.98) | 0.61 (0.37-1.02) | 8.30E-03 |
| rs62568532 | 110775973 | G,A | 0.10 | 1.06 (1.02-1.09) | 5.50E-04 | 1.05 (1.02-1.09) | 1.14 (1-1.3) | 2.46E-03 |
| rs1339756 | 110776765 | A,G | 0.41 | 1.07 (1.04-1.09) | 1.99E-10 | 1.06 (1.03-1.1) | 1.14 (1.09-1.18) | 1.62E-09 |
| rs10979163 | 110780615 | G,A | 0.30 | 1.06 (1.04-1.09) | 1.38E-08 | 1.07 (1.04-1.1) | 1.12 (1.06-1.17) | 7.18E-08 |
| rs7029094 | 110782565 | A,G | 0.41 | 1.07 (1.05-1.09) | 1.01E-10 | 1.07 (1.04-1.1) | 1.14 (1.09-1.18) | 8.31E-10 |
| rs10512368 | 110783036 | G,A | 0.41 | 1.07 (1.05-1.09) | 1.60E-10 | 1.07 (1.04-1.1) | 1.14 (1.09-1.18) | 1.30E-09 |
| rs10816612 | 110786222 | G,A | 0.41 | 1.07 (1.05-1.09) | 7.10E-11 | 1.07 (1.04-1.1) | 1.14 (1.09-1.19) | 5.89E-10 |
| rs4979625 | 110786533 | A,T | 0.41 | 1.07 (1.05-1.09) | 1.16E-10 | 1.07 (1.03-1.1) | 1.14 (1.09-1.18) | 9.56E-10 |
| rs7868720 | 110787096 | G,A | 0.41 | 1.07 (1.05-1.09) | 1.37E-10 | 1.07 (1.04-1.1) | 1.14 (1.09-1.18) | 1.12E-09 |
| rs1995761 | 110795237 | A,G | 0.41 | 1.07 (1.05-1.09) | 1.02E-10 | 1.07 (1.04-1.1) | 1.14 (1.09-1.18) | 8.41E-10 |
| rs10759272 | 110795806 | C,G | 0.41 | 1.07 (1.05-1.09) | 1.47E-10 | 1.07 (1.04-1.1) | 1.14 (1.09-1.18) | 1.18E-09 |
| rs11789909 | 110796768 | T,A | 0.08 | 0.91 (0.88-0.94) | 9.05E-08 | 0.92 (0.88-0.95) | 0.74 (0.63-0.88) | 2.35E-07 |
| rs12377186 | 110797181 | A,G | 0.11 | 0.98 (0.95-1.01) | 0.17 | 0.99 (0.96-1.03) | 0.88 (0.78-0.99) | 0.09 |
| rs10979179 | 110805072 | G,A | 0.11 | 1.05 (1.02-1.08) | 2.69E-03 | 1.05 (1.01-1.09) | 1.11 (0.98-1.26) | 0.01 |
| rs7020797 | 110806917 | G,A | 0.40 | 1.07 (1.05-1.09) | 8.80E-12 | 1.07 (1.04-1.1) | 1.15 (1.1-1.19) | 7.64E-11 |
| rs10816621 | 110808571 | G,A | 0.07 | 1.05 (1.01-1.09) | 0.01 | 1.06 (1.02-1.11) | 0.96 (0.78-1.17) | 0.01 |
| rs13301510 | 110809708 | G,A | 0.29 | 1.06 (1.04-1.08) | 4.49E-08 | 1.06 (1.03-1.09) | 1.12 (1.07-1.18) | 3.08E-07 |
| rs6477612 | 110811552 | G,A | 0.41 | 1.07 (1.05-1.09) | 4.40E-11 | 1.07 (1.03-1.1) | 1.14 (1.1-1.19) | 3.66E-10 |
| rs6477613 | 110811614 | G,A | 0.41 | 1.07 (1.05-1.09) | 3.38E-11 | 1.07 (1.03-1.1) | 1.14 (1.1-1.19) | 2.81E-10 |
| rs1318148 | 110814693 | G,C | 0.41 | 1.07 (1.05-1.09) | 1.13E-12 | 1.07 (1.04-1.1) | 1.15 (1.11-1.2) | 9.60E-12 |
| rs892687 | 110814936 | A,C | 0.42 | 1.07 (1.05-1.09) | 2.83E-12 | 1.07 (1.04-1.1) | 1.15 (1.1-1.2) | 2.51E-11 |
| rs7861155 | 110815564 | G,C | 0.18 | 1.09 (1.06-1.11) | 4.51E-11 | 1.08 (1.05-1.12) | 1.2 (1.11-1.29) | 3.44E-10 |
| rs10979182 | 110817020 | A,G | 0.42 | 1.07 (1.05-1.09) | 1.66E-11 | 1.07 (1.03-1.1) | 1.14 (1.1-1.19) | 1.39E-10 |
| rs4978668 | 110817549 | C,A | 0.42 | 1.08 (1.05-1.1) | 3.34E-13 | 1.07 (1.04-1.1) | 1.16 (1.11-1.2) | 2.99E-12 |
| rs117323246 | 110819521 | A,G | 0.04 | 0.88 (0.84-0.93) | 6.97E-07 | 0.89 (0.85-0.94) | 0.66 (0.48-0.92) | 2.69E-06 |
| rs10979183 | 110821050 | G,A | 0.42 | 1.07 (1.05-1.1) | 3.94E-13 | 1.07 (1.04-1.1) | 1.16 (1.11-1.21) | 3.37E-12 |
| rs1878600 | 110821830 | G,A | 0.31 | 1.07 (1.05-1.09) | 5.59E-10 | 1.07 (1.04-1.1) | 1.14 (1.09-1.19) | 4.40E-09 |
| rs1434836 | 110822658 | A,G | 0.42 | 1.07 (1.05-1.09) | 1.17E-11 | 1.07 (1.03-1.1) | 1.15 (1.1-1.19) | 9.62E-11 |
| rs10979184 | 110825659 | A,G | 0.11 | 1.05 (1.02-1.08) | 1.85E-03 | 1.05 (1.02-1.09) | 1.1 (0.97-1.25) | 7.85E-03 |
| rs34528190 | 110825688 | A,T | 0.12 | 1 (0.97-1.03) | 1.00 | 1 (0.97-1.04) | 0.98 (0.88-1.1) | 0.93 |
| rs4978670 | 110826546 | A,G | 0.42 | 1.07 (1.05-1.09) | 3.05E-12 | 1.07 (1.04-1.1) | 1.15 (1.11-1.2) | 2.55E-11 |
| rs10816623 | 110828308 | A,G | 0.12 | 1.04 (1.01-1.07) | 6.81E-03 | 1.04 (1.01-1.08) | 1.09 (0.96-1.22) | 0.03 |
| rs13289116 | 110829012 | A,G | 0.31 | 1.06 (1.03-1.08) | 4.91E-07 | 1.06 (1.03-1.09) | 1.11 (1.06-1.17) | 3.16E-06 |
| rs13291052 | 110832584 | A,G | 0.31 | 1.06 (1.04-1.08) | 5.24E-08 | 1.06 (1.03-1.09) | 1.12 (1.07-1.18) | 3.66E-07 |
| rs13292389 | 110832663 | A,G | 0.04 | 1.12 (1.06-1.17) | 9.94E-06 | 1.13 (1.07-1.18) | 1.05 (0.77-1.43) | 3.07E-05 |
| rs61538103 | 110833526 | C,A | 0.10 | 1 (0.97-1.04) | 0.83 | 1 (0.97-1.04) | 1.01 (0.88-1.17) | 0.97 |
| rs72745932 | 110834549 | A,G | 0.05 | 1.09 (1.04-1.15) | 1.73E-04 | 1.1 (1.05-1.15) | 1.07 (0.79-1.43) | 6.29E-04 |
| rs111283159 | 110835980 | C,A | 0.01 | 0.99 (0.9-1.08) | 0.75 | 0.98 (0.89-1.08) | 1.39 (0.44-4.4) | 0.79 |
| rs10481656 | 110836284 | G,A | 0.39 | 1.07 (1.05-1.09) | 5.77E-12 | 1.07 (1.04-1.1) | 1.15 (1.1-1.2) | 4.94E-11 |
| rs10481657 | 110836385 | G,A | 0.29 | 1.07 (1.05-1.09) | 5.53E-10 | 1.07 (1.04-1.1) | 1.15 (1.09-1.21) | 4.01E-09 |
| rs10481658 | 110836416 | G,A | 0.39 | 1.07 (1.05-1.09) | 6.40E-12 | 1.07 (1.04-1.1) | 1.15 (1.1-1.2) | 5.55E-11 |
| **rs10816625** | **110837073** | **A,G** | **0.07** | **1.12 (1.08-1.17)** | **7.89E-09** | **1.14 (1.09-1.19)** | **0.99 (0.8-1.24)** | **4.30E-09** |
| **rs13294895** | **110837176** | **G,A** | **0.18** | **1.09 (1.06-1.12)** | **2.97E-11** | **1.08 (1.05-1.11)** | **1.23 (1.13-1.32)** | **1.35E-10** |
| rs35909461 | 110838269 | A,G | 0.09 | 1.02 (0.98-1.05) | 0.32 | 1.03 (0.99-1.06) | 0.97 (0.83-1.12) | 0.37 |
| rs7042321 | 110841593 | G,A | 0.08 | 1.02 (0.99-1.06) | 0.17 | 1.03 (0.99-1.07) | 1 (0.85-1.18) | 0.32 |
| rs10118883 | 110843927 | A,G | 0.10 | 1.01 (0.98-1.04) | 0.62 | 1.02 (0.99-1.06) | 0.9 (0.79-1.03) | 0.11 |
| rs10979190 | 110845216 | G,A | 0.06 | 1.01 (0.96-1.05) | 0.79 | 1.01 (0.96-1.05) | 1 (0.8-1.27) | 0.96 |
| rs10979202 | 110849566 | A,C | 0.06 | 0.99 (0.96-1.03) | 0.78 | 1 (0.96-1.04) | 0.94 (0.76-1.17) | 0.87 |
| rs10979204 | 110850362 | G,C | 0.06 | 1 (0.96-1.04) | 0.98 | 1 (0.96-1.05) | 0.96 (0.77-1.2) | 0.92 |
| rs75004812 | 110853569 | A,G | 0.07 | 1.08 (1.04-1.12) | 6.09E-05 | 1.07 (1.03-1.11) | 1.31 (1.09-1.56) | 1.23E-04 |
| rs12236285 | 110855243 | G,A | 0.06 | 1 (0.96-1.04) | 0.92 | 1 (0.96-1.05) | 0.98 (0.78-1.23) | 0.97 |
| rs117557721 | 110855537 | G,A | 0.01 | 1.05 (0.95-1.16) | 0.35 | 1.04 (0.94-1.15) | 2.04 (0.69-6.02) | 0.33 |
| rs1434851 | 110857960 | A,G | 0.19 | 1.02 (0.99-1.04) | 0.18 | 1.04 (1.01-1.07) | 0.96 (0.89-1.03) | 9.84E-03 |
| rs1813421 | 110858823 | G,A | 0.24 | 1.02 (0.99-1.04) | 0.17 | 1.04 (1.01-1.07) | 0.98 (0.93-1.04) | 0.02 |
| rs7869753 | 110864852 | C,A | 0.24 | 1.02 (0.99-1.04) | 0.14 | 1.04 (1.01-1.07) | 0.98 (0.93-1.04) | 8.39E-03 |
| rs78833899 | 110866666 | A,G | 0.06 | 0.99 (0.95-1.03) | 0.74 | 1 (0.96-1.04) | 0.94 (0.76-1.17) | 0.84 |
| rs10979209 | 110868281 | G,C | 0.06 | 1.04 (1-1.08) | 0.05 | 1.05 (1.01-1.09) | 0.97 (0.78-1.2) | 0.08 |
| rs78417418 | 110871005 | A,G | 0.05 | 1.01 (0.97-1.06) | 0.60 | 1.01 (0.96-1.06) | 1.05 (0.79-1.4) | 0.86 |
| rs10979214 | 110871414 | G,A | 0.35 | 1.05 (1.03-1.07) | 3.35E-06 | 1.05 (1.02-1.08) | 1.1 (1.05-1.15) | 1.96E-05 |
| rs7042864 | 110872307 | G,A | 0.35 | 1.05 (1.03-1.07) | 3.99E-06 | 1.05 (1.02-1.08) | 1.1 (1.05-1.15) | 2.36E-05 |
| rs10124038 | 110872527 | A,G | 0.45 | 0.95 (0.93-0.97) | 3.49E-08 | 0.95 (0.93-0.98) | 0.9 (0.86-0.93) | 2.04E-07 |
| rs2043486 | 110873028 | A,G | 0.30 | 1.05 (1.03-1.07) | 5.14E-06 | 1.05 (1.02-1.08) | 1.1 (1.05-1.16) | 3.06E-05 |
| rs62569845 | 110874785 | A,G | 0.13 | 1 (0.97-1.03) | 0.97 | 1.02 (0.99-1.05) | 0.89 (0.8-1) | 0.06 |
| rs10979217 | 110875895 | A,G | 0.08 | 1.03 (0.99-1.07) | 0.10 | 1.03 (0.99-1.07) | 1.08 (0.9-1.29) | 0.24 |
| rs1999456 | 110877304 | G,C | 0.45 | 0.95 (0.93-0.97) | 5.10E-08 | 0.96 (0.93-0.99) | 0.9 (0.86-0.93) | 2.10E-07 |
| rs1999457 | 110877440 | A,T | 0.45 | 0.95 (0.93-0.96) | 1.74E-08 | 0.96 (0.93-0.99) | 0.89 (0.86-0.93) | 6.52E-08 |
| rs837983 | 110881453 | A,G | 0.37 | 0.9 (0.88-0.92) | 3.38E-25 | 0.89 (0.87-0.92) | 0.82 (0.78-0.85) | 3.22E-24 |
| rs10979219 | 110881731 | A,G | 0.24 | 1.08 (1.05-1.1) | 3.92E-11 | 1.09 (1.06-1.12) | 1.15 (1.08-1.22) | 2.58E-10 |
| rs667052 | 110882390 | A,G | 0.48 | 1.08 (1.06-1.1) | 7.96E-14 | 1.08 (1.05-1.12) | 1.16 (1.11-1.2) | 6.81E-13 |
| rs680138 | 110882980 | A,G | 0.37 | 0.9 (0.88-0.92) | 5.29E-25 | 0.89 (0.86-0.92) | 0.82 (0.78-0.85) | 4.09E-24 |
| rs12376798 | 110883675 | G,A | 0.24 | 1.08 (1.05-1.1) | 1.18E-10 | 1.09 (1.06-1.12) | 1.14 (1.07-1.21) | 6.03E-10 |
| rs630965 | 110885479 | A,G | 0.37 | 0.9 (0.88-0.92) | 2.77E-25 | 0.89 (0.86-0.92) | 0.82 (0.78-0.85) | 1.92E-24 |
| rs631475 | 110885650 | A,C | 0.37 | 0.9 (0.88-0.92) | 3.10E-25 | 0.89 (0.86-0.92) | 0.82 (0.78-0.85) | 2.20E-24 |
| rs497006 | 110885781 | A,G | 0.37 | 0.9 (0.88-0.92) | 7.86E-25 | 0.89 (0.86-0.91) | 0.82 (0.79-0.86) | 4.38E-24 |
| rs519679 | 110885947 | G,C | 0.37 | 0.9 (0.88-0.92) | 3.35E-25 | 0.89 (0.86-0.91) | 0.82 (0.78-0.85) | 1.96E-24 |
| rs520613 | 110886052 | A,G | 0.37 | 0.9 (0.88-0.92) | 2.15E-24 | 0.89 (0.86-0.91) | 0.82 (0.78-0.85) | 1.27E-23 |
| rs522463 | 110886254 | A,C | 0.37 | 0.9 (0.88-0.92) | 1.56E-24 | 0.89 (0.86-0.92) | 0.82 (0.79-0.86) | 9.20E-24 |
| rs12380317 | 110886306 | T,A | 0.24 | 1.08 (1.06-1.1) | 3.65E-11 | 1.09 (1.06-1.12) | 1.14 (1.08-1.21) | 1.86E-10 |
| rs525142 | 110886534 | A,G | 0.37 | 0.9 (0.88-0.92) | 1.24E-24 | 0.89 (0.86-0.91) | 0.82 (0.79-0.86) | 6.83E-24 |
| rs62569853 | 110886727 | A,G | 0.09 | 1.04 (1.01-1.08) | 0.01 | 1.04 (1-1.08) | 1.14 (0.98-1.32) | 0.04 |
| rs527071 | 110886745 | A,C | 0.37 | 0.9 (0.88-0.92) | 9.75E-25 | 0.89 (0.86-0.92) | 0.82 (0.79-0.86) | 5.80E-24 |
| rs548980 | 110886840 | A,G | 0.37 | 0.9 (0.88-0.92) | 2.45E-25 | 0.89 (0.86-0.92) | 0.82 (0.78-0.85) | 1.86E-24 |
| rs1025573 | 110887338 | G,A | 0.09 | 1.04 (1.01-1.08) | 0.02 | 1.04 (1-1.08) | 1.12 (0.97-1.31) | 0.05 |
| rs580018 | 110887934 | A,G | 0.19 | 1.01 (0.99-1.04) | 0.28 | 1.03 (1-1.06) | 0.97 (0.9-1.04) | 0.06 |
| rs471467 | 110888113 | A,G | 0.37 | 0.9 (0.88-0.92) | 5.27E-25 | 0.89 (0.86-0.91) | 0.82 (0.78-0.85) | 3.32E-24 |
| rs472483 | 110888260 | G,A | 0.37 | 0.9 (0.88-0.92) | 1.17E-24 | 0.89 (0.86-0.92) | 0.82 (0.78-0.86) | 7.39E-24 |
| rs865686 | 110888478 | A,C | 0.37 | 0.9 (0.88-0.92) | 5.99E-25 | 0.89 (0.86-0.91) | 0.82 (0.78-0.85) | 3.60E-24 |
| rs7026944 | 110891253 | A,G | 0.24 | 1.08 (1.05-1.1) | 4.99E-11 | 1.09 (1.06-1.12) | 1.14 (1.07-1.21) | 2.22E-10 |
| rs7870781 | 110891494 | A,G | 0.32 | 1.09 (1.06-1.11) | 6.30E-15 | 1.08 (1.05-1.11) | 1.18 (1.13-1.24) | 6.07E-14 |
| rs6477617 | 110892030 | G,C | 0.32 | 1.08 (1.06-1.11) | 1.14E-14 | 1.08 (1.05-1.12) | 1.18 (1.12-1.23) | 1.12E-13 |
| rs510294 | 110892787 | A,T | 0.37 | 0.9 (0.88-0.92) | 1.80E-24 | 0.89 (0.86-0.92) | 0.82 (0.79-0.86) | 1.19E-23 |
| rs7862747 | 110892899 | A,C | 0.37 | 0.9 (0.88-0.92) | 4.04E-25 | 0.89 (0.87-0.92) | 0.82 (0.78-0.85) | 3.80E-24 |
| rs628931 | 110893030 | G,A | 0.37 | 0.9 (0.88-0.92) | 6.13E-25 | 0.89 (0.86-0.92) | 0.82 (0.78-0.85) | 4.24E-24 |
| rs10979226 | 110893793 | A,T | 0.24 | 1.08 (1.05-1.1) | 9.06E-11 | 1.09 (1.06-1.12) | 1.13 (1.07-1.2) | 3.72E-10 |
| rs10979227 | 110893829 | A,G | 0.24 | 1.08 (1.05-1.1) | 9.52E-11 | 1.09 (1.06-1.12) | 1.13 (1.07-1.2) | 3.93E-10 |
| rs659713 | 110893949 | C,A | 0.37 | 0.9 (0.88-0.92) | 3.09E-25 | 0.89 (0.86-0.91) | 0.82 (0.78-0.85) | 1.45E-24 |
| rs34137313 | 110893956 | A,C | 0.24 | 1.08 (1.05-1.1) | 1.15E-10 | 1.09 (1.06-1.12) | 1.13 (1.07-1.2) | 4.25E-10 |
| rs7047840 | 110894289 | A,G | 0.24 | 1.08 (1.05-1.1) | 1.32E-10 | 1.09 (1.06-1.12) | 1.13 (1.07-1.2) | 5.32E-10 |
| rs7048206 | 110894366 | G,A | 0.24 | 1.08 (1.05-1.1) | 9.01E-11 | 1.09 (1.06-1.12) | 1.13 (1.07-1.2) | 3.74E-10 |
| rs7033853 | 110894500 | A,G | 0.24 | 1.08 (1.05-1.1) | 8.19E-11 | 1.09 (1.06-1.12) | 1.13 (1.07-1.2) | 3.48E-10 |
| rs72745949 | 110894773 | G,A | 0.02 | 1.07 (0.99-1.16) | 0.10 | 1.06 (0.98-1.15) | 1.61 (0.7-3.69) | 0.18 |
| rs10979229 | 110895080 | G,A | 0.24 | 1.08 (1.05-1.1) | 3.99E-11 | 1.09 (1.06-1.12) | 1.14 (1.07-1.21) | 1.95E-10 |
| rs484167 | 110895137 | A,G | 0.24 | 1.03 (1.01-1.05) | 0.01 | 1.05 (1.02-1.08) | 1.01 (0.95-1.07) | 2.83E-03 |
| **rs676256** | **110895353** | **A,G** | **0.37** | **0.9 (0.88-0.92)** | **1.58E-25** | **0.89 (0.86-0.92)** | **0.81 (0.78-0.85)** | **1.32E-24** |
| rs55801097 | 110895455 | G,A | 0.01 | 0.93 (0.82-1.06) | 0.27 | #N/A | #N/A | #N/A |
| rs10979230 | 110895509 | C,A | 0.24 | 1.08 (1.05-1.1) | 6.15E-11 | 1.09 (1.06-1.12) | 1.14 (1.07-1.21) | 3.31E-10 |
| rs487887 | 110895527 | A,C | 0.25 | 1.03 (1.01-1.06) | 5.11E-03 | 1.05 (1.02-1.08) | 1.02 (0.97-1.08) | 1.49E-03 |
| rs10979231 | 110895634 | A,G | 0.24 | 1.08 (1.05-1.1) | 5.41E-11 | 1.09 (1.06-1.12) | 1.14 (1.07-1.21) | 2.84E-10 |
| rs10979233 | 110895705 | C,G | 0.24 | 1.08 (1.05-1.1) | 1.18E-10 | 1.09 (1.06-1.12) | 1.13 (1.07-1.2) | 5.23E-10 |
| rs3119744 | 110895863 | C,A | 0.37 | 0.9 (0.88-0.92) | 2.62E-25 | 0.89 (0.86-0.91) | 0.82 (0.78-0.85) | 1.74E-24 |
| rs1434838 | 110896141 | G,A | 0.24 | 1.08 (1.05-1.1) | 6.67E-11 | 1.09 (1.06-1.12) | 1.14 (1.07-1.21) | 3.37E-10 |
| rs1434839 | 110896230 | G,A | 0.24 | 1.08 (1.05-1.1) | 7.06E-11 | 1.09 (1.06-1.12) | 1.14 (1.07-1.21) | 3.43E-10 |
| rs516327 | 110896340 | G,A | 0.25 | 1.03 (1.01-1.06) | 5.99E-03 | 1.06 (1.03-1.09) | 1.02 (0.96-1.08) | 1.13E-03 |
| rs34793195 | 110896525 | A,G | 0.24 | 1.08 (1.05-1.1) | 1.50E-10 | 1.09 (1.06-1.12) | 1.14 (1.07-1.21) | 7.53E-10 |
| rs10979234 | 110896675 | G,A | 0.24 | 1.08 (1.05-1.1) | 7.18E-11 | 1.09 (1.06-1.12) | 1.14 (1.07-1.21) | 3.88E-10 |
| rs520093 | 110896775 | A,G | 0.39 | 1.05 (1.03-1.07) | 8.01E-06 | 1.06 (1.02-1.09) | 1.09 (1.04-1.13) | 3.38E-05 |
| rs595811 | 110896828 | G,A | 0.39 | 1.05 (1.03-1.07) | 1.81E-06 | 1.06 (1.03-1.09) | 1.1 (1.05-1.14) | 8.56E-06 |
| rs2053148 | 110898048 | C,A | 0.24 | 1.08 (1.05-1.1) | 2.27E-10 | 1.08 (1.05-1.12) | 1.14 (1.07-1.21) | 1.28E-09 |
| rs2117466 | 110898052 | G,A | 0.24 | 1.08 (1.05-1.1) | 1.01E-10 | 1.09 (1.06-1.12) | 1.14 (1.07-1.21) | 5.58E-10 |
| rs10979236 | 110898716 | A,G | 0.24 | 1.08 (1.05-1.1) | 2.91E-10 | 1.09 (1.06-1.12) | 1.13 (1.07-1.2) | 1.31E-09 |
| rs1836458 | 110898887 | A,C | 0.08 | 1.03 (0.99-1.07) | 0.12 | 1.03 (0.99-1.07) | 1.06 (0.89-1.27) | 0.31 |
| rs10979237 | 110898964 | G,A | 0.24 | 1.08 (1.05-1.1) | 2.29E-10 | 1.09 (1.06-1.12) | 1.13 (1.07-1.2) | 1.08E-09 |
| rs12379956 | 110899161 | A,G | 0.24 | 1.08 (1.05-1.1) | 2.28E-10 | 1.09 (1.06-1.12) | 1.13 (1.07-1.2) | 9.97E-10 |
| rs12379962 | 110899191 | A,G | 0.24 | 1.08 (1.05-1.1) | 3.08E-10 | 1.09 (1.05-1.12) | 1.13 (1.07-1.2) | 1.40E-09 |
| rs627610 | 110899291 | C,A | 0.24 | 1.02 (1-1.05) | 0.03 | 1.04 (1.01-1.07) | 1.01 (0.95-1.07) | 0.01 |
| rs10979238 | 110899409 | G,A | 0.24 | 1.08 (1.05-1.1) | 2.41E-10 | 1.09 (1.06-1.12) | 1.13 (1.07-1.2) | 9.90E-10 |
| rs10979239 | 110899675 | C,G | 0.24 | 1.08 (1.05-1.1) | 2.81E-10 | 1.09 (1.06-1.12) | 1.13 (1.07-1.2) | 1.27E-09 |
| rs1434840 | 110899937 | C,A | 0.24 | 1.08 (1.05-1.1) | 1.99E-10 | 1.09 (1.06-1.12) | 1.14 (1.07-1.21) | 9.98E-10 |
| rs641002 | 110900018 | G,A | 0.41 | 0.91 (0.89-0.93) | 3.45E-21 | 0.88 (0.86-0.91) | 0.84 (0.81-0.87) | 1.32E-21 |
| rs1434841 | 110900034 | G,A | 0.24 | 1.08 (1.05-1.1) | 1.71E-10 | 1.09 (1.06-1.12) | 1.13 (1.07-1.2) | 8.02E-10 |
| rs1434842 | 110900060 | A,G | 0.24 | 1.08 (1.05-1.1) | 1.95E-10 | 1.09 (1.06-1.12) | 1.13 (1.07-1.2) | 9.09E-10 |
| rs1836459 | 110900115 | G,A | 0.24 | 1.08 (1.05-1.1) | 9.15E-11 | 1.09 (1.06-1.12) | 1.14 (1.07-1.21) | 4.96E-10 |
| rs35338365 | 110900335 | G,A | 0.24 | 1.08 (1.05-1.1) | 1.50E-10 | 1.09 (1.06-1.12) | 1.14 (1.07-1.21) | 7.11E-10 |
| rs10979242 | 110900392 | G,A | 0.24 | 1.08 (1.05-1.1) | 8.50E-11 | 1.09 (1.06-1.12) | 1.14 (1.07-1.21) | 4.70E-10 |
| rs10979243 | 110900418 | G,A | 0.24 | 1.08 (1.05-1.1) | 1.49E-10 | 1.09 (1.06-1.12) | 1.13 (1.07-1.2) | 6.66E-10 |
| rs10979244 | 110900593 | T,A | 0.24 | 1.08 (1.05-1.1) | 1.44E-10 | 1.09 (1.06-1.12) | 1.14 (1.07-1.21) | 7.03E-10 |
| rs10979245 | 110900744 | G,A | 0.24 | 1.08 (1.05-1.1) | 1.48E-10 | 1.09 (1.06-1.12) | 1.14 (1.07-1.21) | 7.16E-10 |
| rs10979246 | 110901072 | G,C | 0.24 | 1.08 (1.05-1.1) | 1.28E-10 | 1.09 (1.06-1.12) | 1.14 (1.07-1.21) | 6.47E-10 |
| rs2571501 | 110902296 | A,T | 0.21 | 1.02 (1-1.04) | 0.12 | 1.04 (1.01-1.07) | 0.98 (0.92-1.05) | 0.02 |
| rs10979247 | 110903322 | A,G | 0.24 | 1.08 (1.05-1.1) | 1.89E-10 | 1.09 (1.06-1.12) | 1.13 (1.07-1.2) | 8.11E-10 |
| rs12380087 | 110903380 | C,A | 0.24 | 1.08 (1.05-1.1) | 1.89E-10 | 1.09 (1.05-1.12) | 1.14 (1.07-1.21) | 9.67E-10 |
| rs7853556 | 110903440 | A,G | 0.07 | 1.01 (0.97-1.05) | 0.54 | 1.01 (0.97-1.06) | 0.99 (0.8-1.22) | 0.78 |
| rs117686075 | 110903782 | A,G | 0.02 | 0.98 (0.91-1.06) | 0.62 | 0.98 (0.91-1.05) | 1.07 (0.54-2.14) | 0.84 |
| rs10979248 | 110904528 | A,G | 0.24 | 1.08 (1.05-1.1) | 4.14E-11 | 1.09 (1.06-1.12) | 1.14 (1.07-1.21) | 2.08E-10 |
| rs537744 | 110904645 | C,G | 0.42 | 0.91 (0.9-0.93) | 1.53E-19 | 0.89 (0.86-0.92) | 0.84 (0.81-0.88) | 1.46E-19 |
| rs567032 | 110905570 | A,G | 0.35 | 1.04 (1.02-1.06) | 4.77E-04 | 1.04 (1.01-1.07) | 1.07 (1.02-1.12) | 2.12E-03 |
| rs567960 | 110905663 | G,A | 0.27 | 1.02 (1-1.04) | 0.08 | 1.02 (0.99-1.05) | 1.04 (0.98-1.09) | 0.21 |
| rs479410 | 110905747 | A,T | 0.27 | 1.02 (1-1.04) | 0.09 | 1.02 (0.99-1.05) | 1.03 (0.98-1.09) | 0.23 |
| rs637397 | 110905812 | G,A | 0.42 | 0.91 (0.89-0.93) | 3.93E-20 | 0.89 (0.86-0.92) | 0.84 (0.81-0.88) | 4.38E-20 |
| rs59244432 | 110906257 | A,G | 0.08 | 1.07 (1.03-1.11) | 6.05E-04 | 1.07 (1.03-1.12) | 1.06 (0.88-1.28) | 2.02E-03 |
| rs651808 | 110906666 | A,G | 0.27 | 1.02 (1-1.04) | 0.10 | 1.02 (0.99-1.05) | 1.04 (0.98-1.09) | 0.25 |
| rs651829 | 110906677 | A,G | 0.42 | 0.91 (0.9-0.93) | 1.47E-19 | 0.89 (0.86-0.92) | 0.84 (0.81-0.88) | 1.72E-19 |
| rs10816634 | 110907407 | A,G | 0.24 | 1.08 (1.06-1.1) | 3.43E-11 | 1.09 (1.06-1.12) | 1.13 (1.07-1.2) | 1.20E-10 |
| rs10979249 | 110907520 | C,A | 0.24 | 1.08 (1.06-1.11) | 1.73E-11 | 1.09 (1.06-1.12) | 1.14 (1.08-1.21) | 7.72E-11 |
| rs10979251 | 110909319 | A,G | 0.24 | 1.08 (1.05-1.1) | 2.15E-10 | 1.08 (1.05-1.12) | 1.14 (1.07-1.21) | 1.31E-09 |
| rs10816635 | 110910044 | G,A | 0.24 | 1.08 (1.05-1.1) | 1.40E-10 | 1.08 (1.05-1.11) | 1.14 (1.08-1.22) | 9.30E-10 |
| rs602325 | 110910506 | T,A | 0.20 | 1.02 (1-1.05) | 0.07 | 1.04 (1.01-1.07) | 1 (0.93-1.07) | 0.03 |
| rs539270 | 110910792 | G,A | 0.27 | 1.02 (0.99-1.04) | 0.15 | 1.02 (0.99-1.05) | 1.03 (0.98-1.09) | 0.36 |
| rs10512365 | 110911118 | G,A | 0.24 | 1.08 (1.05-1.1) | 2.38E-10 | 1.08 (1.05-1.12) | 1.14 (1.07-1.21) | 1.40E-09 |
| rs542808 | 110911155 | G,A | 0.20 | 1.02 (1-1.05) | 0.10 | 1.04 (1.01-1.07) | 0.99 (0.92-1.06) | 0.04 |
| rs28496063 | 110911169 | G,A | 0.24 | 1.08 (1.05-1.1) | 1.88E-10 | 1.08 (1.05-1.12) | 1.14 (1.07-1.21) | 1.07E-09 |
| rs28550561 | 110911197 | A,G | 0.31 | 1.09 (1.06-1.11) | 3.66E-15 | 1.08 (1.05-1.11) | 1.2 (1.14-1.26) | 2.23E-14 |
| rs566510 | 110911435 | A,G | 0.42 | 0.92 (0.9-0.93) | 2.78E-18 | 0.9 (0.87-0.92) | 0.85 (0.82-0.88) | 4.16E-18 |
| rs10115125 | 110911908 | G,C | 0.24 | 1.08 (1.05-1.1) | 1.37E-10 | 1.08 (1.05-1.12) | 1.14 (1.08-1.21) | 8.66E-10 |
| rs633082 | 110912805 | C,G | 0.29 | 1.02 (1-1.04) | 0.10 | 1.03 (1-1.06) | 1.03 (0.97-1.08) | 0.19 |
| rs634454 | 110913154 | C,A | 0.20 | 0.91 (0.89-0.93) | 2.67E-14 | 0.91 (0.88-0.94) | 0.83 (0.77-0.89) | 2.59E-13 |
| rs635667 | 110913378 | A,G | 0.28 | 1.01 (0.99-1.03) | 0.46 | 1.01 (0.98-1.04) | 1.01 (0.96-1.07) | 0.73 |
| rs677872 | 110914540 | G,A | 0.41 | 0.92 (0.9-0.93) | 3.42E-18 | 0.89 (0.87-0.92) | 0.85 (0.82-0.89) | 2.71E-18 |
| rs586559 | 110915881 | A,G | 0.41 | 0.92 (0.9-0.94) | 2.06E-17 | 0.9 (0.87-0.92) | 0.85 (0.82-0.89) | 2.19E-17 |
| rs614851 | 110917627 | A,G | 0.28 | 1.01 (0.99-1.04) | 0.18 | 1.02 (0.99-1.05) | 1.02 (0.97-1.08) | 0.35 |
| rs10816636 | 110918637 | A,C | 0.24 | 1.08 (1.05-1.1) | 1.34E-10 | 1.09 (1.06-1.12) | 1.13 (1.07-1.2) | 6.04E-10 |
| rs79304677 | 110919211 | G,A | 0.03 | 0.84 (0.79-0.89) | 1.11E-08 | 0.84 (0.79-0.9) | 0.58 (0.35-0.96) | 6.04E-08 |
| rs34499892 | 110920289 | G,A | 0.02 | 1.09 (1.02-1.16) | 0.01 | #N/A | #N/A | #N/A |
| rs7850979 | 110920305 | G,A | 0.31 | 1.09 (1.07-1.11) | 7.39E-16 | 1.08 (1.05-1.11) | 1.2 (1.14-1.26) | 4.72E-15 |
| rs7866211 | 110920309 | A,G | 0.31 | 1.09 (1.07-1.11) | 5.85E-16 | 1.08 (1.05-1.11) | 1.2 (1.15-1.26) | 3.69E-15 |
| rs113741411 | 110920364 | A,T | 0.48 | 0.94 (0.92-0.96) | 4.25E-11 | 0.93 (0.9-0.96) | 0.88 (0.85-0.91) | 3.33E-10 |
| rs1434843 | 110920807 | G,A | 0.24 | 1.08 (1.05-1.1) | 8.73E-11 | 1.09 (1.06-1.12) | 1.14 (1.07-1.21) | 4.91E-10 |
| rs676083 | 110922092 | G,A | 0.41 | 0.92 (0.9-0.94) | 1.51E-17 | 0.9 (0.87-0.92) | 0.85 (0.82-0.89) | 2.24E-17 |
| rs10512363 | 110922828 | A,G | 0.24 | 1.07 (1.05-1.1) | 7.20E-10 | 1.08 (1.05-1.11) | 1.14 (1.07-1.21) | 4.43E-09 |
| rs525803 | 110923883 | A,G | 0.41 | 0.92 (0.9-0.94) | 7.61E-17 | 0.9 (0.87-0.93) | 0.86 (0.82-0.89) | 1.24E-16 |
| rs837993 | 110924767 | A,C | 0.41 | 0.92 (0.9-0.94) | 1.72E-16 | 0.9 (0.87-0.93) | 0.86 (0.82-0.89) | 2.25E-16 |
| rs500708 | 110925107 | A,G | 0.41 | 0.92 (0.9-0.94) | 4.40E-16 | 0.9 (0.87-0.93) | 0.86 (0.83-0.9) | 3.93E-16 |
| rs630611 | 110925407 | A,C | 0.37 | 0.91 (0.89-0.93) | 6.43E-20 | 0.89 (0.87-0.92) | 0.84 (0.81-0.88) | 1.19E-19 |
| rs563471 | 110925919 | A,G | 0.28 | 1.01 (0.99-1.03) | 0.28 | 1.01 (0.98-1.04) | 1.02 (0.97-1.08) | 0.56 |
| rs837994 | 110926669 | C,A | 0.37 | 0.91 (0.9-0.93) | 1.84E-18 | 0.9 (0.87-0.92) | 0.85 (0.81-0.88) | 5.12E-18 |
| rs116897613 | 110926751 | G,A | 0.05 | 0.96 (0.92-1) | 0.07 | 0.96 (0.91-1) | 0.99 (0.77-1.27) | 0.16 |
| rs7030021 | 110927072 | C,A | 0.24 | 1.07 (1.05-1.1) | 3.03E-09 | 1.07 (1.04-1.11) | 1.14 (1.07-1.21) | 2.18E-08 |
| rs13290910 | 110927519 | G,A | 0.24 | 1.07 (1.05-1.1) | 5.43E-10 | 1.07 (1.04-1.11) | 1.15 (1.09-1.22) | 4.32E-09 |
| rs510082 | 110927520 | G,A | 0.41 | 0.92 (0.9-0.94) | 4.27E-16 | 0.9 (0.87-0.93) | 0.86 (0.83-0.9) | 3.30E-16 |
| rs511142 | 110927695 | A,G | 0.41 | 0.92 (0.9-0.94) | 1.66E-16 | 0.89 (0.87-0.92) | 0.86 (0.83-0.9) | 7.44E-17 |
| rs671618 | 110927732 | C,A | 0.28 | 1.01 (0.99-1.03) | 0.29 | 1.01 (0.99-1.04) | 1.02 (0.97-1.08) | 0.55 |
| rs687992 | 110929030 | A,G | 0.28 | 1.01 (0.99-1.03) | 0.26 | 1.01 (0.98-1.04) | 1.02 (0.97-1.08) | 0.53 |
| rs7038715 | 110929199 | G,A | 0.31 | 1.09 (1.06-1.11) | 1.34E-14 | 1.07 (1.04-1.1) | 1.19 (1.14-1.25) | 7.00E-14 |
| rs7027772 | 110929605 | A,G | 0.31 | 1.09 (1.06-1.11) | 7.02E-15 | 1.08 (1.05-1.11) | 1.19 (1.14-1.25) | 5.19E-14 |
| rs593924 | 110929785 | A,T | 0.41 | 0.92 (0.9-0.94) | 9.55E-17 | 0.9 (0.87-0.93) | 0.86 (0.82-0.89) | 1.23E-16 |
| rs573308 | 110929842 | G,C | 0.41 | 0.92 (0.9-0.94) | 2.40E-16 | 0.9 (0.87-0.93) | 0.86 (0.82-0.89) | 3.15E-16 |
| rs527246 | 110932294 | G,A | 0.41 | 0.92 (0.9-0.94) | 1.84E-16 | 0.9 (0.87-0.92) | 0.86 (0.82-0.89) | 1.39E-16 |
| rs626290 | 110932412 | G,A | 0.28 | 1.01 (0.99-1.03) | 0.38 | 1.01 (0.98-1.04) | 1.02 (0.97-1.08) | 0.68 |
| rs477917 | 110936038 | G,C | 0.28 | 1.01 (0.99-1.03) | 0.32 | 1.01 (0.98-1.04) | 1.02 (0.97-1.08) | 0.60 |
| rs509311 | 110937131 | A,C | 0.28 | 1.01 (0.99-1.03) | 0.40 | 1.01 (0.98-1.04) | 1.02 (0.97-1.07) | 0.70 |
| rs10816637 | 110937650 | C,A | 0.28 | 1.01 (0.99-1.03) | 0.39 | 1.01 (0.98-1.04) | 1.02 (0.97-1.08) | 0.68 |
| rs1434844 | 110938877 | T,A | 0.31 | 1.08 (1.06-1.11) | 5.98E-14 | 1.07 (1.04-1.1) | 1.19 (1.14-1.25) | 1.82E-13 |
| rs78952179 | 110939082 | A,G | 0.07 | 1.04 (1-1.07) | 0.07 | 1.04 (1-1.08) | 1.06 (0.89-1.27) | 0.19 |
| rs10979261 | 110939138 | A,G | 0.24 | 1.07 (1.05-1.1) | 2.48E-09 | 1.07 (1.04-1.1) | 1.14 (1.08-1.21) | 1.88E-08 |
| rs117566834 | 110939166 | A,G | 0.01 | 1.05 (0.94-1.18) | 0.37 | #N/A | #N/A | #N/A |
| rs78025103 | 110939265 | G,A | 0.01 | 1.08 (0.98-1.19) | 0.10 | 1.07 (0.97-1.18) | 2.32 (0.71-7.56) | 0.13 |
| rs1434846 | 110939308 | A,G | 0.31 | 1.08 (1.06-1.1) | 9.99E-14 | 1.07 (1.04-1.1) | 1.19 (1.13-1.25) | 4.07E-13 |
| rs72745965 | 110939948 | G,C | 0.02 | 0.91 (0.85-0.98) | 0.01 | 0.92 (0.86-0.99) | 0.4 (0.16-1.02) | 0.01 |
| rs503238 | 110940013 | G,A | 0.27 | 1.01 (0.99-1.04) | 0.19 | 1.02 (0.99-1.05) | 1.03 (0.97-1.08) | 0.42 |
| rs10979262 | 110940990 | T,A | 0.31 | 1.08 (1.06-1.11) | 2.84E-14 | 1.07 (1.04-1.1) | 1.19 (1.14-1.25) | 1.48E-13 |
| rs12380632 | 110941095 | A,G | 0.33 | 1.08 (1.06-1.1) | 8.80E-14 | 1.07 (1.04-1.1) | 1.19 (1.13-1.24) | 3.60E-13 |
| rs558016 | 110941382 | G,A | 0.23 | 1.01 (0.99-1.04) | 0.27 | 1.02 (0.99-1.05) | 1.01 (0.95-1.07) | 0.37 |
| rs666303 | 110941390 | G,A | 0.31 | 0.93 (0.91-0.95) | 5.15E-11 | 0.93 (0.9-0.95) | 0.88 (0.84-0.92) | 3.06E-10 |
| rs77889939 | 110942176 | T,A | 0.27 | 1.01 (0.99-1.03) | 0.26 | 1.02 (0.99-1.05) | 1.02 (0.97-1.08) | 0.50 |
| rs683466 | 110942879 | G,A | 0.26 | 1.01 (0.99-1.03) | 0.41 | 1.02 (0.99-1.05) | 1 (0.94-1.05) | 0.28 |
| rs72745970 | 110943026 | G,A | 0.01 | 1.06 (0.98-1.15) | 0.17 | 1.06 (0.97-1.15) | 1.2 (0.44-3.27) | 0.38 |
| rs10979263 | 110943815 | A,G | 0.04 | 1.01 (0.96-1.07) | 0.59 | 1 (0.95-1.06) | 1.22 (0.89-1.66) | 0.47 |
| rs10979264 | 110944986 | G,A | 0.03 | 1 (0.95-1.05) | 0.98 | 0.98 (0.93-1.04) | 1.42 (1.01-2) | 0.09 |
| rs607019 | 110945296 | G,A | 0.26 | 1.01 (0.99-1.03) | 0.37 | 1.02 (0.99-1.05) | 1 (0.95-1.06) | 0.32 |
| rs607056 | 110945330 | G,A | 0.26 | 1.01 (0.99-1.03) | 0.37 | 1.02 (0.99-1.05) | 1 (0.95-1.06) | 0.33 |
| rs10759273 | 110946296 | A,G | 0.06 | 1.01 (0.97-1.05) | 0.67 | 1 (0.96-1.05) | 1.1 (0.88-1.36) | 0.70 |
| rs559836 | 110946380 | G,A | 0.26 | 1.01 (0.99-1.03) | 0.41 | 1.02 (0.99-1.05) | 1 (0.94-1.05) | 0.31 |
| rs10816640 | 110946857 | G,A | 0.04 | 1 (0.95-1.05) | 0.99 | 0.98 (0.93-1.04) | 1.42 (1.01-2) | 0.10 |
| rs553333 | 110947155 | G,A | 0.26 | 1.01 (0.99-1.03) | 0.34 | 1.02 (0.99-1.05) | 1 (0.95-1.06) | 0.27 |
| rs864145 | 110948023 | G,C | 0.26 | 1.01 (0.99-1.03) | 0.35 | 1.02 (0.99-1.05) | 1 (0.95-1.06) | 0.28 |
| rs7875207 | 110948812 | G,A | 0.01 | 1.05 (0.96-1.14) | 0.31 | 1.04 (0.95-1.14) | 1.39 (0.45-4.31) | 0.54 |
| rs79583876 | 110949055 | C,A | 0.02 | 0.94 (0.88-1.01) | 0.09 | 0.94 (0.88-1.01) | 0.94 (0.54-1.64) | 0.23 |
| rs10816642 | 110950488 | A,G | 0.03 | 1 (0.95-1.05) | 0.98 | 0.99 (0.94-1.05) | 1.22 (0.84-1.78) | 0.54 |
| rs7022714 | 110950572 | A,G | 0.23 | 1.03 (1.01-1.06) | 4.90E-03 | 1.04 (1.01-1.07) | 1.06 (0.99-1.12) | 0.02 |
| rs13295544 | 110953139 | A,G | 0.07 | 0.98 (0.94-1.01) | 0.23 | 0.97 (0.93-1.01) | 1.06 (0.89-1.28) | 0.22 |
| rs28450518 | 110953584 | T,A | 0.01 | 1.08 (0.98-1.18) | 0.11 | 1.08 (0.99-1.19) | 0.89 (0.33-2.35) | 0.25 |
| rs669329 | 110954331 | A,G | 0.42 | 0.95 (0.93-0.96) | 1.20E-08 | 0.94 (0.91-0.97) | 0.9 (0.86-0.93) | 7.12E-08 |
| rs550891 | 110954663 | C,A | 0.36 | 0.95 (0.93-0.97) | 2.04E-06 | 0.95 (0.93-0.98) | 0.91 (0.87-0.95) | 1.26E-05 |
| rs59860864 | 110954925 | C,A | 0.17 | 1.04 (1.02-1.07) | 8.20E-04 | 1.03 (1-1.06) | 1.14 (1.05-1.23) | 1.52E-03 |
| rs13287026 | 110955043 | G,A | 0.05 | 0.99 (0.95-1.03) | 0.60 | 0.97 (0.93-1.02) | 1.21 (0.95-1.53) | 0.16 |
| rs556399 | 110955288 | G,A | 0.47 | 1.02 (1-1.04) | 0.06 | 1.02 (0.98-1.05) | 1.04 (1-1.08) | 0.17 |
| rs34979687 | 110955816 | A,G | 0.15 | 1.04 (1.01-1.07) | 6.73E-03 | 1.03 (1-1.06) | 1.12 (1.02-1.22) | 0.02 |
| rs638978 | 110956479 | G,A | 0.43 | 1.03 (1.01-1.05) | 7.04E-04 | 1.03 (1-1.06) | 1.07 (1.03-1.11) | 3.12E-03 |
| rs75802750 | 110956500 | G,A | 0.14 | 1.04 (1.02-1.07) | 2.23E-03 | 1.04 (1.01-1.08) | 1.1 (1-1.21) | 9.14E-03 |
| rs625647 | 110957231 | A,C | 0.46 | 1.02 (1-1.04) | 0.07 | 1.03 (0.99-1.06) | 1.03 (1-1.08) | 0.17 |
| rs3983564 | 110957368 | T,A | 0.42 | 1.03 (1.01-1.05) | 2.14E-03 | 1.03 (1-1.06) | 1.06 (1.02-1.11) | 8.96E-03 |
| rs507584 | 110957372 | T,A | 0.41 | 1.03 (1.01-1.05) | 3.40E-03 | 1.03 (1-1.06) | 1.06 (1.02-1.1) | 0.01 |
| rs538688 | 110958479 | A,C | 0.41 | 1.03 (1.01-1.05) | 4.42E-03 | 1.03 (1-1.06) | 1.06 (1.02-1.1) | 0.02 |
| rs10979270 | 110959027 | A,G | 0.08 | 1 (0.96-1.03) | 0.82 | 0.99 (0.95-1.03) | 1.03 (0.86-1.23) | 0.87 |
| rs569795 | 110959560 | G,A | 0.46 | 1.02 (1-1.04) | 0.06 | 1.03 (0.99-1.06) | 1.04 (1-1.08) | 0.14 |
| rs569859 | 110959588 | A,G | 0.46 | 1.02 (1-1.04) | 0.05 | 1.03 (1-1.06) | 1.04 (1-1.08) | 0.11 |
| rs581561 | 110960080 | C,A | 0.36 | 0.95 (0.93-0.97) | 1.16E-06 | 0.95 (0.93-0.98) | 0.9 (0.87-0.94) | 7.02E-06 |
| rs507242 | 110969263 | A,G | 0.41 | 1.03 (1.01-1.05) | 2.89E-03 | 1.03 (1-1.06) | 1.06 (1.02-1.11) | 0.01 |
| rs838013 | 110969574 | A,G | 0.41 | 1.03 (1.01-1.05) | 2.47E-03 | 1.03 (1-1.06) | 1.06 (1.02-1.11) | 0.01 |
| rs838012 | 110969777 | G,A | 0.41 | 1.03 (1.01-1.05) | 2.01E-03 | 1.03 (1-1.06) | 1.06 (1.02-1.11) | 8.46E-03 |
| rs10816648 | 110971140 | A,G | 0.05 | 0.95 (0.91-0.99) | 0.03 | 0.94 (0.89-0.98) | 1.15 (0.89-1.48) | 0.01 |
| rs35517812 | 110971386 | G,A | 0.17 | 1.04 (1.02-1.07) | 6.64E-04 | 1.04 (1.01-1.07) | 1.11 (1.03-1.2) | 2.57E-03 |
| rs485572 | 110972062 | C,G | 0.41 | 1.03 (1.01-1.05) | 3.16E-03 | 1.03 (1-1.06) | 1.06 (1.02-1.11) | 0.01 |
| rs539221 | 110972292 | A,C | 0.36 | 0.95 (0.94-0.97) | 4.63E-06 | 0.95 (0.93-0.98) | 0.91 (0.87-0.95) | 2.76E-05 |
| rs10979274 | 110973879 | A,G | 0.05 | 0.95 (0.91-1) | 0.03 | 0.94 (0.9-0.99) | 1.13 (0.87-1.46) | 0.02 |
| rs609982 | 110974043 | C,G | 0.46 | 1.02 (1-1.04) | 0.06 | 1.03 (1-1.06) | 1.04 (1-1.08) | 0.13 |
| rs73998 | 110974433 | C,A | 0.40 | 1.03 (1.01-1.05) | 4.07E-03 | 1.03 (1-1.06) | 1.06 (1.02-1.1) | 0.02 |
| rs10979275 | 110975315 | G,A | 0.10 | 0.97 (0.94-1) | 0.08 | 0.96 (0.92-0.99) | 1.07 (0.93-1.23) | 0.03 |
| rs548698 | 110975463 | C,A | 0.42 | 1.03 (1.01-1.05) | 4.62E-03 | 1.03 (1-1.06) | 1.06 (1.02-1.1) | 0.02 |
| rs838001 | 110975498 | C,A | 0.41 | 0.95 (0.93-0.97) | 1.06E-07 | 0.94 (0.91-0.97) | 0.9 (0.87-0.94) | 4.38E-07 |
| rs838002 | 110975878 | A,G | 0.42 | 1.03 (1.01-1.05) | 3.94E-03 | 1.03 (1-1.06) | 1.06 (1.02-1.1) | 0.02 |
| rs10979276 | 110975982 | A,G | 0.42 | 1.03 (1.01-1.05) | 4.29E-03 | 1.03 (1-1.06) | 1.06 (1.02-1.1) | 0.02 |
| rs73519684 | 110976133 | A,C | 0.17 | 1.04 (1.02-1.07) | 1.06E-03 | 1.04 (1.01-1.07) | 1.1 (1.02-1.19) | 4.28E-03 |
| rs498727 | 110976981 | G,A | 0.41 | 0.95 (0.93-0.97) | 8.66E-08 | 0.94 (0.91-0.97) | 0.9 (0.87-0.94) | 4.56E-07 |
| rs501345 | 110977231 | A,G | 0.41 | 0.95 (0.93-0.97) | 5.02E-08 | 0.94 (0.91-0.97) | 0.9 (0.87-0.94) | 2.44E-07 |
| rs656048 | 110977391 | G,A | 0.41 | 0.95 (0.93-0.97) | 8.22E-08 | 0.94 (0.91-0.97) | 0.9 (0.87-0.94) | 4.33E-07 |
| rs7026619 | 110978841 | A,C | 0.19 | 1.03 (1-1.05) | 0.05 | 1.03 (1-1.06) | 1.03 (0.95-1.1) | 0.10 |
| rs672586 | 110978859 | A,G | 0.34 | 1.02 (1-1.04) | 0.12 | 1.02 (0.99-1.05) | 1.03 (0.99-1.08) | 0.30 |
| rs4246873 | 110980594 | G,A | 0.38 | 0.95 (0.93-0.97) | 1.28E-06 | 0.95 (0.92-0.98) | 0.91 (0.87-0.95) | 6.98E-06 |
| rs62567152 | 110982252 | G,A | 0.11 | 1.02 (0.99-1.05) | 0.26 | 1.02 (0.99-1.06) | 1 (0.88-1.13) | 0.41 |
| rs71497837 | 110986274 | G,A | 0.02 | 0.93 (0.85-1) | 0.06 | 0.92 (0.85-1) | 1.21 (0.6-2.44) | 0.10 |
| rs519144 | 110986403 | G,A | 0.38 | 0.95 (0.93-0.97) | 6.43E-07 | 0.94 (0.92-0.97) | 0.91 (0.87-0.95) | 3.12E-06 |
| rs10979283 | 110987236 | C,A | 0.06 | 0.97 (0.93-1.01) | 0.12 | 0.96 (0.92-1.01) | 1 (0.79-1.27) | 0.26 |
| rs7043432 | 110988346 | A,G | 0.21 | 1.03 (1.01-1.06) | 8.78E-03 | 1.03 (1-1.06) | 1.08 (1.01-1.16) | 0.02 |
| rs837999 | 110989319 | C,A | 0.44 | 1.03 (1.01-1.05) | 1.96E-03 | 1.04 (1.01-1.07) | 1.06 (1.02-1.1) | 7.08E-03 |
| rs10979285 | 110989866 | G,A | 0.08 | 1.03 (1-1.07) | 0.06 | 1.05 (1.01-1.09) | 0.91 (0.77-1.08) | 0.02 |
| rs868895 | 110989880 | C,A | 0.38 | 0.95 (0.93-0.97) | 6.85E-07 | 0.94 (0.91-0.97) | 0.91 (0.87-0.95) | 3.13E-06 |
| rs1888913 | 110990323 | A,C | 0.18 | 1.03 (1-1.05) | 0.04 | 1.02 (0.99-1.05) | 1.06 (0.98-1.15) | 0.13 |
| rs111556338 | 110997417 | G,A | 0.02 | 0.95 (0.88-1.03) | 0.23 | 0.95 (0.87-1.03) | 1.22 (0.57-2.63) | 0.36 |
| rs75594954 | 110999079 | A,G | 0.17 | 1.03 (1-1.05) | 0.03 | 1.03 (1-1.06) | 1.06 (0.98-1.15) | 0.11 |
| rs35034713 | 111000704 | A,G | 0.05 | 0.97 (0.93-1.02) | 0.23 | 0.97 (0.92-1.01) | 1.05 (0.8-1.37) | 0.36 |
| rs7847854 | 111002172 | A,G | 0.37 | 0.95 (0.93-0.97) | 7.58E-07 | 0.94 (0.91-0.97) | 0.91 (0.87-0.95) | 2.78E-06 |
| rs74938381 | 111002999 | C,A | 0.04 | 1.02 (0.97-1.06) | 0.53 | 1.02 (0.97-1.07) | 0.96 (0.72-1.29) | 0.73 |
| rs1360533 | 111004738 | A,G | 0.40 | 0.95 (0.93-0.97) | 3.72E-07 | 0.93 (0.9-0.96) | 0.91 (0.88-0.95) | 4.77E-07 |
| rs1556472 | 111005974 | T,A | 0.07 | 0.98 (0.94-1.02) | 0.28 | 0.97 (0.94-1.01) | 1.02 (0.84-1.23) | 0.44 |
| rs4644328 | 111008699 | A,G | 0.33 | 0.95 (0.93-0.97) | 2.83E-06 | 0.95 (0.93-0.98) | 0.9 (0.86-0.95) | 1.68E-05 |
| rs4382542 | 111008909 | G,A | 0.33 | 0.95 (0.93-0.97) | 2.13E-06 | 0.95 (0.93-0.98) | 0.9 (0.86-0.95) | 1.27E-05 |
| rs1332299 | 111012416 | A,G | 0.25 | 1.01 (0.99-1.03) | 0.31 | 1.02 (0.99-1.05) | 1.01 (0.96-1.07) | 0.50 |
| rs1332300 | 111012582 | G,C | 0.34 | 0.95 (0.94-0.97) | 6.64E-06 | 0.96 (0.93-0.98) | 0.91 (0.87-0.95) | 3.81E-05 |
| rs10979291 | 111014868 | G,A | 0.07 | 0.98 (0.95-1.02) | 0.38 | 0.97 (0.94-1.01) | 1.09 (0.9-1.32) | 0.30 |
| rs1332302 | 111015811 | T,A | 0.49 | 1.03 (1.01-1.05) | 1.93E-03 | 1.05 (1.01-1.08) | 1.06 (1.02-1.1) | 3.83E-03 |
| rs7034951 | 111016395 | A,G | 0.33 | 0.95 (0.93-0.97) | 1.62E-06 | 0.95 (0.93-0.98) | 0.9 (0.86-0.95) | 9.95E-06 |
| rs1412433 | 111017507 | A,G | 0.18 | 1.02 (1-1.05) | 0.07 | 1.02 (0.99-1.06) | 1.04 (0.96-1.13) | 0.20 |
| rs2900448 | 111017606 | A,G | 0.01 | 1 (0.9-1.11) | 0.97 | #N/A | #N/A | #N/A |
| rs7043626 | 111018343 | A,C | 0.36 | 0.95 (0.93-0.97) | 7.22E-07 | 0.95 (0.92-0.98) | 0.9 (0.86-0.94) | 4.63E-06 |
| rs1537286 | 111021175 | G,A | 0.33 | 0.95 (0.93-0.97) | 1.48E-06 | 0.95 (0.93-0.98) | 0.9 (0.86-0.94) | 8.87E-06 |
| rs7030526 | 111022525 | A,G | 0.40 | 0.95 (0.93-0.97) | 4.31E-07 | 0.93 (0.91-0.96) | 0.91 (0.88-0.95) | 7.63E-07 |
| rs10739255 | 111023899 | G,A | 0.42 | 1.04 (1.02-1.06) | 1.75E-04 | 1.04 (1.01-1.07) | 1.08 (1.03-1.12) | 8.65E-04 |
| rs10739256 | 111024123 | A,G | 0.49 | 1.03 (1.01-1.05) | 1.56E-03 | 1.05 (1.02-1.09) | 1.06 (1.02-1.11) | 2.52E-03 |
| rs10759275 | 111024438 | A,C | 0.49 | 1.03 (1.01-1.05) | 1.47E-03 | 1.05 (1.02-1.09) | 1.06 (1.02-1.11) | 2.63E-03 |
| rs10979297 | 111024683 | T,A | 0.33 | 1.03 (1.01-1.05) | 2.80E-03 | 1.03 (1-1.06) | 1.06 (1.02-1.11) | 0.01 |
| rs1758617 | 111025649 | G,A | 0.42 | 1.04 (1.02-1.06) | 1.33E-04 | 1.04 (1.01-1.07) | 1.08 (1.04-1.12) | 6.65E-04 |
| rs2778252 | 111026158 | A,G | 0.50 | 0.97 (0.95-0.99) | 2.83E-03 | 0.99 (0.96-1.02) | 0.94 (0.91-0.98) | 4.96E-03 |
| rs13283710 | 111026582 | G,A | 0.07 | 0.98 (0.94-1.02) | 0.29 | 0.97 (0.93-1.01) | 1.07 (0.88-1.3) | 0.28 |
| rs3117888 | 111027827 | G,A | 0.49 | 1.03 (1.01-1.05) | 1.95E-03 | 1.05 (1.02-1.09) | 1.06 (1.02-1.1) | 2.80E-03 |
| rs2778253 | 111028359 | C,G | 0.42 | 1.04 (1.02-1.06) | 2.62E-04 | 1.04 (1.01-1.07) | 1.07 (1.03-1.12) | 1.25E-03 |
| rs2778254 | 111028362 | C,A | 0.42 | 1.04 (1.02-1.06) | 2.75E-04 | 1.04 (1.01-1.07) | 1.07 (1.03-1.12) | 1.31E-03 |
| rs3117889 | 111028604 | G,C | 0.42 | 1.04 (1.02-1.06) | 1.73E-04 | 1.04 (1.01-1.07) | 1.08 (1.04-1.12) | 8.65E-04 |
| rs3119743 | 111028887 | A,T | 0.42 | 1.04 (1.02-1.06) | 2.35E-04 | 1.04 (1.01-1.07) | 1.08 (1.03-1.12) | 1.15E-03 |
| rs1330591 | 111029103 | G,A | 0.48 | 1.03 (1.01-1.05) | 1.26E-03 | 1.05 (1.02-1.09) | 1.06 (1.02-1.11) | 1.53E-03 |
| rs2482603 | 111029969 | G,A | 0.50 | 1.03 (1.01-1.05) | 1.60E-03 | 1.05 (1.02-1.09) | 1.06 (1.02-1.11) | 2.31E-03 |
| rs3117890 | 111031148 | A,G | 0.49 | 1.03 (1.01-1.05) | 9.12E-04 | 1.05 (1.02-1.09) | 1.07 (1.03-1.11) | 1.85E-03 |
| rs645119 | 111031550 | G,A | 0.50 | 1.03 (1.01-1.05) | 9.37E-04 | 1.05 (1.02-1.09) | 1.07 (1.03-1.11) | 1.88E-03 |
| rs2778255 | 111032311 | G,A | 0.45 | 1.04 (1.02-1.06) | 5.14E-05 | 1.04 (1.01-1.07) | 1.08 (1.04-1.13) | 2.65E-04 |
| rs75484768 | 111034106 | C,A | 0.03 | 1.02 (0.97-1.08) | 0.42 | 1.02 (0.96-1.08) | 1.12 (0.76-1.66) | 0.68 |
| rs1323397 | 111034788 | A,G | 0.31 | 1.03 (1.01-1.05) | 2.39E-03 | 1.04 (1.01-1.07) | 1.06 (1.01-1.11) | 8.71E-03 |
| rs9299149 | 111034825 | C,A | 0.33 | 0.95 (0.93-0.97) | 6.39E-07 | 0.95 (0.92-0.98) | 0.9 (0.86-0.94) | 4.10E-06 |
| rs7039993 | 111034920 | G,C | 0.01 | 1.01 (0.93-1.09) | 0.89 | 1.02 (0.93-1.1) | 0.61 (0.27-1.36) | 0.45 |
| rs10759277 | 111035445 | A,G | 0.08 | 0.98 (0.95-1.02) | 0.36 | 0.98 (0.94-1.02) | 1.04 (0.88-1.22) | 0.44 |
| rs1575449 | 111037191 | A,G | 0.02 | 0.97 (0.9-1.05) | 0.47 | 0.97 (0.9-1.06) | 0.77 (0.34-1.75) | 0.68 |
| rs1323374 | 111037829 | T,A | 0.25 | 1.01 (0.99-1.03) | 0.40 | 1.01 (0.98-1.04) | 1.02 (0.96-1.08) | 0.68 |
| rs117563258 | 111038705 | G,A | 0.02 | 1 (0.93-1.08) | 0.98 | 1.01 (0.93-1.09) | 0.62 (0.26-1.49) | 0.55 |
| rs10979300 | 111040235 | G,A | 0.09 | 1.03 (1-1.07) | 0.09 | 1.04 (1-1.08) | 0.94 (0.8-1.11) | 0.07 |
| rs1323376 | 111042771 | G,A | 0.33 | 0.95 (0.93-0.97) | 6.36E-07 | 0.95 (0.92-0.98) | 0.9 (0.86-0.94) | 4.12E-06 |
| rs1323377 | 111043009 | A,G | 0.33 | 0.95 (0.93-0.97) | 7.59E-07 | 0.95 (0.92-0.98) | 0.9 (0.86-0.94) | 4.88E-06 |
| rs10979301 | 111044933 | A,G | 0.34 | 0.95 (0.93-0.97) | 1.21E-06 | 0.95 (0.92-0.98) | 0.9 (0.86-0.95) | 7.66E-06 |
| rs12552629 | 111047312 | A,G | 0.47 | 0.96 (0.94-0.98) | 1.40E-04 | 0.97 (0.94-1) | 0.93 (0.89-0.96) | 5.73E-04 |
| rs7852572 | 111047463 | G,A | 0.36 | 1.04 (1.02-1.06) | 9.40E-05 | 1.04 (1.01-1.08) | 1.08 (1.03-1.13) | 4.66E-04 |
| rs72747953 | 111050043 | G,A | 0.05 | 1.03 (0.99-1.08) | 0.15 | 1.04 (0.99-1.09) | 0.95 (0.73-1.23) | 0.22 |
| rs7021201 | 111050366 | G,A | 0.48 | 0.96 (0.94-0.98) | 1.22E-04 | 0.98 (0.94-1.01) | 0.93 (0.89-0.96) | 4.06E-04 |
| rs10979302 | 111050704 | A,C | 0.07 | 0.98 (0.95-1.02) | 0.35 | 0.97 (0.93-1.01) | 1.1 (0.9-1.33) | 0.25 |
| rs12353336 | 111050810 | G,A | 0.31 | 0.96 (0.94-0.98) | 1.70E-05 | 0.96 (0.93-0.99) | 0.91 (0.87-0.95) | 9.22E-05 |
| rs117088293 | 111051035 | G,A | 0.03 | 1.03 (0.97-1.08) | 0.36 | 1.02 (0.97-1.08) | 1.11 (0.76-1.63) | 0.63 |
| rs10117055 | 111051418 | G,A | 0.45 | 1.04 (1.02-1.06) | 2.50E-05 | 1.04 (1-1.07) | 1.09 (1.05-1.13) | 1.26E-04 |
| rs10114572 | 111051611 | A,G | 0.45 | 1.04 (1.02-1.06) | 5.55E-05 | 1.04 (1-1.07) | 1.08 (1.04-1.13) | 2.75E-04 |
| rs10117687 | 111051626 | T,A | 0.31 | 0.96 (0.94-0.98) | 3.10E-05 | 0.96 (0.93-0.99) | 0.91 (0.87-0.96) | 1.64E-04 |
| rs73653749 | 111051751 | A,G | 0.03 | 0.95 (0.9-1) | 0.04 | 0.94 (0.89-0.99) | 1.05 (0.71-1.56) | 0.09 |
| rs4978678 | 111053537 | A,C | 0.34 | 0.95 (0.93-0.97) | 4.22E-07 | 0.95 (0.92-0.98) | 0.9 (0.86-0.94) | 2.76E-06 |
| rs56125520 | 111054295 | A,C | 0.16 | 1.01 (0.98-1.04) | 0.43 | 1.01 (0.98-1.05) | 1 (0.92-1.09) | 0.65 |
| rs7046291 | 111054302 | G,C | 0.23 | 1 (0.98-1.02) | 0.91 | 1 (0.98-1.03) | 0.99 (0.93-1.05) | 0.84 |
| rs10979304 | 111055670 | A,G | 0.07 | 0.98 (0.94-1.02) | 0.26 | 0.97 (0.93-1.01) | 1.05 (0.86-1.29) | 0.32 |
| rs55896475 | 111056782 | G,A | 0.14 | 1.02 (1-1.05) | 0.09 | 1.03 (0.99-1.06) | 1.04 (0.94-1.15) | 0.22 |
| rs76429636 | 111057036 | G,A | 0.02 | 0.85 (0.8-0.91) | 4.56E-07 | 0.86 (0.8-0.91) | 0.62 (0.37-1.05) | 2.49E-06 |
| rs13300950 | 111057524 | A,G | 0.07 | 0.98 (0.95-1.02) | 0.41 | 0.98 (0.94-1.02) | 1.07 (0.88-1.31) | 0.39 |
| rs77922938 | 111060196 | G,A | 0.06 | 1.05 (1.01-1.09) | 0.02 | 1.04 (0.99-1.09) | 1.29 (1.01-1.64) | 0.03 |
| rs1323380 | 111060921 | C,A | 0.16 | 1.01 (0.98-1.04) | 0.44 | 1.01 (0.98-1.05) | 1 (0.92-1.09) | 0.64 |
| rs1885973 | 111061706 | T,A | 0.23 | 1 (0.98-1.02) | 1.00 | 1.01 (0.98-1.04) | 0.97 (0.91-1.03) | 0.37 |
| rs117191246 | 111061960 | A,G | 0.01 | 0.93 (0.84-1.03) | 0.15 | #N/A | #N/A | #N/A |
| rs10979312 | 111064186 | A,C | 0.34 | 0.95 (0.93-0.97) | 1.02E-06 | 0.95 (0.92-0.98) | 0.9 (0.86-0.95) | 6.51E-06 |
| rs1323381 | 111064438 | G,A | 0.14 | 1.02 (0.99-1.05) | 0.13 | 1.03 (0.99-1.06) | 1.02 (0.93-1.13) | 0.30 |
| rs1407849 | 111064598 | A,T | 0.21 | 1.01 (0.98-1.03) | 0.51 | 1.01 (0.98-1.04) | 1 (0.94-1.07) | 0.68 |
| rs1407850 | 111064801 | A,G | 0.45 | 1.04 (1.02-1.06) | 3.19E-05 | 1.03 (1-1.07) | 1.09 (1.05-1.13) | 1.51E-04 |
| rs1323383 | 111065424 | A,G | 0.45 | 1.04 (1.02-1.06) | 4.76E-05 | 1.03 (1-1.07) | 1.09 (1.04-1.13) | 2.12E-04 |
| rs117936512 | 111066476 | C,A | 0.45 | 1.04 (1.02-1.06) | 2.01E-05 | 1.03 (1-1.07) | 1.09 (1.05-1.13) | 8.48E-05 |
| rs17543333 | 111066833 | A,C | 0.16 | 1.01 (0.98-1.03) | 0.65 | 1.01 (0.98-1.04) | 0.99 (0.91-1.08) | 0.78 |
| rs16912868 | 111067924 | A,G | 0.24 | 1 (0.98-1.02) | 0.86 | 1.01 (0.98-1.04) | 0.97 (0.91-1.03) | 0.47 |
| rs116991065 | 111070042 | A,G | 0.14 | 1.02 (0.99-1.05) | 0.16 | 1.02 (0.99-1.05) | 1.03 (0.94-1.14) | 0.36 |
| rs2093680 | 111070724 | G,A | 0.49 | 0.96 (0.95-0.98) | 1.81E-04 | 0.97 (0.94-1.01) | 0.93 (0.89-0.97) | 7.11E-04 |
| rs79831337 | 111070857 | A,G | 0.37 | 1.04 (1.02-1.06) | 6.96E-05 | 1.05 (1.02-1.08) | 1.08 (1.03-1.13) | 3.00E-04 |
| rs1407851 | 111071459 | G,C | 0.45 | 1.04 (1.02-1.06) | 1.87E-04 | 1.02 (0.99-1.05) | 1.08 (1.04-1.12) | 4.83E-04 |
| rs56020002 | 111071733 | G,A | 0.14 | 1.02 (0.99-1.05) | 0.17 | 1.02 (0.99-1.05) | 1.04 (0.94-1.15) | 0.40 |
| rs10979314 | 111072429 | G,A | 0.08 | 1 (0.97-1.04) | 0.86 | 1.02 (0.98-1.06) | 0.85 (0.71-1.02) | 0.13 |
| rs10979315 | 111072586 | A,G | 0.48 | 0.97 (0.95-0.99) | 5.56E-04 | 0.97 (0.94-1) | 0.93 (0.9-0.97) | 2.33E-03 |
| rs12349823 | 111072668 | G,A | 0.48 | 0.97 (0.95-0.99) | 5.78E-04 | 0.97 (0.94-1) | 0.93 (0.9-0.97) | 2.43E-03 |
| rs12336489 | 111073103 | A,G | 0.34 | 0.95 (0.93-0.97) | 1.85E-06 | 0.95 (0.92-0.98) | 0.91 (0.87-0.95) | 1.15E-05 |
| rs12336573 | 111073347 | A,G | 0.34 | 0.95 (0.93-0.97) | 1.50E-06 | 0.95 (0.92-0.98) | 0.91 (0.87-0.95) | 9.38E-06 |
| rs10283642 | 111074243 | G,A | 0.47 | 0.98 (0.96-1) | 0.02 | 0.99 (0.96-1.02) | 0.95 (0.92-0.99) | 0.05 |
| rs10739257 | 111074457 | C,A | 0.30 | 0.97 (0.95-0.99) | 0.01 | 0.97 (0.95-1) | 0.95 (0.9-1) | 0.05 |
| rs62570194 | 111075953 | A,G | 0.10 | 1.01 (0.98-1.04) | 0.57 | 1 (0.97-1.04) | 1.07 (0.93-1.23) | 0.60 |
| rs7033858 | 111077461 | A,G | 0.36 | 1.01 (0.99-1.03) | 0.59 | 1.01 (0.98-1.04) | 1.01 (0.97-1.05) | 0.82 |
| rs1923964 | 111081694 | C,G | 0.17 | 0.99 (0.97-1.02) | 0.65 | 1 (0.97-1.04) | 0.95 (0.88-1.03) | 0.41 |
| rs4978345 | 111082271 | A,C | 0.35 | 1.01 (0.99-1.03) | 0.32 | 1.01 (0.98-1.04) | 1.02 (0.98-1.07) | 0.60 |
| rs74793433 | 111085785 | G,A | 0.03 | 1.04 (0.98-1.09) | 0.18 | 1.02 (0.97-1.08) | 1.51 (1-2.29) | 0.10 |
| rs62570196 | 111086170 | A,G | 0.07 | 0.99 (0.96-1.03) | 0.62 | 0.99 (0.95-1.03) | 0.97 (0.81-1.15) | 0.87 |
| rs62570197 | 111086510 | G,A | 0.10 | 1.01 (0.98-1.04) | 0.44 | 1 (0.97-1.04) | 1.11 (0.97-1.26) | 0.32 |
| rs77284504 | 111086758 | A,T | 0.06 | 0.98 (0.94-1.02) | 0.33 | 0.98 (0.94-1.02) | 0.96 (0.77-1.19) | 0.62 |
| rs10816659 | 111092798 | G,A | 0.21 | 1.02 (1-1.05) | 0.06 | 1.03 (1-1.06) | 1.04 (0.97-1.11) | 0.17 |
| rs62570199 | 111094828 | G,A | 0.02 | 0.98 (0.93-1.05) | 0.62 | 0.97 (0.91-1.03) | 1.98 (1.14-3.44) | 0.03 |
| rs2417854 | 111095847 | C,G | 0.20 | 1.03 (1-1.05) | 0.04 | 1.03 (1-1.06) | 1.04 (0.97-1.12) | 0.12 |
| rs1323389 | 111098510 | A,G | 0.21 | 1.02 (1-1.05) | 0.07 | 1.02 (0.99-1.05) | 1.04 (0.98-1.11) | 0.18 |
| rs16936302 | 111100826 | A,G | 0.24 | 1 (0.98-1.02) | 0.91 | 1 (0.97-1.03) | 0.99 (0.93-1.05) | 0.89 |

**Supplemental Table 2B. Association statistics and effect estimates for 9q31.2 fine-mapping SNPs in breast cancer cases and controls with Asian ancestry.**

| SNP | Location | Alleles | MAF | Trend OR | P-trend | Het OR | Hom OR | P-geno |
| --- | --- | --- | --- | --- | --- | --- | --- | --- |
| rs2900441 | 110740582 | T,A | 0.07 | 0.96 (0.87-1.07) | 0.45 | 0.96 (0.86-1.08) | 0.92 (0.61-1.39) | 0.75 |
| rs837410 | 110740832 | A,G | 0.43 | 1.07 (1.02-1.13) | 0.01 | 1.07 (0.99-1.17) | 1.14 (1.03-1.27) | 0.04 |
| rs629480 | 110741144 | A,G | 0.21 | 1.01 (0.95-1.07) | 0.81 | 1.03 (0.95-1.11) | 0.96 (0.8-1.15) | 0.68 |
| rs657659 | 110742851 | A,G | 0.33 | 1.08 (1.02-1.14) | 0.01 | 1.07 (0.99-1.16) | 1.16 (1.03-1.31) | 0.03 |
| rs7024363 | 110746405 | A,C | 0.10 | 1 (0.91-1.09) | 0.94 | 1.04 (0.94-1.15) | 0.77 (0.56-1.06) | 0.15 |
| rs1556939 | 110749585 | C,G | 0.47 | 0.93 (0.88-0.98) | 4.69E-03 | 0.89 (0.81-0.97) | 0.87 (0.78-0.96) | 8.47E-03 |
| rs7029391 | 110751144 | A,G | 0.47 | 0.92 (0.87-0.97) | 2.00E-03 | 0.89 (0.82-0.97) | 0.85 (0.77-0.95) | 5.58E-03 |
| rs480113 | 110751682 | G,A | 0.04 | 1.16 (1.02-1.31) | 0.02 | 1.17 (1.03-1.34) | 1.06 (0.49-2.29) | 0.06 |
| rs1009813 | 110752106 | G,A | 0.07 | 0.94 (0.85-1.05) | 0.28 | 0.95 (0.85-1.07) | 0.84 (0.55-1.29) | 0.54 |
| rs7851574 | 110753079 | A,G | 0.44 | 0.92 (0.87-0.97) | 2.08E-03 | 0.89 (0.82-0.97) | 0.86 (0.77-0.95) | 5.14E-03 |
| rs1934419 | 110754102 | A,G | 0.44 | 0.92 (0.88-0.97) | 2.86E-03 | 0.89 (0.82-0.97) | 0.86 (0.77-0.96) | 7.07E-03 |
| rs1769652 | 110754778 | A,G | 0.24 | 1 (0.94-1.06) | 0.96 | 1.02 (0.94-1.1) | 0.95 (0.81-1.11) | 0.65 |
| rs10979155 | 110754824 | A,G | 0.04 | 0.91 (0.8-1.04) | 0.18 | 0.91 (0.79-1.04) | 0.99 (0.41-2.42) | 0.37 |
| rs668474 | 110755943 | G,A | 0.09 | 1.08 (0.98-1.18) | 0.11 | 1.07 (0.96-1.18) | 1.29 (0.84-1.97) | 0.25 |
| rs10979157 | 110757887 | G,A | 0.41 | 0.95 (0.91-1.01) | 0.08 | 0.94 (0.87-1.02) | 0.92 (0.82-1.02) | 0.20 |
| rs13288365 | 110759721 | G,C | 0.15 | 1.01 (0.94-1.08) | 0.84 | 0.99 (0.91-1.08) | 1.09 (0.86-1.37) | 0.75 |
| rs673985 | 110759922 | G,A | 0.31 | 1.08 (1.02-1.14) | 6.89E-03 | 1.1 (1.02-1.19) | 1.14 (1.01-1.29) | 0.02 |
| rs10979158 | 110760000 | A,G | 0.35 | 1.07 (1.02-1.13) | 0.01 | 1.08 (1-1.17) | 1.15 (1.02-1.29) | 0.04 |
| rs4979617 | 110760144 | G,A | 0.35 | 1.07 (1.02-1.13) | 0.01 | 1.07 (0.99-1.16) | 1.15 (1.03-1.3) | 0.03 |
| rs4979618 | 110760316 | A,G | 0.36 | 1.07 (1.02-1.13) | 0.01 | 1.07 (0.99-1.16) | 1.16 (1.03-1.3) | 0.03 |
| rs4979620 | 110760910 | C,G | 0.47 | 1.06 (1-1.11) | 0.03 | 1.05 (0.96-1.15) | 1.12 (1.01-1.24) | 0.10 |
| rs12342578 | 110761002 | G,A | 0.38 | 0.95 (0.91-1.01) | 0.09 | 0.93 (0.86-1.01) | 0.92 (0.83-1.03) | 0.17 |
| rs12342611 | 110761104 | C,G | 0.34 | 1.08 (1.02-1.14) | 6.81E-03 | 1.07 (0.99-1.16) | 1.17 (1.04-1.32) | 0.02 |
| rs12347482 | 110761427 | C,A | 0.46 | 1.05 (1-1.11) | 0.05 | 1.05 (0.96-1.14) | 1.11 (1-1.23) | 0.15 |
| rs12236795 | 110761513 | A,G | 0.46 | 1.05 (1-1.11) | 0.05 | 1.05 (0.96-1.14) | 1.11 (1-1.23) | 0.14 |
| rs4979621 | 110764599 | A,G | 0.46 | 1.05 (1-1.1) | 0.08 | 1.04 (0.96-1.14) | 1.1 (0.99-1.22) | 0.21 |
| rs4979622 | 110764799 | G,A | 0.44 | 1.06 (1-1.11) | 0.03 | 1.05 (0.96-1.14) | 1.12 (1.01-1.25) | 0.10 |
| rs4978665 | 110764844 | G,C | 0.09 | 1.04 (0.95-1.14) | 0.41 | 1.07 (0.97-1.19) | 0.79 (0.51-1.21) | 0.19 |
| rs10759270 | 110764981 | G,A | 0.50 | 0.95 (0.9-1) | 0.05 | 0.94 (0.86-1.03) | 0.9 (0.81-1) | 0.13 |
| rs12554275 | 110765067 | C,G | 0.12 | 0.97 (0.9-1.05) | 0.50 | 0.96 (0.88-1.05) | 1.01 (0.75-1.37) | 0.70 |
| rs10512369 | 110765359 | G,A | 0.06 | 0.96 (0.86-1.08) | 0.50 | 0.98 (0.87-1.1) | 0.82 (0.51-1.31) | 0.67 |
| rs1416836 | 110766963 | G,A | 0.14 | 1 (0.93-1.08) | 0.91 | 1 (0.91-1.09) | 1.05 (0.82-1.34) | 0.93 |
| rs9299144 | 110767478 | G,A | 0.38 | 1.05 (1-1.11) | 0.07 | 1.06 (0.98-1.15) | 1.09 (0.98-1.22) | 0.19 |
| rs10120885 | 110768664 | C,G | 0.35 | 1.07 (1.01-1.13) | 0.02 | 1.09 (1.01-1.18) | 1.12 (0.99-1.26) | 0.05 |
| rs4979623 | 110769283 | A,G | 0.39 | 1.04 (0.99-1.1) | 0.12 | 1.06 (0.98-1.15) | 1.08 (0.96-1.2) | 0.25 |
| rs9695201 | 110769468 | G,A | 0.38 | 1.04 (0.99-1.1) | 0.13 | 1.06 (0.97-1.14) | 1.08 (0.96-1.21) | 0.28 |
| rs7860780 | 110772665 | G,A | 0.38 | 1.05 (1-1.11) | 0.08 | 1.06 (0.98-1.15) | 1.09 (0.98-1.22) | 0.19 |
| rs9695286 | 110773067 | G,A | 0.35 | 1.07 (1.01-1.12) | 0.02 | 1.09 (1-1.18) | 1.12 (0.99-1.26) | 0.06 |
| rs1339756 | 110776765 | G,A | 0.38 | 1.04 (0.99-1.1) | 0.12 | 1.06 (0.98-1.15) | 1.08 (0.97-1.21) | 0.26 |
| rs10979163 | 110780615 | G,A | 0.13 | 1.03 (0.95-1.11) | 0.49 | 1.03 (0.94-1.12) | 1.07 (0.82-1.41) | 0.78 |
| rs7029094 | 110782565 | G,A | 0.49 | 1.04 (0.99-1.1) | 0.10 | 1.05 (0.96-1.15) | 1.09 (0.98-1.21) | 0.25 |
| rs10512368 | 110783036 | A,G | 0.39 | 1.05 (0.99-1.1) | 0.09 | 1.07 (0.99-1.16) | 1.08 (0.97-1.21) | 0.18 |
| rs10816612 | 110786222 | A,G | 0.39 | 1.05 (0.99-1.1) | 0.10 | 1.07 (0.99-1.16) | 1.08 (0.97-1.21) | 0.21 |
| rs4979625 | 110786533 | T,A | 0.39 | 1.05 (0.99-1.1) | 0.09 | 1.07 (0.99-1.16) | 1.08 (0.97-1.21) | 0.19 |
| rs7868720 | 110787096 | A,G | 0.39 | 1.05 (0.99-1.1) | 0.09 | 1.07 (0.99-1.16) | 1.08 (0.97-1.21) | 0.19 |
| rs1995761 | 110795237 | G,A | 0.38 | 1.04 (0.99-1.1) | 0.11 | 1.06 (0.98-1.15) | 1.08 (0.96-1.2) | 0.23 |
| rs10759272 | 110795806 | G,C | 0.39 | 1.05 (0.99-1.1) | 0.10 | 1.07 (0.99-1.16) | 1.08 (0.96-1.2) | 0.17 |
| rs10979179 | 110805072 | G,A | 0.36 | 0.93 (0.89-0.99) | 0.01 | 0.91 (0.84-0.98) | 0.9 (0.8-1.01) | 0.03 |
| rs7020797 | 110806917 | G,A | 0.49 | 0.95 (0.9-1) | 0.07 | 0.96 (0.88-1.05) | 0.91 (0.82-1.01) | 0.17 |
| rs10816621 | 110808571 | G,A | 0.08 | 0.98 (0.9-1.08) | 0.75 | 0.99 (0.89-1.09) | 0.96 (0.63-1.47) | 0.95 |
| rs13301510 | 110809708 | G,A | 0.12 | 1.01 (0.94-1.1) | 0.72 | 1.02 (0.93-1.12) | 0.98 (0.74-1.3) | 0.87 |
| rs6477612 | 110811552 | A,G | 0.41 | 1.06 (1.01-1.12) | 0.02 | 1.09 (1.01-1.18) | 1.12 (1.01-1.25) | 0.05 |
| rs6477613 | 110811614 | G,A | 0.49 | 0.95 (0.9-1) | 0.04 | 0.96 (0.88-1.05) | 0.9 (0.81-0.99) | 0.11 |
| rs1318148 | 110814693 | G,C | 0.49 | 0.95 (0.9-1) | 0.06 | 0.97 (0.89-1.06) | 0.9 (0.82-1) | 0.15 |
| rs892687 | 110814936 | C,A | 0.41 | 1.06 (1.01-1.12) | 0.02 | 1.08 (0.99-1.17) | 1.12 (1.01-1.25) | 0.07 |
| rs7861155 | 110815564 | G,C | 0.07 | 1.05 (0.95-1.17) | 0.34 | 1.05 (0.94-1.17) | 1.15 (0.66-2.01) | 0.63 |
| rs10979182 | 110817020 | G,A | 0.40 | 1.06 (1-1.11) | 0.04 | 1.07 (0.98-1.16) | 1.11 (1-1.24) | 0.12 |
| rs4978668 | 110817549 | A,C | 0.41 | 1.05 (1-1.11) | 0.05 | 1.06 (0.98-1.15) | 1.11 (0.99-1.23) | 0.14 |
| rs10979183 | 110821050 | A,G | 0.41 | 1.06 (1.01-1.12) | 0.03 | 1.06 (0.98-1.15) | 1.13 (1.01-1.25) | 0.09 |
| rs1878600 | 110821830 | G,A | 0.13 | 1 (0.93-1.08) | 0.90 | 1 (0.92-1.1) | 1.02 (0.78-1.33) | 0.99 |
| rs1434836 | 110822658 | G,A | 0.40 | 1.05 (1-1.11) | 0.05 | 1.07 (0.98-1.16) | 1.11 (0.99-1.23) | 0.14 |
| rs7357711 | 110823759 | A,G | 0.14 | 1.02 (0.95-1.1) | 0.57 | 1.02 (0.94-1.12) | 1.05 (0.8-1.36) | 0.85 |
| rs10979184 | 110825659 | A,G | 0.37 | 0.95 (0.9-1) | 0.04 | 0.92 (0.85-1) | 0.91 (0.81-1.02) | 0.09 |
| rs34528190 | 110825688 | A,T | 0.06 | 0.97 (0.87-1.08) | 0.54 | 0.97 (0.86-1.09) | 0.91 (0.55-1.5) | 0.83 |
| rs4978670 | 110826546 | G,A | 0.41 | 1.06 (1.01-1.12) | 0.02 | 1.07 (0.99-1.16) | 1.12 (1.01-1.25) | 0.08 |
| rs10816623 | 110828308 | A,G | 0.38 | 0.95 (0.9-1) | 0.05 | 0.92 (0.85-1) | 0.92 (0.82-1.03) | 0.10 |
| rs13289116 | 110829012 | A,G | 0.13 | 1 (0.93-1.08) | 0.99 | 1.01 (0.92-1.1) | 0.97 (0.74-1.27) | 0.97 |
| rs13291052 | 110832584 | A,G | 0.16 | 0.99 (0.92-1.07) | 0.83 | 0.99 (0.91-1.07) | 1 (0.8-1.26) | 0.96 |
| rs61538103 | 110833526 | C,A | 0.12 | 0.99 (0.91-1.07) | 0.82 | 1 (0.91-1.09) | 0.94 (0.7-1.27) | 0.92 |
| rs10481656 | 110836284 | A,G | 0.42 | 1.06 (1.01-1.12) | 0.03 | 1.1 (1.01-1.19) | 1.11 (1-1.24) | 0.05 |
| rs10481657 | 110836385 | G,A | 0.14 | 0.99 (0.92-1.07) | 0.78 | 1 (0.92-1.09) | 0.94 (0.74-1.21) | 0.90 |
| rs10481658 | 110836416 | G,A | 0.47 | 0.93 (0.88-0.98) | 6.44E-03 | 0.95 (0.88-1.04) | 0.86 (0.78-0.96) | 0.02 |
| **rs10816625** | **110837073** | **A,G** | **0.40** | **1.12 (1.06-1.18)** | **2.77E-05** | **1.12 (1.03-1.21)** | **1.26 (1.13-1.4)** | **1.52E-04** |
| **rs13294895** | **110837176** | **G,A** | **0.03** | **1.04 (0.89-1.21)** | **0.66** | **1 (0.85-1.18)** | **2.03 (0.77-5.31)** | **0.35** |
| rs35909461 | 110838269 | A,G | 0.23 | 0.95 (0.89-1.01) | 0.09 | 0.92 (0.85-1) | 0.96 (0.81-1.14) | 0.13 |
| rs7042321 | 110841593 | G,A | 0.24 | 0.97 (0.92-1.04) | 0.41 | 0.98 (0.9-1.05) | 0.95 (0.81-1.12) | 0.71 |
| rs10118883 | 110843927 | A,G | 0.03 | 1.1 (0.94-1.28) | 0.25 | 1.08 (0.92-1.28) | 1.47 (0.57-3.75) | 0.47 |
| rs10979190 | 110845216 | G,A | 0.04 | 0.87 (0.76-0.99) | 0.04 | 0.86 (0.75-0.99) | 0.9 (0.45-1.81) | 0.10 |
| rs10979202 | 110849566 | A,C | 0.05 | 0.91 (0.81-1.03) | 0.14 | 0.9 (0.8-1.03) | 0.97 (0.51-1.84) | 0.31 |
| rs10979204 | 110850362 | G,C | 0.05 | 0.91 (0.8-1.02) | 0.10 | 0.9 (0.79-1.03) | 0.87 (0.47-1.6) | 0.26 |
| rs12236285 | 110855243 | G,A | 0.05 | 0.83 (0.74-0.94) | 3.95E-03 | 0.83 (0.73-0.94) | 0.77 (0.4-1.47) | 0.01 |
| rs1434851 | 110857960 | A,G | 0.31 | 0.97 (0.92-1.03) | 0.32 | 0.98 (0.91-1.06) | 0.94 (0.82-1.07) | 0.59 |
| rs1813421 | 110858823 | G,A | 0.32 | 0.95 (0.9-1) | 0.07 | 0.93 (0.86-1) | 0.93 (0.81-1.05) | 0.13 |
| rs7869753 | 110864852 | C,A | 0.32 | 0.94 (0.89-0.99) | 0.03 | 0.92 (0.85-0.99) | 0.91 (0.8-1.03) | 0.07 |
| rs78833899 | 110866666 | A,G | 0.05 | 0.88 (0.78-0.98) | 0.02 | 0.87 (0.77-0.99) | 0.78 (0.42-1.45) | 0.08 |
| rs78417418 | 110871005 | A,G | 0.05 | 0.88 (0.78-1) | 0.04 | 0.88 (0.77-1) | 0.82 (0.41-1.65) | 0.12 |
| rs10979214 | 110871414 | G,A | 0.08 | 0.91 (0.82-1) | 0.06 | 0.9 (0.8-1) | 0.88 (0.61-1.28) | 0.15 |
| rs7042864 | 110872307 | G,A | 0.08 | 0.92 (0.84-1.02) | 0.13 | 0.92 (0.82-1.03) | 0.89 (0.61-1.3) | 0.31 |
| rs10124038 | 110872527 | G,A | 0.30 | 0.97 (0.91-1.03) | 0.28 | 0.96 (0.89-1.04) | 0.95 (0.83-1.09) | 0.51 |
| rs2043486 | 110873028 | A,G | 0.03 | 1.02 (0.88-1.19) | 0.79 | 1.03 (0.87-1.21) | 0.97 (0.41-2.29) | 0.95 |
| rs62569845 | 110874785 | A,G | 0.13 | 0.97 (0.89-1.05) | 0.39 | 0.97 (0.89-1.06) | 0.92 (0.67-1.26) | 0.68 |
| rs10979217 | 110875895 | A,G | 0.09 | 1.02 (0.93-1.11) | 0.68 | 1 (0.91-1.11) | 1.17 (0.8-1.71) | 0.72 |
| rs1999456 | 110877304 | C,G | 0.29 | 0.96 (0.91-1.02) | 0.18 | 0.93 (0.86-1.01) | 0.96 (0.84-1.11) | 0.22 |
| rs1999457 | 110877440 | T,A | 0.29 | 0.96 (0.91-1.02) | 0.18 | 0.93 (0.86-1.01) | 0.96 (0.84-1.1) | 0.23 |
| rs837983 | 110881453 | A,G | 0.04 | 0.92 (0.81-1.04) | 0.19 | 0.95 (0.83-1.08) | 0.51 (0.23-1.14) | 0.17 |
| rs10979219 | 110881731 | A,G | 0.03 | 0.95 (0.81-1.11) | 0.52 | 0.99 (0.83-1.17) | 0.57 (0.26-1.25) | 0.35 |
| rs667052 | 110882390 | G,A | 0.20 | 0.95 (0.89-1.02) | 0.15 | 0.97 (0.89-1.05) | 0.87 (0.72-1.05) | 0.29 |
| rs680138 | 110882980 | A,G | 0.04 | 0.91 (0.8-1.03) | 0.13 | 0.94 (0.82-1.07) | 0.45 (0.2-1.04) | 0.10 |
| rs12376798 | 110883675 | G,A | 0.03 | 0.92 (0.78-1.09) | 0.35 | 0.96 (0.81-1.15) | 0.52 (0.23-1.2) | 0.27 |
| rs630965 | 110885479 | A,G | 0.07 | 0.94 (0.85-1.04) | 0.22 | 0.95 (0.85-1.05) | 0.81 (0.49-1.36) | 0.45 |
| rs631475 | 110885650 | A,C | 0.07 | 0.94 (0.85-1.04) | 0.25 | 0.95 (0.85-1.06) | 0.81 (0.49-1.36) | 0.48 |
| rs497006 | 110885781 | A,G | 0.07 | 0.94 (0.85-1.04) | 0.21 | 0.94 (0.85-1.05) | 0.81 (0.49-1.36) | 0.43 |
| rs519679 | 110885947 | G,C | 0.07 | 0.94 (0.85-1.04) | 0.24 | 0.95 (0.85-1.06) | 0.81 (0.48-1.37) | 0.47 |
| rs520613 | 110886052 | A,G | 0.07 | 0.96 (0.87-1.06) | 0.42 | 0.97 (0.87-1.08) | 0.83 (0.5-1.39) | 0.66 |
| rs522463 | 110886254 | A,C | 0.07 | 0.94 (0.85-1.04) | 0.20 | 0.95 (0.85-1.05) | 0.78 (0.46-1.32) | 0.40 |
| rs12380317 | 110886306 | T,A | 0.03 | 0.93 (0.79-1.1) | 0.39 | 0.96 (0.81-1.15) | 0.59 (0.27-1.32) | 0.39 |
| rs525142 | 110886534 | A,G | 0.07 | 0.93 (0.84-1.03) | 0.15 | 0.94 (0.84-1.04) | 0.78 (0.46-1.31) | 0.33 |
| rs62569853 | 110886727 | A,G | 0.11 | 0.98 (0.9-1.06) | 0.57 | 1 (0.91-1.1) | 0.8 (0.56-1.13) | 0.45 |
| rs527071 | 110886745 | A,C | 0.07 | 0.93 (0.84-1.03) | 0.17 | 0.94 (0.84-1.05) | 0.78 (0.46-1.32) | 0.35 |
| rs548980 | 110886840 | A,G | 0.04 | 0.91 (0.8-1.03) | 0.14 | 0.93 (0.81-1.06) | 0.56 (0.26-1.21) | 0.19 |
| rs648354 | 110887106 | A,G | 0.07 | 0.93 (0.84-1.03) | 0.16 | 0.94 (0.84-1.05) | 0.78 (0.46-1.32) | 0.35 |
| rs1025573 | 110887338 | G,A | 0.10 | 0.97 (0.89-1.06) | 0.49 | 0.99 (0.91-1.09) | 0.78 (0.55-1.11) | 0.39 |
| rs580018 | 110887934 | A,G | 0.03 | 0.9 (0.76-1.06) | 0.20 | 0.9 (0.76-1.06) | 0.83 (0.26-2.66) | 0.43 |
| rs471467 | 110888113 | A,G | 0.07 | 0.93 (0.84-1.03) | 0.14 | 0.94 (0.84-1.05) | 0.72 (0.42-1.23) | 0.26 |
| rs472483 | 110888260 | G,A | 0.07 | 0.93 (0.84-1.03) | 0.15 | 0.94 (0.85-1.05) | 0.72 (0.42-1.23) | 0.27 |
| rs865686 | 110888478 | A,C | 0.07 | 0.93 (0.84-1.02) | 0.13 | 0.94 (0.84-1.04) | 0.75 (0.44-1.27) | 0.28 |
| rs7026944 | 110891253 | A,G | 0.03 | 0.94 (0.8-1.11) | 0.47 | 0.99 (0.83-1.17) | 0.52 (0.23-1.2) | 0.29 |
| rs7870781 | 110891494 | A,G | 0.08 | 0.97 (0.88-1.07) | 0.57 | 0.98 (0.88-1.09) | 0.9 (0.62-1.32) | 0.81 |
| rs6477617 | 110892030 | G,C | 0.08 | 0.96 (0.87-1.05) | 0.36 | 0.97 (0.87-1.08) | 0.81 (0.55-1.2) | 0.52 |
| rs510294 | 110892787 | A,T | 0.04 | 0.93 (0.82-1.05) | 0.25 | 0.95 (0.83-1.09) | 0.55 (0.25-1.22) | 0.25 |
| rs7862747 | 110892899 | A,C | 0.04 | 0.93 (0.82-1.05) | 0.24 | 0.95 (0.83-1.08) | 0.58 (0.27-1.26) | 0.28 |
| rs628931 | 110893030 | G,A | 0.04 | 0.92 (0.81-1.04) | 0.18 | 0.95 (0.83-1.09) | 0.53 (0.26-1.08) | 0.15 |
| rs10979226 | 110893793 | A,T | 0.04 | 0.99 (0.87-1.14) | 0.94 | 1.02 (0.88-1.18) | 0.66 (0.31-1.4) | 0.51 |
| rs10979227 | 110893829 | A,G | 0.04 | 0.99 (0.86-1.14) | 0.91 | 1.02 (0.88-1.18) | 0.66 (0.31-1.4) | 0.52 |
| rs659713 | 110893949 | C,A | 0.04 | 0.92 (0.81-1.05) | 0.20 | 0.94 (0.82-1.08) | 0.58 (0.27-1.27) | 0.26 |
| rs34137313 | 110893956 | A,C | 0.04 | 0.99 (0.86-1.14) | 0.92 | 1.03 (0.89-1.19) | 0.63 (0.3-1.32) | 0.42 |
| rs7047840 | 110894289 | A,G | 0.04 | 1 (0.87-1.14) | 0.95 | 1.02 (0.88-1.19) | 0.66 (0.31-1.4) | 0.51 |
| rs7048206 | 110894366 | G,A | 0.04 | 1 (0.87-1.14) | 0.95 | 1.02 (0.88-1.19) | 0.66 (0.31-1.4) | 0.51 |
| rs7033853 | 110894500 | A,G | 0.04 | 1 (0.87-1.14) | 0.95 | 1.02 (0.88-1.19) | 0.66 (0.31-1.4) | 0.51 |
| rs72745949 | 110894773 | G,A | 0.04 | 0.93 (0.81-1.06) | 0.25 | 0.96 (0.84-1.1) | 0.46 (0.2-1.04) | 0.13 |
| rs10979229 | 110895080 | G,A | 0.04 | 1 (0.87-1.15) | 0.97 | 1.03 (0.89-1.19) | 0.7 (0.33-1.49) | 0.59 |
| rs484167 | 110895137 | G,A | 0.27 | 0.96 (0.9-1.02) | 0.18 | 0.96 (0.88-1.03) | 0.93 (0.8-1.07) | 0.41 |
| **rs676256** | **110895353** | **A,G** | **0.04** | **0.94 (0.82-1.06)** | **0.30** | **0.95 (0.83-1.09)** | **0.64 (0.3-1.36)** | **0.40** |
| rs10979230 | 110895509 | C,A | 0.04 | 1 (0.87-1.15) | 0.97 | 1.03 (0.89-1.19) | 0.7 (0.33-1.49) | 0.59 |
| rs487887 | 110895527 | C,A | 0.21 | 0.97 (0.9-1.03) | 0.29 | 0.95 (0.87-1.03) | 0.98 (0.82-1.17) | 0.40 |
| rs10979231 | 110895634 | A,G | 0.04 | 1 (0.88-1.15) | 0.97 | 1.04 (0.9-1.2) | 0.61 (0.29-1.27) | 0.32 |
| rs10979233 | 110895705 | C,G | 0.04 | 1 (0.87-1.14) | 0.97 | 1.01 (0.88-1.16) | 0.86 (0.45-1.65) | 0.89 |
| rs3119744 | 110895863 | C,A | 0.04 | 0.93 (0.82-1.05) | 0.23 | 0.95 (0.83-1.09) | 0.53 (0.23-1.18) | 0.22 |
| rs1434838 | 110896141 | G,A | 0.04 | 1 (0.87-1.15) | 0.95 | 1.03 (0.89-1.19) | 0.7 (0.33-1.49) | 0.58 |
| rs1434839 | 110896230 | G,A | 0.04 | 1.01 (0.88-1.16) | 0.89 | 1.03 (0.89-1.19) | 0.78 (0.37-1.62) | 0.72 |
| rs516327 | 110896340 | A,G | 0.23 | 0.98 (0.92-1.04) | 0.52 | 0.97 (0.89-1.05) | 0.99 (0.84-1.17) | 0.69 |
| rs34793195 | 110896525 | A,G | 0.04 | 1.02 (0.89-1.17) | 0.81 | 1.04 (0.9-1.21) | 0.71 (0.33-1.52) | 0.55 |
| rs10979234 | 110896675 | G,A | 0.04 | 1 (0.87-1.15) | 0.95 | 1.03 (0.89-1.19) | 0.7 (0.33-1.49) | 0.58 |
| rs520093 | 110896775 | G,A | 0.08 | 0.96 (0.88-1.06) | 0.47 | 0.98 (0.88-1.09) | 0.8 (0.54-1.19) | 0.54 |
| rs595811 | 110896828 | A,G | 0.08 | 0.96 (0.87-1.05) | 0.38 | 0.97 (0.88-1.08) | 0.8 (0.54-1.19) | 0.50 |
| rs2053148 | 110898048 | C,A | 0.04 | 1.01 (0.88-1.17) | 0.84 | 1.04 (0.9-1.21) | 0.68 (0.31-1.51) | 0.53 |
| rs2117466 | 110898052 | G,A | 0.04 | 1.01 (0.88-1.17) | 0.85 | 1.04 (0.9-1.21) | 0.67 (0.3-1.46) | 0.49 |
| rs10979236 | 110898716 | A,G | 0.04 | 1.01 (0.88-1.16) | 0.92 | 1.05 (0.91-1.21) | 0.57 (0.27-1.23) | 0.26 |
| rs1836458 | 110898887 | A,C | 0.13 | 0.99 (0.92-1.07) | 0.79 | 0.95 (0.87-1.04) | 1.3 (0.96-1.77) | 0.10 |
| rs10979237 | 110898964 | G,A | 0.04 | 1.01 (0.88-1.16) | 0.92 | 1.05 (0.91-1.21) | 0.57 (0.27-1.23) | 0.26 |
| rs12379956 | 110899161 | A,G | 0.04 | 1.01 (0.88-1.16) | 0.89 | 1.05 (0.91-1.22) | 0.57 (0.27-1.23) | 0.26 |
| rs12379962 | 110899191 | A,G | 0.04 | 1.01 (0.88-1.16) | 0.88 | 1.05 (0.91-1.22) | 0.57 (0.27-1.23) | 0.25 |
| rs627610 | 110899291 | A,C | 0.23 | 0.98 (0.92-1.04) | 0.45 | 0.95 (0.88-1.03) | 1 (0.85-1.18) | 0.47 |
| rs10979238 | 110899409 | G,A | 0.04 | 1.04 (0.91-1.19) | 0.59 | 1.06 (0.91-1.22) | 0.84 (0.4-1.77) | 0.67 |
| rs10979239 | 110899675 | C,G | 0.04 | 1.02 (0.89-1.16) | 0.78 | 1.04 (0.91-1.2) | 0.77 (0.39-1.51) | 0.60 |
| rs1434840 | 110899937 | C,A | 0.04 | 1.04 (0.91-1.19) | 0.61 | 1.07 (0.92-1.23) | 0.72 (0.35-1.48) | 0.43 |
| rs641002 | 110900018 | G,A | 0.46 | 0.97 (0.92-1.02) | 0.18 | 0.98 (0.9-1.07) | 0.93 (0.84-1.03) | 0.35 |
| rs1434841 | 110900034 | G,A | 0.04 | 1.03 (0.9-1.18) | 0.64 | 1.06 (0.92-1.23) | 0.72 (0.35-1.48) | 0.45 |
| rs1434842 | 110900060 | A,G | 0.04 | 1.03 (0.9-1.18) | 0.63 | 1.06 (0.92-1.23) | 0.72 (0.35-1.48) | 0.44 |
| rs1836459 | 110900115 | G,A | 0.04 | 1.03 (0.9-1.18) | 0.64 | 1.06 (0.92-1.23) | 0.72 (0.35-1.48) | 0.45 |
| rs35338365 | 110900335 | G,A | 0.04 | 1.03 (0.9-1.18) | 0.64 | 1.06 (0.92-1.23) | 0.72 (0.35-1.48) | 0.45 |
| rs10979242 | 110900392 | G,A | 0.04 | 1.03 (0.9-1.18) | 0.65 | 1.06 (0.92-1.22) | 0.72 (0.35-1.48) | 0.45 |
| rs10979243 | 110900418 | G,A | 0.04 | 1.05 (0.91-1.2) | 0.51 | 1.07 (0.92-1.24) | 0.84 (0.4-1.78) | 0.60 |
| rs10979244 | 110900593 | T,A | 0.04 | 1.04 (0.91-1.19) | 0.58 | 1.07 (0.93-1.24) | 0.72 (0.35-1.48) | 0.41 |
| rs10979245 | 110900744 | G,A | 0.04 | 1.04 (0.91-1.19) | 0.60 | 1.07 (0.92-1.23) | 0.72 (0.35-1.48) | 0.42 |
| rs10979246 | 110901072 | G,C | 0.04 | 1 (0.87-1.14) | 0.96 | 1.02 (0.89-1.18) | 0.67 (0.32-1.41) | 0.52 |
| rs2571501 | 110902296 | A,T | 0.35 | 1.04 (0.99-1.1) | 0.14 | 1.04 (0.96-1.13) | 1.09 (0.96-1.22) | 0.34 |
| rs10979247 | 110903322 | A,G | 0.04 | 1.03 (0.9-1.17) | 0.70 | 1.05 (0.91-1.22) | 0.75 (0.37-1.5) | 0.53 |
| rs12380087 | 110903380 | C,A | 0.04 | 1.04 (0.9-1.18) | 0.62 | 1.07 (0.92-1.23) | 0.72 (0.35-1.48) | 0.43 |
| rs7853556 | 110903440 | A,G | 0.12 | 0.98 (0.91-1.06) | 0.63 | 0.94 (0.86-1.03) | 1.26 (0.92-1.71) | 0.13 |
| rs10979248 | 110904528 | A,G | 0.04 | 1.01 (0.89-1.15) | 0.91 | 1.02 (0.89-1.17) | 0.87 (0.47-1.61) | 0.86 |
| rs537744 | 110904645 | C,G | 0.47 | 0.97 (0.92-1.03) | 0.31 | 1 (0.91-1.09) | 0.94 (0.85-1.05) | 0.47 |
| rs567032 | 110905570 | A,G | 0.49 | 1.02 (0.97-1.08) | 0.39 | 1.05 (0.96-1.15) | 1.05 (0.94-1.16) | 0.51 |
| rs567960 | 110905663 | G,A | 0.47 | 1.02 (0.97-1.08) | 0.41 | 1.03 (0.95-1.13) | 1.04 (0.94-1.16) | 0.67 |
| rs479410 | 110905747 | A,T | 0.46 | 1.02 (0.97-1.08) | 0.36 | 1.04 (0.95-1.13) | 1.05 (0.94-1.16) | 0.63 |
| rs637397 | 110905812 | G,A | 0.47 | 0.97 (0.92-1.03) | 0.31 | 0.99 (0.91-1.08) | 0.95 (0.85-1.05) | 0.52 |
| rs59244432 | 110906257 | A,G | 0.02 | 0.98 (0.83-1.16) | 0.81 | 0.97 (0.81-1.16) | 1.18 (0.46-3.01) | 0.87 |
| rs651808 | 110906666 | A,G | 0.46 | 1.03 (0.97-1.08) | 0.32 | 1.04 (0.96-1.13) | 1.05 (0.95-1.17) | 0.56 |
| rs651829 | 110906677 | A,G | 0.47 | 0.98 (0.93-1.03) | 0.34 | 1 (0.91-1.09) | 0.95 (0.86-1.05) | 0.53 |
| rs10816634 | 110907407 | A,G | 0.04 | 0.99 (0.87-1.13) | 0.88 | 1.01 (0.87-1.16) | 0.77 (0.38-1.57) | 0.76 |
| rs10979249 | 110907520 | C,A | 0.04 | 0.98 (0.86-1.12) | 0.78 | 1 (0.87-1.15) | 0.8 (0.42-1.53) | 0.80 |
| rs10979251 | 110909319 | A,G | 0.05 | 1 (0.88-1.13) | 0.97 | 1.02 (0.89-1.17) | 0.76 (0.4-1.42) | 0.64 |
| rs10816635 | 110910044 | G,A | 0.04 | 1.02 (0.89-1.16) | 0.81 | 1.04 (0.9-1.19) | 0.83 (0.44-1.55) | 0.73 |
| rs602325 | 110910506 | T,A | 0.35 | 1.05 (0.99-1.11) | 0.08 | 1.07 (0.99-1.15) | 1.09 (0.97-1.23) | 0.18 |
| rs539270 | 110910792 | G,A | 0.45 | 1.03 (0.97-1.08) | 0.33 | 1.06 (0.98-1.16) | 1.04 (0.94-1.16) | 0.35 |
| rs10512365 | 110911118 | G,A | 0.04 | 1.02 (0.89-1.16) | 0.79 | 1.04 (0.9-1.19) | 0.81 (0.43-1.55) | 0.69 |
| rs542808 | 110911155 | G,A | 0.35 | 1.04 (0.99-1.1) | 0.12 | 1.06 (0.98-1.15) | 1.07 (0.95-1.21) | 0.26 |
| rs28496063 | 110911169 | G,A | 0.04 | 1.02 (0.89-1.16) | 0.79 | 1.04 (0.9-1.19) | 0.81 (0.43-1.55) | 0.69 |
| rs28550561 | 110911197 | A,G | 0.07 | 1 (0.9-1.12) | 0.95 | 0.99 (0.88-1.12) | 1.1 (0.7-1.74) | 0.91 |
| rs566510 | 110911435 | A,G | 0.49 | 0.98 (0.93-1.03) | 0.35 | 1.04 (0.95-1.13) | 0.95 (0.85-1.05) | 0.16 |
| rs10115125 | 110911908 | G,C | 0.05 | 0.99 (0.87-1.12) | 0.86 | 1.01 (0.88-1.15) | 0.79 (0.42-1.49) | 0.75 |
| rs633082 | 110912805 | C,G | 0.47 | 1.04 (0.98-1.09) | 0.19 | 1.06 (0.97-1.16) | 1.07 (0.96-1.19) | 0.33 |
| rs635667 | 110913378 | A,G | 0.45 | 1.02 (0.97-1.08) | 0.37 | 1.04 (0.95-1.13) | 1.05 (0.94-1.16) | 0.61 |
| rs677872 | 110914540 | G,A | 0.46 | 0.98 (0.93-1.03) | 0.35 | 1.02 (0.94-1.11) | 0.94 (0.85-1.05) | 0.28 |
| rs681015 | 110915234 | A,G | 0.47 | 1.04 (0.99-1.1) | 0.10 | 1.09 (1-1.19) | 1.09 (0.98-1.21) | 0.13 |
| rs586559 | 110915881 | A,G | 0.46 | 0.98 (0.93-1.03) | 0.40 | 1.03 (0.94-1.12) | 0.95 (0.86-1.05) | 0.25 |
| rs614851 | 110917627 | A,G | 0.47 | 1.03 (0.98-1.09) | 0.23 | 1.07 (0.98-1.16) | 1.06 (0.96-1.18) | 0.31 |
| rs10816636 | 110918637 | A,C | 0.05 | 0.96 (0.85-1.08) | 0.48 | 0.99 (0.87-1.12) | 0.65 (0.36-1.17) | 0.35 |
| rs7850979 | 110920305 | G,A | 0.08 | 0.97 (0.87-1.07) | 0.51 | 0.96 (0.86-1.07) | 1 (0.66-1.52) | 0.75 |
| rs7866211 | 110920309 | A,G | 0.08 | 0.97 (0.87-1.07) | 0.51 | 0.96 (0.86-1.07) | 1 (0.66-1.52) | 0.75 |
| rs113741411 | 110920364 | A,T | 0.48 | 0.98 (0.93-1.03) | 0.49 | 1.05 (0.96-1.15) | 0.96 (0.86-1.06) | 0.14 |
| rs1434843 | 110920807 | G,A | 0.05 | 0.95 (0.84-1.06) | 0.36 | 0.97 (0.85-1.09) | 0.69 (0.37-1.26) | 0.42 |
| rs676083 | 110922092 | G,A | 0.45 | 0.98 (0.93-1.03) | 0.40 | 1.04 (0.96-1.13) | 0.94 (0.85-1.05) | 0.14 |
| rs10512363 | 110922828 | A,G | 0.06 | 0.97 (0.86-1.08) | 0.55 | 0.99 (0.87-1.12) | 0.73 (0.41-1.29) | 0.54 |
| rs525803 | 110923883 | A,G | 0.47 | 0.98 (0.93-1.03) | 0.42 | 1.05 (0.96-1.14) | 0.95 (0.86-1.05) | 0.12 |
| rs837993 | 110924767 | A,C | 0.47 | 0.98 (0.93-1.03) | 0.47 | 1.04 (0.95-1.13) | 0.96 (0.86-1.06) | 0.23 |
| rs500708 | 110925107 | A,G | 0.47 | 0.98 (0.93-1.03) | 0.45 | 1.02 (0.94-1.11) | 0.96 (0.86-1.06) | 0.38 |
| rs630611 | 110925407 | A,C | 0.05 | 0.94 (0.83-1.07) | 0.35 | 0.95 (0.84-1.09) | 0.73 (0.35-1.53) | 0.56 |
| rs563471 | 110925919 | A,G | 0.47 | 1.04 (0.98-1.09) | 0.18 | 1.08 (0.99-1.18) | 1.07 (0.96-1.19) | 0.21 |
| rs837994 | 110926669 | C,A | 0.05 | 0.93 (0.82-1.05) | 0.23 | 0.95 (0.83-1.08) | 0.5 (0.21-1.18) | 0.20 |
| rs7030021 | 110927072 | C,A | 0.05 | 1.01 (0.9-1.15) | 0.82 | 1.02 (0.9-1.17) | 0.94 (0.53-1.68) | 0.92 |
| rs13290910 | 110927519 | G,A | 0.05 | 1.02 (0.9-1.15) | 0.79 | 1.02 (0.9-1.16) | 0.95 (0.54-1.68) | 0.92 |
| rs510082 | 110927520 | G,A | 0.45 | 0.97 (0.92-1.02) | 0.22 | 1.01 (0.92-1.09) | 0.93 (0.84-1.03) | 0.25 |
| rs511142 | 110927695 | A,G | 0.46 | 0.97 (0.92-1.02) | 0.24 | 1.03 (0.94-1.12) | 0.93 (0.84-1.03) | 0.12 |
| rs671618 | 110927732 | C,A | 0.47 | 1.04 (0.98-1.09) | 0.17 | 1.09 (1-1.19) | 1.07 (0.96-1.19) | 0.16 |
| rs687992 | 110929030 | A,G | 0.48 | 1.04 (0.99-1.1) | 0.12 | 1.09 (1-1.18) | 1.08 (0.98-1.2) | 0.15 |
| rs7038715 | 110929199 | G,A | 0.07 | 0.97 (0.88-1.07) | 0.54 | 0.96 (0.86-1.07) | 1 (0.67-1.5) | 0.77 |
| rs7027772 | 110929605 | A,G | 0.08 | 0.96 (0.87-1.06) | 0.47 | 0.94 (0.84-1.05) | 1.08 (0.76-1.55) | 0.47 |
| rs593924 | 110929785 | A,T | 0.45 | 0.97 (0.92-1.03) | 0.31 | 1.02 (0.94-1.11) | 0.94 (0.84-1.04) | 0.21 |
| rs573308 | 110929842 | G,C | 0.45 | 0.97 (0.92-1.02) | 0.24 | 1.02 (0.94-1.11) | 0.93 (0.84-1.03) | 0.18 |
| rs527246 | 110932294 | G,A | 0.45 | 0.98 (0.93-1.03) | 0.47 | 1.02 (0.94-1.11) | 0.95 (0.86-1.06) | 0.34 |
| rs626290 | 110932412 | G,A | 0.46 | 1.03 (0.98-1.09) | 0.26 | 1.09 (1-1.18) | 1.05 (0.95-1.17) | 0.16 |
| rs477917 | 110936038 | G,C | 0.47 | 1.04 (0.98-1.09) | 0.17 | 1.08 (0.99-1.18) | 1.07 (0.97-1.19) | 0.20 |
| rs509311 | 110937131 | A,C | 0.47 | 1.04 (0.98-1.09) | 0.18 | 1.1 (1.01-1.2) | 1.07 (0.96-1.18) | 0.10 |
| rs10816637 | 110937650 | C,A | 0.47 | 1.03 (0.98-1.09) | 0.23 | 1.09 (1-1.19) | 1.06 (0.95-1.18) | 0.15 |
| rs1434844 | 110938877 | T,A | 0.07 | 1 (0.9-1.11) | 0.96 | 1 (0.89-1.12) | 1.05 (0.69-1.59) | 0.97 |
| rs10979261 | 110939138 | A,G | 0.05 | 1.03 (0.91-1.16) | 0.62 | 1.05 (0.92-1.19) | 0.87 (0.48-1.58) | 0.68 |
| rs78025103 | 110939265 | G,A | 0.03 | 1.02 (0.87-1.2) | 0.78 | 1.06 (0.89-1.25) | 0.57 (0.19-1.66) | 0.45 |
| rs1434846 | 110939308 | A,G | 0.07 | 1 (0.9-1.11) | 0.96 | 1 (0.89-1.12) | 1.05 (0.69-1.59) | 0.97 |
| rs503238 | 110940013 | G,A | 0.44 | 1.03 (0.98-1.09) | 0.22 | 1.11 (1.02-1.21) | 1.05 (0.94-1.17) | 0.05 |
| rs10979262 | 110940990 | T,A | 0.07 | 1 (0.9-1.11) | 1.00 | 0.99 (0.89-1.11) | 1.05 (0.67-1.65) | 0.97 |
| rs12380632 | 110941095 | A,G | 0.08 | 0.98 (0.89-1.09) | 0.76 | 0.98 (0.88-1.1) | 0.97 (0.65-1.43) | 0.95 |
| rs558016 | 110941382 | G,A | 0.04 | 1.07 (0.94-1.21) | 0.33 | 1.09 (0.96-1.25) | 0.73 (0.33-1.61) | 0.31 |
| rs666303 | 110941390 | A,G | 0.49 | 1.02 (0.97-1.08) | 0.42 | 1.04 (0.95-1.14) | 1.04 (0.94-1.16) | 0.62 |
| rs77889939 | 110942176 | T,A | 0.06 | 0.99 (0.89-1.1) | 0.82 | 0.98 (0.88-1.11) | 1 (0.6-1.68) | 0.97 |
| rs683466 | 110942879 | G,A | 0.05 | 0.99 (0.87-1.12) | 0.85 | 1 (0.88-1.14) | 0.79 (0.36-1.73) | 0.83 |
| rs72745970 | 110943026 | G,A | 0.01 | 1.03 (0.83-1.28) | 0.80 | #N/A | #N/A | #N/A |
| rs10979263 | 110943815 | A,G | 0.48 | 1.03 (0.98-1.09) | 0.26 | 1.07 (0.98-1.17) | 1.06 (0.96-1.18) | 0.33 |
| rs10979264 | 110944986 | G,A | 0.47 | 1.02 (0.97-1.08) | 0.43 | 1.05 (0.97-1.15) | 1.04 (0.94-1.15) | 0.50 |
| rs607019 | 110945296 | G,A | 0.04 | 0.98 (0.86-1.11) | 0.76 | 0.99 (0.87-1.14) | 0.76 (0.35-1.64) | 0.78 |
| rs607056 | 110945330 | G,A | 0.04 | 0.98 (0.87-1.11) | 0.78 | 1 (0.87-1.14) | 0.76 (0.35-1.64) | 0.78 |
| rs10759273 | 110946296 | A,G | 0.48 | 1.03 (0.98-1.08) | 0.27 | 1.08 (0.99-1.19) | 1.06 (0.95-1.17) | 0.20 |
| rs559836 | 110946380 | G,A | 0.04 | 0.99 (0.87-1.12) | 0.82 | 1 (0.87-1.14) | 0.76 (0.35-1.64) | 0.78 |
| rs10816640 | 110946857 | G,A | 0.46 | 1.03 (0.98-1.08) | 0.30 | 1.06 (0.97-1.16) | 1.05 (0.95-1.17) | 0.38 |
| rs553333 | 110947155 | G,A | 0.05 | 0.98 (0.87-1.1) | 0.70 | 1 (0.88-1.14) | 0.67 (0.36-1.26) | 0.45 |
| rs864145 | 110948023 | G,C | 0.04 | 0.99 (0.87-1.12) | 0.83 | 1 (0.88-1.14) | 0.77 (0.36-1.65) | 0.79 |
| rs7875207 | 110948812 | G,A | 0.01 | 0.98 (0.77-1.24) | 0.87 | #N/A | #N/A | #N/A |
| rs10816642 | 110950488 | A,G | 0.41 | 1.01 (0.96-1.07) | 0.64 | 1.06 (0.98-1.15) | 1.01 (0.91-1.12) | 0.33 |
| rs7022714 | 110950572 | G,A | 0.50 | 1.01 (0.96-1.06) | 0.80 | 1.05 (0.96-1.15) | 1.01 (0.91-1.12) | 0.44 |
| rs13295544 | 110953139 | A,G | 0.45 | 1.02 (0.97-1.07) | 0.49 | 1.08 (0.99-1.17) | 1.03 (0.92-1.14) | 0.21 |
| rs28450518 | 110953584 | T,A | 0.03 | 0.97 (0.83-1.13) | 0.66 | 0.99 (0.84-1.16) | 0.56 (0.2-1.61) | 0.54 |
| rs669329 | 110954331 | G,A | 0.49 | 0.99 (0.94-1.04) | 0.69 | 1.02 (0.93-1.11) | 0.98 (0.88-1.08) | 0.69 |
| rs550891 | 110954663 | C,A | 0.07 | 1 (0.9-1.1) | 0.95 | 1.01 (0.91-1.12) | 0.85 (0.51-1.41) | 0.79 |
| rs59860864 | 110954925 | C,A | 0.01 | 0.85 (0.66-1.1) | 0.20 | 0.91 (0.69-1.21) | 0.35 (0.1-1.27) | 0.19 |
| rs13287026 | 110955043 | G,A | 0.43 | 1.02 (0.97-1.07) | 0.47 | 1.04 (0.96-1.13) | 1.03 (0.93-1.15) | 0.61 |
| rs556399 | 110955288 | A,G | 0.09 | 0.95 (0.87-1.04) | 0.30 | 0.96 (0.87-1.06) | 0.85 (0.59-1.23) | 0.54 |
| rs34979687 | 110955816 | A,G | 0.01 | 0.83 (0.64-1.08) | 0.16 | 0.91 (0.69-1.21) | 0.24 (0.05-1.09) | 0.09 |
| rs638978 | 110956479 | G,A | 0.45 | 0.99 (0.94-1.05) | 0.78 | 1 (0.92-1.09) | 0.98 (0.89-1.09) | 0.90 |
| rs75802750 | 110956500 | G,A | 0.01 | 0.82 (0.63-1.07) | 0.15 | 0.9 (0.68-1.2) | 0.24 (0.05-1.09) | 0.09 |
| rs625647 | 110957231 | C,A | 0.09 | 0.94 (0.86-1.03) | 0.18 | 0.97 (0.88-1.08) | 0.68 (0.47-1) | 0.13 |
| rs3983564 | 110957368 | T,A | 0.48 | 0.99 (0.94-1.04) | 0.63 | 0.98 (0.89-1.07) | 0.98 (0.88-1.08) | 0.85 |
| rs507584 | 110957372 | T,A | 0.45 | 0.99 (0.94-1.04) | 0.72 | 1 (0.92-1.09) | 0.98 (0.88-1.09) | 0.90 |
| rs538688 | 110958479 | A,C | 0.46 | 0.99 (0.94-1.04) | 0.64 | 1 (0.92-1.09) | 0.97 (0.88-1.08) | 0.83 |
| rs10979270 | 110959027 | A,G | 0.13 | 1.01 (0.94-1.1) | 0.71 | 1.04 (0.95-1.14) | 0.91 (0.7-1.18) | 0.49 |
| rs569795 | 110959560 | A,G | 0.09 | 0.95 (0.86-1.04) | 0.27 | 0.99 (0.89-1.09) | 0.68 (0.46-1) | 0.14 |
| rs569859 | 110959588 | G,A | 0.08 | 0.95 (0.86-1.04) | 0.27 | 0.99 (0.89-1.1) | 0.63 (0.42-0.95) | 0.08 |
| rs581561 | 110960080 | C,A | 0.07 | 0.99 (0.89-1.09) | 0.79 | 1 (0.9-1.12) | 0.81 (0.47-1.4) | 0.75 |
| rs507242 | 110969263 | A,G | 0.48 | 0.98 (0.93-1.03) | 0.51 | 0.97 (0.89-1.05) | 0.97 (0.87-1.07) | 0.73 |
| rs838012 | 110969777 | G,A | 0.46 | 0.99 (0.94-1.04) | 0.62 | 0.99 (0.91-1.07) | 0.97 (0.88-1.08) | 0.89 |
| rs10816648 | 110971140 | A,G | 0.44 | 1.04 (0.98-1.09) | 0.19 | 1.06 (0.98-1.16) | 1.07 (0.96-1.19) | 0.31 |
| rs35517812 | 110971386 | G,A | 0.01 | 0.74 (0.56-0.97) | 0.03 | 0.78 (0.58-1.05) | 0.34 (0.09-1.23) | 0.06 |
| rs485572 | 110972062 | C,G | 0.46 | 0.99 (0.94-1.04) | 0.63 | 0.98 (0.9-1.06) | 0.98 (0.88-1.08) | 0.86 |
| rs539221 | 110972292 | A,C | 0.08 | 0.96 (0.88-1.06) | 0.44 | 0.97 (0.87-1.07) | 0.9 (0.58-1.41) | 0.73 |
| rs10979274 | 110973879 | A,G | 0.44 | 1.04 (0.99-1.1) | 0.15 | 1.07 (0.98-1.16) | 1.07 (0.97-1.19) | 0.26 |
| rs609982 | 110974043 | G,C | 0.09 | 0.94 (0.86-1.03) | 0.21 | 0.97 (0.87-1.07) | 0.72 (0.49-1.06) | 0.21 |
| rs73998 | 110974433 | C,A | 0.45 | 0.98 (0.93-1.04) | 0.52 | 0.97 (0.89-1.06) | 0.97 (0.87-1.08) | 0.78 |
| rs10979275 | 110975315 | G,A | 0.44 | 1.04 (0.98-1.09) | 0.17 | 1.07 (0.98-1.16) | 1.07 (0.96-1.19) | 0.27 |
| rs548698 | 110975463 | C,A | 0.45 | 0.99 (0.94-1.04) | 0.66 | 0.99 (0.91-1.07) | 0.98 (0.88-1.09) | 0.90 |
| rs838001 | 110975498 | A,C | 0.48 | 0.99 (0.94-1.04) | 0.62 | 0.98 (0.9-1.07) | 0.97 (0.88-1.08) | 0.88 |
| rs838002 | 110975878 | A,G | 0.47 | 0.99 (0.94-1.04) | 0.59 | 0.97 (0.89-1.06) | 0.97 (0.88-1.08) | 0.79 |
| rs10979276 | 110975982 | A,G | 0.47 | 0.99 (0.94-1.04) | 0.63 | 0.97 (0.89-1.06) | 0.98 (0.88-1.08) | 0.80 |
| rs73519684 | 110976133 | A,C | 0.01 | 0.74 (0.56-0.97) | 0.03 | 0.77 (0.57-1.04) | 0.37 (0.1-1.37) | 0.07 |
| rs498727 | 110976981 | A,G | 0.46 | 0.98 (0.93-1.03) | 0.47 | 0.97 (0.89-1.06) | 0.96 (0.87-1.07) | 0.76 |
| rs501345 | 110977231 | G,A | 0.48 | 0.98 (0.93-1.03) | 0.44 | 0.98 (0.9-1.07) | 0.96 (0.86-1.07) | 0.74 |
| rs656048 | 110977391 | A,G | 0.46 | 0.98 (0.93-1.04) | 0.52 | 0.98 (0.9-1.06) | 0.97 (0.87-1.07) | 0.80 |
| rs7026619 | 110978841 | A,C | 0.46 | 1 (0.95-1.05) | 0.97 | 1.02 (0.94-1.11) | 1 (0.9-1.11) | 0.87 |
| rs672586 | 110978859 | G,A | 0.43 | 1 (0.95-1.06) | 0.94 | 1.03 (0.95-1.12) | 1 (0.9-1.11) | 0.73 |
| rs4246873 | 110980594 | A,G | 0.50 | 0.98 (0.93-1.03) | 0.44 | 0.98 (0.9-1.08) | 0.96 (0.87-1.06) | 0.74 |
| rs62567152 | 110982252 | G,A | 0.36 | 1.01 (0.96-1.07) | 0.65 | 1.01 (0.94-1.1) | 1.02 (0.91-1.15) | 0.90 |
| rs519144 | 110986403 | G,A | 0.50 | 1.01 (0.96-1.06) | 0.71 | 1 (0.91-1.09) | 1.02 (0.92-1.13) | 0.87 |
| rs10979283 | 110987236 | C,A | 0.45 | 1.01 (0.96-1.07) | 0.64 | 1.03 (0.95-1.13) | 1.02 (0.92-1.13) | 0.76 |
| rs837999 | 110989319 | C,A | 0.41 | 0.99 (0.94-1.04) | 0.59 | 0.98 (0.91-1.07) | 0.97 (0.87-1.08) | 0.87 |
| rs868895 | 110989880 | A,C | 0.47 | 0.98 (0.94-1.04) | 0.56 | 0.98 (0.9-1.07) | 0.97 (0.87-1.08) | 0.84 |
| rs1888913 | 110990323 | A,C | 0.05 | 0.99 (0.88-1.12) | 0.88 | 0.99 (0.87-1.13) | 1 (0.59-1.69) | 0.99 |
| rs75594954 | 110999079 | A,G | 0.01 | 0.84 (0.65-1.09) | 0.18 | 0.86 (0.64-1.14) | 0.62 (0.21-1.84) | 0.40 |
| rs35034713 | 111000704 | A,G | 0.47 | 1.02 (0.97-1.08) | 0.42 | 1.04 (0.95-1.13) | 1.04 (0.94-1.16) | 0.67 |
| rs7847854 | 111002172 | G,A | 0.49 | 0.99 (0.94-1.05) | 0.77 | 1.01 (0.92-1.1) | 0.98 (0.89-1.09) | 0.90 |
| rs1360533 | 111004738 | G,A | 0.41 | 0.97 (0.92-1.03) | 0.33 | 1 (0.92-1.08) | 0.94 (0.84-1.05) | 0.45 |
| rs1556472 | 111005974 | A,T | 0.49 | 0.99 (0.94-1.05) | 0.84 | 1 (0.92-1.1) | 0.99 (0.89-1.1) | 0.96 |
| rs4644328 | 111008699 | A,G | 0.08 | 1.04 (0.95-1.14) | 0.41 | 1.03 (0.93-1.15) | 1.14 (0.76-1.7) | 0.69 |
| rs4382542 | 111008909 | G,A | 0.08 | 1.05 (0.95-1.15) | 0.35 | 1.04 (0.94-1.15) | 1.16 (0.77-1.76) | 0.61 |
| rs1332299 | 111012416 | G,A | 0.46 | 0.99 (0.94-1.04) | 0.72 | 1 (0.92-1.09) | 0.98 (0.88-1.09) | 0.90 |
| rs1332300 | 111012582 | G,C | 0.08 | 1.05 (0.95-1.15) | 0.34 | 1.04 (0.94-1.15) | 1.16 (0.76-1.78) | 0.61 |
| rs10979291 | 111014868 | A,G | 0.50 | 1 (0.95-1.05) | 0.86 | 1 (0.92-1.09) | 0.99 (0.89-1.1) | 0.98 |
| rs1332302 | 111015811 | A,T | 0.11 | 1.04 (0.96-1.13) | 0.38 | 1.04 (0.95-1.14) | 1.07 (0.8-1.43) | 0.68 |
| rs7034951 | 111016395 | A,G | 0.08 | 1.04 (0.95-1.15) | 0.37 | 1.04 (0.94-1.15) | 1.12 (0.75-1.68) | 0.66 |
| rs7043626 | 111018343 | A,C | 0.09 | 1.04 (0.95-1.14) | 0.38 | 1.01 (0.92-1.12) | 1.36 (0.91-2.02) | 0.31 |
| rs1537286 | 111021175 | G,A | 0.08 | 1.04 (0.95-1.15) | 0.38 | 1.03 (0.93-1.15) | 1.21 (0.79-1.85) | 0.59 |
| rs7030526 | 111022525 | G,A | 0.42 | 0.98 (0.93-1.03) | 0.35 | 1 (0.92-1.09) | 0.94 (0.84-1.05) | 0.46 |
| rs10739255 | 111023899 | G,A | 0.39 | 0.98 (0.93-1.03) | 0.39 | 1 (0.93-1.09) | 0.94 (0.84-1.05) | 0.47 |
| rs10739256 | 111024123 | G,A | 0.11 | 1.04 (0.95-1.12) | 0.40 | 1.02 (0.93-1.12) | 1.13 (0.85-1.51) | 0.63 |
| rs10759275 | 111024438 | C,A | 0.11 | 1.03 (0.95-1.12) | 0.42 | 1.03 (0.93-1.13) | 1.12 (0.84-1.48) | 0.68 |
| rs10979297 | 111024683 | T,A | 0.08 | 0.96 (0.87-1.06) | 0.40 | 0.95 (0.85-1.06) | 0.99 (0.68-1.45) | 0.64 |
| rs1758617 | 111025649 | G,A | 0.39 | 0.98 (0.93-1.03) | 0.44 | 1.01 (0.93-1.09) | 0.94 (0.85-1.06) | 0.49 |
| rs2778252 | 111026158 | A,G | 0.09 | 1.03 (0.94-1.13) | 0.55 | 1.02 (0.93-1.13) | 1.08 (0.76-1.55) | 0.83 |
| rs13283710 | 111026582 | G,A | 0.50 | 1 (0.95-1.05) | 0.89 | 1.01 (0.93-1.11) | 0.99 (0.89-1.1) | 0.89 |
| rs3117888 | 111027827 | A,G | 0.11 | 1.04 (0.96-1.13) | 0.39 | 1.04 (0.95-1.14) | 1.07 (0.79-1.43) | 0.69 |
| rs2778253 | 111028359 | C,G | 0.38 | 0.98 (0.93-1.04) | 0.53 | 1 (0.92-1.08) | 0.96 (0.86-1.07) | 0.75 |
| rs2778254 | 111028362 | C,A | 0.38 | 0.98 (0.93-1.04) | 0.55 | 1 (0.92-1.08) | 0.96 (0.86-1.07) | 0.75 |
| rs3117889 | 111028604 | G,C | 0.39 | 0.98 (0.93-1.03) | 0.46 | 1.01 (0.93-1.09) | 0.95 (0.85-1.06) | 0.50 |
| rs3119743 | 111028887 | A,T | 0.39 | 0.98 (0.93-1.03) | 0.45 | 1.01 (0.93-1.09) | 0.95 (0.85-1.06) | 0.49 |
| rs1330591 | 111029103 | A,G | 0.12 | 1.03 (0.95-1.11) | 0.47 | 1.03 (0.94-1.13) | 1.04 (0.79-1.36) | 0.75 |
| rs2482603 | 111029969 | A,G | 0.11 | 1.02 (0.93-1.1) | 0.72 | 1.02 (0.93-1.12) | 1.01 (0.75-1.36) | 0.92 |
| rs3117890 | 111031148 | G,A | 0.11 | 1.04 (0.96-1.13) | 0.31 | 1.05 (0.95-1.15) | 1.07 (0.8-1.43) | 0.59 |
| rs645119 | 111031550 | A,G | 0.10 | 1.02 (0.93-1.1) | 0.72 | 1.02 (0.92-1.12) | 1.02 (0.75-1.39) | 0.93 |
| rs2778255 | 111032311 | G,A | 0.38 | 0.98 (0.93-1.04) | 0.57 | 1 (0.92-1.08) | 0.96 (0.86-1.08) | 0.77 |
| rs1323397 | 111034788 | A,G | 0.07 | 0.96 (0.87-1.06) | 0.43 | 0.95 (0.85-1.07) | 0.96 (0.64-1.45) | 0.71 |
| rs9299149 | 111034825 | C,A | 0.08 | 1.04 (0.95-1.14) | 0.38 | 1.04 (0.94-1.15) | 1.12 (0.74-1.68) | 0.67 |
| rs7039993 | 111034920 | G,C | 0.03 | 1.06 (0.9-1.25) | 0.50 | 1.07 (0.89-1.27) | 0.99 (0.35-2.84) | 0.78 |
| rs10759277 | 111035445 | G,A | 0.48 | 0.99 (0.94-1.05) | 0.79 | 1 (0.92-1.09) | 0.98 (0.89-1.09) | 0.93 |
| rs1575449 | 111037191 | A,G | 0.02 | 1.1 (0.9-1.34) | 0.34 | 1.14 (0.92-1.4) | 0.66 (0.18-2.48) | 0.39 |
| rs1323374 | 111037829 | A,T | 0.46 | 1 (0.95-1.05) | 0.99 | 1.01 (0.93-1.1) | 1 (0.9-1.11) | 0.92 |
| rs10979300 | 111040235 | G,A | 0.30 | 1 (0.94-1.05) | 0.90 | 1.01 (0.93-1.09) | 0.98 (0.86-1.12) | 0.93 |
| rs1323376 | 111042771 | G,A | 0.08 | 1.06 (0.96-1.16) | 0.25 | 1.06 (0.96-1.17) | 1.1 (0.74-1.64) | 0.51 |
| rs1323377 | 111043009 | A,G | 0.08 | 1.06 (0.96-1.16) | 0.25 | 1.05 (0.95-1.17) | 1.12 (0.75-1.68) | 0.52 |
| rs10979301 | 111044933 | A,G | 0.05 | 0.99 (0.88-1.12) | 0.85 | 0.97 (0.86-1.11) | 1.18 (0.62-2.25) | 0.81 |
| rs12552629 | 111047312 | A,G | 0.07 | 0.97 (0.88-1.08) | 0.62 | 0.97 (0.86-1.09) | 0.99 (0.64-1.54) | 0.86 |
| rs7852572 | 111047463 | G,A | 0.11 | 1.04 (0.96-1.14) | 0.32 | 1.06 (0.96-1.17) | 0.99 (0.72-1.36) | 0.48 |
| rs7021201 | 111050366 | G,A | 0.08 | 0.98 (0.89-1.07) | 0.62 | 0.97 (0.88-1.08) | 0.96 (0.67-1.38) | 0.88 |
| rs10979302 | 111050704 | C,A | 0.50 | 1 (0.95-1.05) | 0.90 | 1.02 (0.94-1.12) | 0.99 (0.89-1.1) | 0.77 |
| rs12353336 | 111050810 | G,A | 0.05 | 0.99 (0.88-1.11) | 0.85 | 0.96 (0.84-1.09) | 1.43 (0.8-2.57) | 0.38 |
| rs10117055 | 111051418 | G,A | 0.41 | 1.01 (0.96-1.06) | 0.78 | 1.02 (0.94-1.11) | 1.01 (0.91-1.13) | 0.88 |
| rs10114572 | 111051611 | A,G | 0.43 | 1 (0.95-1.06) | 0.88 | 1.03 (0.94-1.11) | 1 (0.9-1.11) | 0.80 |
| rs10117687 | 111051626 | T,A | 0.05 | 0.98 (0.87-1.11) | 0.73 | 0.96 (0.84-1.09) | 1.24 (0.65-2.37) | 0.66 |
| rs73653749 | 111051751 | A,G | 0.02 | 1.06 (0.87-1.29) | 0.54 | 1.09 (0.89-1.34) | 0.66 (0.18-2.48) | 0.56 |
| rs4978678 | 111053537 | A,C | 0.05 | 1 (0.89-1.12) | 0.97 | 0.98 (0.86-1.11) | 1.24 (0.66-2.34) | 0.76 |
| rs56125520 | 111054295 | A,C | 0.03 | 0.99 (0.85-1.15) | 0.90 | 1.02 (0.87-1.2) | 0.62 (0.26-1.52) | 0.54 |
| rs7046291 | 111054302 | C,G | 0.49 | 1.01 (0.95-1.06) | 0.83 | 1.03 (0.94-1.13) | 1.01 (0.91-1.12) | 0.77 |
| rs10979304 | 111055670 | G,A | 0.50 | 1 (0.95-1.05) | 0.97 | 1.02 (0.93-1.11) | 1 (0.9-1.11) | 0.90 |
| rs55896475 | 111056782 | G,A | 0.03 | 1 (0.85-1.16) | 0.97 | 1.04 (0.88-1.22) | 0.54 (0.21-1.37) | 0.35 |
| rs13300950 | 111057524 | G,A | 0.49 | 1 (0.94-1.05) | 0.86 | 1.01 (0.93-1.11) | 0.99 (0.89-1.1) | 0.87 |
| rs1323380 | 111060921 | C,A | 0.03 | 1 (0.86-1.15) | 0.99 | 1.04 (0.89-1.21) | 0.59 (0.26-1.35) | 0.38 |
| rs1885973 | 111061706 | A,T | 0.46 | 1 (0.95-1.05) | 0.92 | 1.02 (0.94-1.11) | 0.99 (0.89-1.1) | 0.75 |
| rs10979312 | 111064186 | A,C | 0.05 | 0.96 (0.85-1.08) | 0.48 | 0.97 (0.86-1.09) | 0.82 (0.42-1.61) | 0.73 |
| rs1323381 | 111064438 | G,A | 0.03 | 1.02 (0.89-1.18) | 0.76 | 1.05 (0.9-1.23) | 0.71 (0.33-1.53) | 0.52 |
| rs1407849 | 111064598 | T,A | 0.48 | 0.99 (0.94-1.04) | 0.69 | 1 (0.92-1.1) | 0.98 (0.88-1.09) | 0.86 |
| rs1407850 | 111064801 | A,G | 0.44 | 1 (0.95-1.05) | 0.88 | 1.01 (0.93-1.1) | 0.99 (0.89-1.1) | 0.88 |
| rs1323383 | 111065424 | A,G | 0.43 | 0.99 (0.94-1.05) | 0.84 | 1.01 (0.93-1.1) | 0.98 (0.88-1.09) | 0.83 |
| rs117936512 | 111066476 | C,A | 0.38 | 0.99 (0.94-1.05) | 0.76 | 1.01 (0.93-1.09) | 0.97 (0.87-1.09) | 0.78 |
| rs17543333 | 111066833 | A,C | 0.06 | 1.04 (0.93-1.15) | 0.52 | 1.07 (0.95-1.2) | 0.79 (0.47-1.33) | 0.36 |
| rs16912868 | 111067924 | G,A | 0.44 | 0.98 (0.93-1.03) | 0.47 | 1 (0.92-1.08) | 0.96 (0.86-1.07) | 0.70 |
| rs116991065 | 111070042 | A,G | 0.03 | 1.02 (0.88-1.19) | 0.79 | 1.06 (0.9-1.25) | 0.59 (0.24-1.43) | 0.36 |
| rs2093680 | 111070724 | G,A | 0.11 | 1.01 (0.93-1.09) | 0.89 | 1.03 (0.94-1.13) | 0.86 (0.63-1.17) | 0.46 |
| rs79831337 | 111070857 | A,G | 0.11 | 1.03 (0.95-1.12) | 0.44 | 1.05 (0.96-1.16) | 0.95 (0.71-1.28) | 0.49 |
| rs56020002 | 111071733 | G,A | 0.03 | 1.03 (0.88-1.19) | 0.75 | 1.06 (0.9-1.25) | 0.63 (0.26-1.53) | 0.43 |
| rs10979314 | 111072429 | G,A | 0.27 | 0.98 (0.93-1.04) | 0.56 | 1 (0.92-1.08) | 0.94 (0.82-1.09) | 0.72 |
| rs10979315 | 111072586 | A,G | 0.11 | 1.02 (0.94-1.1) | 0.70 | 1.04 (0.95-1.14) | 0.89 (0.65-1.21) | 0.50 |
| rs12349823 | 111072668 | G,A | 0.11 | 1.01 (0.93-1.1) | 0.76 | 1.04 (0.94-1.14) | 0.89 (0.66-1.22) | 0.56 |
| rs12336489 | 111073103 | A,G | 0.08 | 1.01 (0.92-1.11) | 0.87 | 1.01 (0.92-1.12) | 0.96 (0.6-1.53) | 0.95 |
| rs12336573 | 111073347 | A,G | 0.08 | 1.01 (0.92-1.11) | 0.87 | 1.01 (0.92-1.12) | 0.96 (0.6-1.53) | 0.96 |
| rs10283642 | 111074243 | G,A | 0.08 | 0.95 (0.86-1.05) | 0.29 | 0.98 (0.88-1.08) | 0.68 (0.43-1.08) | 0.23 |
| rs10739257 | 111074457 | C,A | 0.23 | 0.98 (0.92-1.05) | 0.58 | 0.98 (0.9-1.05) | 0.98 (0.83-1.17) | 0.82 |
| rs62570194 | 111075953 | A,G | 0.39 | 1.01 (0.96-1.07) | 0.61 | 0.99 (0.92-1.08) | 1.04 (0.93-1.16) | 0.71 |
| rs7033858 | 111077461 | A,G | 0.49 | 1 (0.95-1.05) | 0.93 | 1 (0.91-1.09) | 1 (0.9-1.1) | 1.00 |
| rs1923964 | 111081694 | C,G | 0.34 | 1.02 (0.96-1.07) | 0.58 | 1.05 (0.97-1.13) | 1 (0.89-1.13) | 0.45 |
| rs4978345 | 111082271 | A,C | 0.40 | 0.98 (0.93-1.03) | 0.42 | 0.96 (0.89-1.04) | 0.97 (0.87-1.08) | 0.59 |
| rs62570197 | 111086510 | G,A | 0.41 | 0.99 (0.94-1.05) | 0.82 | 0.99 (0.91-1.08) | 0.99 (0.89-1.1) | 0.97 |
| rs10816659 | 111092798 | G,A | 0.40 | 0.99 (0.94-1.05) | 0.81 | 0.97 (0.89-1.05) | 1 (0.9-1.11) | 0.70 |
| rs2417854 | 111095847 | C,G | 0.40 | 0.99 (0.94-1.05) | 0.79 | 0.97 (0.9-1.05) | 1 (0.89-1.11) | 0.75 |
| rs1323389 | 111098510 | A,G | 0.40 | 0.99 (0.94-1.04) | 0.70 | 0.97 (0.89-1.05) | 0.99 (0.89-1.1) | 0.73 |
| rs16936302 | 111100826 | A,G | 0.47 | 0.99 (0.94-1.05) | 0.78 | 0.97 (0.89-1.05) | 0.99 (0.89-1.1) | 0.73 |
| rs12550936 | 111106217 | C,A | 0.37 | 0.98 (0.93-1.04) | 0.55 | 0.98 (0.9-1.06) | 0.97 (0.86-1.09) | 0.81 |

**Supplemental Table 2C. Association statistics and effect estimates for 9q31.2 fine-mapping SNPs in breast cancer cases and controls with African ancestry.**

| SNP | Location | Alleles | MAF | Trend OR | P-trend | Het OR | Hom OR | P-geno |
| --- | --- | --- | --- | --- | --- | --- | --- | --- |
| rs2900441 | 110740582 | A,T | 0.50 | 0.96 (0.84-1.09) | 0.51 | 1.11 (0.89-1.38) | 0.92 (0.71-1.18) | 0.21 |
| rs837410 | 110740832 | G,A | 0.13 | 1.14 (0.95-1.38) | 0.16 | 1.2 (0.97-1.49) | 0.94 (0.46-1.95) | 0.22 |
| rs629480 | 110741144 | A,G | 0.11 | 1.14 (0.93-1.39) | 0.20 | 1.21 (0.97-1.51) | 0.87 (0.4-1.86) | 0.22 |
| rs79634968 | 110742192 | G,C | 0.02 | 1.18 (0.71-1.96) | 0.52 | #N/A | #N/A | #N/A |
| rs657659 | 110742851 | A,G | 0.20 | 0.89 (0.76-1.05) | 0.16 | 0.9 (0.75-1.1) | 0.76 (0.48-1.22) | 0.36 |
| rs35871706 | 110745668 | G,A | 0.01 | 0.97 (0.51-1.86) | 0.94 | #N/A | #N/A | #N/A |
| rs7024363 | 110746405 | C,A | 0.49 | 0.95 (0.84-1.08) | 0.46 | 1.1 (0.89-1.36) | 0.9 (0.7-1.16) | 0.21 |
| rs1556939 | 110749585 | G,C | 0.48 | 0.95 (0.83-1.07) | 0.38 | 1.07 (0.86-1.32) | 0.89 (0.69-1.14) | 0.26 |
| rs7029391 | 110751144 | A,G | 0.34 | 1.02 (0.89-1.16) | 0.78 | 1.03 (0.85-1.24) | 1.03 (0.77-1.38) | 0.95 |
| rs480113 | 110751682 | G,A | 0.03 | 0.93 (0.64-1.37) | 0.72 | #N/A | #N/A | #N/A |
| rs1009813 | 110752106 | G,A | 0.25 | 1.06 (0.92-1.23) | 0.43 | 0.99 (0.82-1.19) | 1.32 (0.89-1.94) | 0.34 |
| rs7851574 | 110753079 | A,G | 0.30 | 1.02 (0.89-1.17) | 0.79 | 1.05 (0.87-1.27) | 0.99 (0.72-1.37) | 0.85 |
| rs1934419 | 110754102 | A,G | 0.48 | 1.05 (0.93-1.19) | 0.43 | 1.09 (0.88-1.35) | 1.1 (0.86-1.42) | 0.67 |
| rs1769652 | 110754778 | A,G | 0.15 | 1.08 (0.91-1.29) | 0.38 | 1.14 (0.93-1.4) | 0.94 (0.53-1.67) | 0.44 |
| rs10979155 | 110754824 | A,G | 0.24 | 1.04 (0.9-1.21) | 0.57 | 1 (0.83-1.21) | 1.19 (0.8-1.77) | 0.68 |
| rs75358334 | 110755497 | G,A | 0.02 | 1.15 (0.71-1.85) | 0.58 | #N/A | #N/A | #N/A |
| rs668474 | 110755943 | G,A | 0.09 | 0.94 (0.74-1.19) | 0.60 | 0.88 (0.68-1.13) | 1.81 (0.55-5.95) | 0.34 |
| rs10979157 | 110757887 | G,A | 0.13 | 1.15 (0.96-1.38) | 0.14 | 1.16 (0.93-1.44) | 1.28 (0.68-2.42) | 0.33 |
| rs76449292 | 110758841 | C,G | 0.14 | 1.05 (0.88-1.26) | 0.59 | 1.1 (0.89-1.35) | 0.9 (0.46-1.72) | 0.65 |
| rs13288365 | 110759721 | G,C | 0.10 | 0.87 (0.71-1.07) | 0.20 | 0.87 (0.69-1.1) | 0.79 (0.36-1.75) | 0.44 |
| rs673985 | 110759922 | G,A | 0.13 | 0.94 (0.78-1.13) | 0.49 | 1.09 (0.88-1.35) | 0.4 (0.2-0.81) | 0.02 |
| rs10979158 | 110760000 | A,G | 0.22 | 1 (0.86-1.17) | 0.96 | 1.15 (0.95-1.39) | 0.72 (0.47-1.1) | 0.08 |
| rs4979617 | 110760144 | G,A | 0.17 | 1.07 (0.9-1.27) | 0.45 | 1.13 (0.93-1.39) | 0.89 (0.52-1.55) | 0.41 |
| rs4979618 | 110760316 | A,G | 0.50 | 0.94 (0.83-1.07) | 0.36 | 1.13 (0.91-1.4) | 0.89 (0.69-1.14) | 0.10 |
| rs4979620 | 110760910 | G,C | 0.25 | 1.1 (0.95-1.28) | 0.19 | 1.05 (0.87-1.27) | 1.35 (0.92-1.99) | 0.29 |
| rs12342578 | 110761002 | G,A | 0.19 | 1.13 (0.96-1.33) | 0.13 | 1.15 (0.95-1.4) | 1.21 (0.74-1.99) | 0.30 |
| rs12342611 | 110761104 | C,G | 0.22 | 0.99 (0.85-1.16) | 0.94 | 1.14 (0.94-1.38) | 0.71 (0.47-1.07) | 0.07 |
| rs12347482 | 110761427 | A,C | 0.25 | 1.11 (0.96-1.28) | 0.17 | 1.06 (0.88-1.27) | 1.36 (0.92-1.99) | 0.28 |
| rs12236795 | 110761513 | G,A | 0.25 | 1.1 (0.95-1.27) | 0.22 | 1.05 (0.87-1.27) | 1.31 (0.89-1.93) | 0.37 |
| rs4979621 | 110764599 | G,A | 0.32 | 1.08 (0.94-1.23) | 0.28 | 1.1 (0.91-1.32) | 1.13 (0.83-1.54) | 0.54 |
| rs4979622 | 110764799 | G,A | 0.49 | 0.94 (0.83-1.07) | 0.35 | 1.03 (0.83-1.28) | 0.88 (0.69-1.13) | 0.38 |
| rs4978665 | 110764844 | G,C | 0.05 | 0.96 (0.7-1.31) | 0.80 | #N/A | #N/A | #N/A |
| rs10759270 | 110764981 | G,A | 0.33 | 1.07 (0.94-1.22) | 0.32 | 1.03 (0.85-1.24) | 1.19 (0.89-1.6) | 0.51 |
| rs12554275 | 110765067 | C,G | 0.38 | 0.93 (0.82-1.06) | 0.30 | 0.79 (0.65-0.96) | 0.96 (0.73-1.27) | 0.04 |
| rs77413190 | 110765278 | A,G | 0.01 | 1.09 (0.51-2.33) | 0.82 | #N/A | #N/A | #N/A |
| rs10512369 | 110765359 | G,A | 0.03 | 0.91 (0.63-1.31) | 0.60 | #N/A | #N/A | #N/A |
| rs1416836 | 110766963 | G,A | 0.07 | 0.95 (0.74-1.23) | 0.71 | 0.93 (0.71-1.22) | 1.23 (0.34-4.41) | 0.82 |
| rs9299144 | 110767478 | G,A | 0.50 | 1.02 (0.91-1.16) | 0.70 | 1.07 (0.87-1.33) | 1.05 (0.82-1.34) | 0.82 |
| rs10120885 | 110768664 | C,G | 0.16 | 1.07 (0.9-1.27) | 0.44 | 1.25 (1.02-1.53) | 0.64 (0.37-1.11) | 0.02 |
| rs4979623 | 110769283 | A,G | 0.41 | 1.06 (0.93-1.2) | 0.37 | 1.07 (0.88-1.31) | 1.12 (0.86-1.45) | 0.66 |
| rs9695201 | 110769468 | A,G | 0.41 | 1.07 (0.94-1.22) | 0.29 | 1.1 (0.9-1.34) | 1.14 (0.87-1.48) | 0.54 |
| rs7860780 | 110772665 | G,A | 0.42 | 1.08 (0.95-1.23) | 0.23 | 1.12 (0.92-1.37) | 1.15 (0.89-1.49) | 0.43 |
| rs9695286 | 110773067 | G,A | 0.16 | 1.06 (0.89-1.26) | 0.52 | 1.24 (1.01-1.52) | 0.61 (0.35-1.07) | 0.02 |
| rs77796391 | 110774115 | G,A | 0.01 | 1.06 (0.43-2.58) | 0.91 | #N/A | #N/A | #N/A |
| rs62568532 | 110775973 | G,A | 0.01 | 0.74 (0.43-1.3) | 0.30 | #N/A | #N/A | #N/A |
| rs1339756 | 110776765 | A,G | 0.47 | 0.95 (0.84-1.08) | 0.45 | 0.94 (0.77-1.16) | 0.91 (0.71-1.17) | 0.75 |
| rs10979163 | 110780615 | G,A | 0.07 | 0.91 (0.72-1.16) | 0.46 | 0.9 (0.69-1.17) | 1.01 (0.3-3.33) | 0.72 |
| rs7029094 | 110782565 | A,G | 0.23 | 1.12 (0.96-1.3) | 0.16 | 1.1 (0.91-1.32) | 1.3 (0.85-1.99) | 0.35 |
| rs10512368 | 110783036 | G,A | 0.44 | 1.01 (0.89-1.14) | 0.92 | 0.99 (0.81-1.22) | 1.02 (0.79-1.31) | 0.98 |
| rs10816612 | 110786222 | A,G | 0.45 | 1.03 (0.91-1.17) | 0.64 | 1.14 (0.93-1.4) | 1.04 (0.81-1.34) | 0.41 |
| rs4979625 | 110786533 | A,T | 0.30 | 0.99 (0.86-1.13) | 0.85 | 1 (0.83-1.21) | 0.95 (0.7-1.31) | 0.95 |
| rs7868720 | 110787096 | G,A | 0.43 | 1 (0.89-1.14) | 0.94 | 1.03 (0.84-1.26) | 1 (0.77-1.3) | 0.95 |
| rs1995761 | 110795237 | A,G | 0.31 | 0.99 (0.86-1.13) | 0.85 | 1 (0.83-1.21) | 0.95 (0.7-1.31) | 0.95 |
| rs10759272 | 110795806 | C,G | 0.31 | 0.98 (0.86-1.13) | 0.81 | 0.99 (0.82-1.19) | 0.96 (0.7-1.31) | 0.97 |
| rs11789909 | 110796768 | T,A | 0.01 | 0.82 (0.43-1.57) | 0.55 | #N/A | #N/A | #N/A |
| rs12377186 | 110797181 | A,G | 0.02 | 1.37 (0.89-2.11) | 0.15 | #N/A | #N/A | #N/A |
| rs10979179 | 110805072 | G,A | 0.11 | 1.24 (1.01-1.51) | 0.04 | 1.31 (1.05-1.64) | 1.04 (0.48-2.28) | 0.06 |
| rs7020797 | 110806917 | G,A | 0.19 | 1.11 (0.95-1.31) | 0.19 | 1.16 (0.96-1.41) | 1.07 (0.65-1.75) | 0.32 |
| rs10816621 | 110808571 | G,A | 0.02 | 1.44 (0.84-2.47) | 0.18 | #N/A | #N/A | #N/A |
| rs13301510 | 110809708 | G,A | 0.07 | 0.94 (0.73-1.19) | 0.60 | 0.92 (0.71-1.2) | 1.01 (0.31-3.35) | 0.84 |
| rs6477612 | 110811552 | G,A | 0.31 | 1.02 (0.89-1.17) | 0.80 | 1.09 (0.91-1.32) | 0.95 (0.7-1.3) | 0.54 |
| rs6477613 | 110811614 | G,A | 0.19 | 1.12 (0.95-1.32) | 0.18 | 1.17 (0.96-1.42) | 1.07 (0.65-1.75) | 0.29 |
| rs1318148 | 110814693 | G,C | 0.19 | 1.11 (0.94-1.3) | 0.22 | 1.14 (0.94-1.39) | 1.1 (0.67-1.79) | 0.40 |
| rs892687 | 110814936 | A,C | 0.43 | 0.97 (0.85-1.09) | 0.59 | 1.03 (0.84-1.26) | 0.92 (0.71-1.18) | 0.64 |
| rs7861155 | 110815564 | G,C | 0.03 | 0.73 (0.5-1.08) | 0.12 | #N/A | #N/A | #N/A |
| rs10979182 | 110817020 | A,G | 0.41 | 0.95 (0.84-1.08) | 0.43 | 1 (0.82-1.22) | 0.88 (0.68-1.14) | 0.57 |
| rs4978668 | 110817549 | C,A | 0.36 | 0.96 (0.85-1.1) | 0.59 | 1.04 (0.86-1.26) | 0.88 (0.66-1.17) | 0.50 |
| rs10979183 | 110821050 | G,A | 0.37 | 0.95 (0.83-1.08) | 0.42 | 1.05 (0.87-1.27) | 0.84 (0.64-1.11) | 0.26 |
| rs1878600 | 110821830 | G,A | 0.07 | 0.92 (0.72-1.17) | 0.49 | 0.9 (0.7-1.17) | 1.01 (0.31-3.35) | 0.75 |
| rs1434836 | 110822658 | A,G | 0.39 | 0.94 (0.83-1.07) | 0.38 | 1.06 (0.87-1.29) | 0.84 (0.65-1.09) | 0.20 |
| rs7357711 | 110823759 | A,G | 0.08 | 0.9 (0.71-1.15) | 0.42 | 0.9 (0.7-1.17) | 0.84 (0.24-2.93) | 0.72 |
| rs10979184 | 110825659 | A,G | 0.07 | 1.29 (1-1.66) | 0.04 | 1.37 (1.05-1.8) | 0.83 (0.24-2.89) | 0.07 |
| rs34528190 | 110825688 | A,T | 0.03 | 1.12 (0.75-1.68) | 0.56 | #N/A | #N/A | #N/A |
| rs4978670 | 110826546 | G,A | 0.47 | 1.06 (0.93-1.2) | 0.39 | 1.2 (0.97-1.47) | 1.1 (0.86-1.41) | 0.24 |
| rs10816623 | 110828308 | A,G | 0.12 | 1.23 (1.01-1.5) | 0.04 | 1.28 (1.02-1.6) | 1.13 (0.53-2.45) | 0.09 |
| rs13289116 | 110829012 | A,G | 0.11 | 0.96 (0.79-1.18) | 0.71 | 0.92 (0.74-1.16) | 1.26 (0.54-2.96) | 0.66 |
| rs13291052 | 110832584 | A,G | 0.07 | 0.98 (0.76-1.26) | 0.86 | #N/A | #N/A | #N/A |
| rs13292389 | 110832663 | A,G | 0.01 | 0.69 (0.32-1.51) | 0.35 | #N/A | #N/A | #N/A |
| rs61538103 | 110833526 | C,A | 0.02 | 1.03 (0.64-1.65) | 0.91 | #N/A | #N/A | #N/A |
| rs72745932 | 110834549 | A,G | 0.01 | 1.92 (0.82-4.49) | 0.12 | #N/A | #N/A | #N/A |
| rs10481656 | 110836284 | A,G | 0.50 | 1.05 (0.93-1.18) | 0.46 | 1.22 (0.98-1.51) | 1.1 (0.86-1.4) | 0.18 |
| rs10481657 | 110836385 | G,A | 0.09 | 0.99 (0.79-1.23) | 0.90 | 1.01 (0.79-1.27) | 0.74 (0.22-2.44) | 0.88 |
| rs10481658 | 110836416 | G,A | 0.36 | 0.95 (0.83-1.09) | 0.46 | 1.08 (0.9-1.31) | 0.82 (0.61-1.08) | 0.14 |
| **rs10816625** | **110837073** | **A,G** | **0.01** | **0.86 (0.51-1.44)** | **0.56** | **#N/A** | **#N/A** | **#N/A** |
| **rs13294895** | **110837176** | **G,A** | **0.03** | **0.87 (0.6-1.25)** | **0.44** | **#N/A** | **#N/A** | **#N/A** |
| rs35909461 | 110838269 | A,G | 0.46 | 0.97 (0.86-1.11) | 0.68 | 1 (0.82-1.24) | 0.94 (0.73-1.22) | 0.86 |
| rs7042321 | 110841593 | G,A | 0.43 | 0.9 (0.8-1.03) | 0.12 | 0.85 (0.69-1.04) | 0.83 (0.64-1.08) | 0.22 |
| rs10118883 | 110843927 | A,G | 0.14 | 0.97 (0.81-1.16) | 0.76 | 0.99 (0.8-1.21) | 0.89 (0.47-1.67) | 0.93 |
| rs10979190 | 110845216 | G,A | 0.02 | 0.99 (0.59-1.67) | 0.97 | #N/A | #N/A | #N/A |
| rs10979202 | 110849566 | A,C | 0.01 | 0.93 (0.49-1.78) | 0.83 | #N/A | #N/A | #N/A |
| rs10979204 | 110850362 | G,C | 0.01 | 0.93 (0.49-1.79) | 0.84 | #N/A | #N/A | #N/A |
| rs75004812 | 110853569 | A,G | 0.01 | 1.26 (0.7-2.27) | 0.43 | #N/A | #N/A | #N/A |
| rs12236285 | 110855243 | G,A | 0.01 | 0.93 (0.49-1.79) | 0.84 | #N/A | #N/A | #N/A |
| rs117557721 | 110855537 | G,A | 0.01 | 0.45 (0.18-1.09) | 0.07 | #N/A | #N/A | #N/A |
| rs1434851 | 110857960 | G,A | 0.43 | 1.16 (1.02-1.32) | 0.02 | 1.3 (1.06-1.59) | 1.3 (0.99-1.69) | 0.03 |
| rs1813421 | 110858823 | A,G | 0.36 | 1.26 (1.1-1.44) | 7.27E-04 | 1.38 (1.14-1.67) | 1.48 (1.1-1.98) | 1.52E-03 |
| rs7869753 | 110864852 | A,C | 0.46 | 1.14 (1-1.29) | 0.05 | 1.21 (0.98-1.48) | 1.27 (0.98-1.65) | 0.11 |
| rs78833899 | 110866666 | A,G | 0.01 | 0.93 (0.49-1.79) | 0.84 | #N/A | #N/A | #N/A |
| rs10979209 | 110868281 | G,C | 0.01 | 0.75 (0.42-1.35) | 0.34 | #N/A | #N/A | #N/A |
| rs78417418 | 110871005 | A,G | 0.01 | 1.15 (0.58-2.26) | 0.69 | #N/A | #N/A | #N/A |
| rs10979214 | 110871414 | G,A | 0.22 | 1.04 (0.9-1.22) | 0.59 | 1 (0.83-1.21) | 1.23 (0.8-1.89) | 0.64 |
| rs7042864 | 110872307 | G,A | 0.29 | 1.1 (0.96-1.26) | 0.18 | 1.2 (1-1.45) | 1.06 (0.76-1.48) | 0.16 |
| rs10124038 | 110872527 | A,G | 0.16 | 1.05 (0.88-1.24) | 0.60 | 1.1 (0.89-1.35) | 0.93 (0.55-1.57) | 0.63 |
| rs2043486 | 110873028 | A,G | 0.24 | 1.07 (0.93-1.24) | 0.35 | 1.05 (0.87-1.26) | 1.21 (0.82-1.8) | 0.60 |
| rs62569845 | 110874785 | A,G | 0.24 | 0.99 (0.85-1.15) | 0.89 | 1.05 (0.87-1.26) | 0.87 (0.6-1.28) | 0.65 |
| rs10979217 | 110875895 | A,G | 0.09 | 0.91 (0.73-1.13) | 0.38 | 0.93 (0.73-1.17) | 0.73 (0.3-1.77) | 0.65 |
| rs1999456 | 110877304 | G,C | 0.29 | 0.93 (0.81-1.07) | 0.29 | 0.98 (0.82-1.18) | 0.79 (0.56-1.1) | 0.37 |
| rs1999457 | 110877440 | A,T | 0.17 | 0.96 (0.81-1.13) | 0.63 | 1.02 (0.84-1.25) | 0.75 (0.45-1.23) | 0.49 |
| rs837983 | 110881453 | A,G | 0.31 | 1.01 (0.88-1.16) | 0.84 | 0.96 (0.79-1.15) | 1.12 (0.8-1.55) | 0.64 |
| rs10979219 | 110881731 | A,G | 0.14 | 1.02 (0.85-1.22) | 0.84 | 1.04 (0.84-1.28) | 0.97 (0.53-1.74) | 0.94 |
| rs667052 | 110882390 | A,G | 0.36 | 1.02 (0.9-1.17) | 0.72 | 1.04 (0.86-1.26) | 1.04 (0.78-1.38) | 0.92 |
| rs680138 | 110882980 | A,G | 0.30 | 0.99 (0.86-1.14) | 0.93 | 0.94 (0.78-1.13) | 1.08 (0.77-1.52) | 0.67 |
| rs12376798 | 110883675 | G,A | 0.04 | 1.12 (0.81-1.55) | 0.51 | #N/A | #N/A | #N/A |
| rs630965 | 110885479 | A,G | 0.49 | 1 (0.88-1.14) | 0.97 | 1.01 (0.82-1.25) | 1 (0.78-1.29) | 0.99 |
| rs631475 | 110885650 | A,C | 0.50 | 1 (0.88-1.13) | 0.97 | 0.96 (0.77-1.19) | 1 (0.77-1.28) | 0.89 |
| rs497006 | 110885781 | A,G | 0.48 | 1 (0.88-1.13) | 0.95 | 0.92 (0.74-1.14) | 1 (0.77-1.29) | 0.65 |
| rs519679 | 110885947 | G,C | 0.48 | 0.99 (0.87-1.12) | 0.88 | 0.92 (0.74-1.13) | 0.99 (0.77-1.27) | 0.66 |
| rs520613 | 110886052 | A,G | 0.48 | 0.99 (0.87-1.13) | 0.92 | 0.92 (0.74-1.14) | 1 (0.77-1.29) | 0.66 |
| rs522463 | 110886254 | A,C | 0.48 | 1 (0.88-1.13) | 0.98 | 0.92 (0.74-1.13) | 1 (0.78-1.29) | 0.62 |
| rs12380317 | 110886306 | T,A | 0.11 | 1.09 (0.88-1.33) | 0.43 | 1.16 (0.92-1.45) | 0.74 (0.32-1.73) | 0.34 |
| rs525142 | 110886534 | G,A | 0.50 | 1 (0.88-1.13) | 0.97 | 0.92 (0.74-1.14) | 1 (0.77-1.28) | 0.66 |
| rs62569853 | 110886727 | A,G | 0.08 | 0.85 (0.68-1.07) | 0.16 | 0.82 (0.64-1.05) | 0.98 (0.35-2.73) | 0.29 |
| rs527071 | 110886745 | A,C | 0.50 | 1 (0.88-1.13) | 0.97 | 0.96 (0.77-1.19) | 1 (0.77-1.28) | 0.89 |
| rs548980 | 110886840 | A,G | 0.31 | 1 (0.87-1.15) | 0.98 | 0.93 (0.77-1.12) | 1.12 (0.8-1.56) | 0.49 |
| rs648354 | 110887106 | A,G | 0.48 | 0.97 (0.85-1.1) | 0.61 | 0.87 (0.7-1.08) | 0.94 (0.73-1.23) | 0.41 |
| rs1025573 | 110887338 | G,A | 0.06 | 0.93 (0.72-1.2) | 0.57 | 0.89 (0.68-1.17) | 1.34 (0.38-4.8) | 0.64 |
| rs580018 | 110887934 | A,G | 0.05 | 1.23 (0.93-1.64) | 0.14 | #N/A | #N/A | #N/A |
| rs471467 | 110888113 | A,G | 0.48 | 1 (0.88-1.13) | 0.99 | 0.92 (0.75-1.14) | 1.01 (0.78-1.3) | 0.64 |
| rs472483 | 110888260 | G,A | 0.48 | 1 (0.88-1.13) | 1.00 | 0.92 (0.75-1.14) | 1.01 (0.78-1.3) | 0.66 |
| rs865686 | 110888478 | A,C | 0.48 | 1 (0.88-1.14) | 0.97 | 0.92 (0.75-1.14) | 1.01 (0.79-1.3) | 0.62 |
| rs7026944 | 110891253 | A,G | 0.16 | 1.04 (0.87-1.23) | 0.68 | 1.18 (0.96-1.44) | 0.56 (0.3-1.05) | 0.04 |
| rs7870781 | 110891494 | A,G | 0.45 | 1.03 (0.91-1.18) | 0.61 | 1.12 (0.91-1.37) | 1.05 (0.81-1.36) | 0.56 |
| rs6477617 | 110892030 | G,C | 0.44 | 1.01 (0.89-1.15) | 0.88 | 1.07 (0.87-1.3) | 1 (0.77-1.3) | 0.78 |
| rs510294 | 110892787 | A,T | 0.23 | 0.92 (0.79-1.07) | 0.29 | 0.84 (0.69-1.01) | 1.08 (0.72-1.64) | 0.14 |
| rs7862747 | 110892899 | A,C | 0.23 | 0.93 (0.8-1.08) | 0.32 | 0.83 (0.69-1) | 1.13 (0.74-1.72) | 0.10 |
| rs628931 | 110893030 | G,A | 0.23 | 0.93 (0.8-1.08) | 0.34 | 0.83 (0.69-1.01) | 1.14 (0.75-1.73) | 0.11 |
| rs10979226 | 110893793 | A,T | 0.11 | 1.22 (0.99-1.5) | 0.06 | 1.18 (0.94-1.48) | 1.96 (0.79-4.84) | 0.14 |
| rs10979227 | 110893829 | A,G | 0.11 | 1.22 (0.99-1.5) | 0.06 | 1.18 (0.94-1.48) | 1.96 (0.79-4.84) | 0.14 |
| rs659713 | 110893949 | C,A | 0.23 | 0.94 (0.81-1.09) | 0.41 | 0.84 (0.7-1.02) | 1.16 (0.76-1.76) | 0.12 |
| rs34137313 | 110893956 | A,C | 0.11 | 1.22 (0.99-1.5) | 0.06 | 1.17 (0.93-1.48) | 1.95 (0.79-4.83) | 0.14 |
| rs7047840 | 110894289 | A,G | 0.11 | 1.22 (0.99-1.5) | 0.06 | 1.18 (0.94-1.48) | 1.96 (0.79-4.84) | 0.14 |
| rs7048206 | 110894366 | G,A | 0.11 | 1.22 (0.99-1.5) | 0.06 | 1.18 (0.94-1.48) | 1.96 (0.79-4.84) | 0.14 |
| rs7033853 | 110894500 | A,G | 0.11 | 1.22 (0.99-1.5) | 0.06 | 1.18 (0.94-1.48) | 1.96 (0.79-4.84) | 0.14 |
| rs72745949 | 110894773 | G,A | 0.02 | 0.78 (0.51-1.17) | 0.23 | #N/A | #N/A | #N/A |
| rs10979229 | 110895080 | G,A | 0.11 | 1.22 (0.99-1.5) | 0.06 | 1.18 (0.94-1.48) | 1.96 (0.79-4.85) | 0.14 |
| rs484167 | 110895137 | A,G | 0.17 | 1.07 (0.9-1.26) | 0.44 | 1.07 (0.88-1.3) | 1.14 (0.66-1.97) | 0.74 |
| **rs676256** | **110895353** | **A,G** | **0.24** | **0.95 (0.82-1.1)** | **0.46** | **0.85 (0.7-1.02)** | **1.16 (0.77-1.74)** | **0.13** |
| rs10979230 | 110895509 | C,A | 0.11 | 1.22 (0.99-1.5) | 0.06 | 1.18 (0.94-1.48) | 1.96 (0.79-4.84) | 0.14 |
| rs487887 | 110895527 | A,C | 0.23 | 1.02 (0.88-1.18) | 0.83 | 1.05 (0.87-1.27) | 0.96 (0.64-1.43) | 0.85 |
| rs10979231 | 110895634 | A,G | 0.11 | 1.22 (0.99-1.5) | 0.06 | 1.18 (0.94-1.48) | 1.96 (0.79-4.84) | 0.14 |
| rs10979233 | 110895705 | C,G | 0.11 | 1.22 (0.99-1.5) | 0.06 | 1.18 (0.94-1.48) | 1.96 (0.79-4.85) | 0.14 |
| rs3119744 | 110895863 | C,A | 0.24 | 0.95 (0.82-1.1) | 0.49 | 0.85 (0.71-1.03) | 1.16 (0.77-1.74) | 0.15 |
| rs1434838 | 110896141 | G,A | 0.11 | 1.22 (0.99-1.5) | 0.06 | 1.18 (0.94-1.48) | 1.96 (0.79-4.84) | 0.14 |
| rs1434839 | 110896230 | G,A | 0.11 | 1.21 (0.99-1.5) | 0.07 | 1.18 (0.94-1.49) | 1.81 (0.72-4.53) | 0.16 |
| rs516327 | 110896340 | G,A | 0.22 | 0.98 (0.84-1.15) | 0.84 | 1 (0.83-1.21) | 0.93 (0.6-1.44) | 0.95 |
| rs34793195 | 110896525 | A,G | 0.05 | 1.13 (0.82-1.55) | 0.45 | #N/A | #N/A | #N/A |
| rs10979234 | 110896675 | G,A | 0.11 | 1.22 (0.99-1.5) | 0.06 | 1.18 (0.94-1.49) | 1.96 (0.79-4.86) | 0.13 |
| rs520093 | 110896775 | G,A | 0.34 | 1.07 (0.93-1.22) | 0.35 | 0.98 (0.81-1.18) | 1.25 (0.92-1.69) | 0.27 |
| rs595811 | 110896828 | A,G | 0.35 | 1.05 (0.91-1.19) | 0.52 | 0.98 (0.81-1.18) | 1.16 (0.87-1.56) | 0.50 |
| rs2053148 | 110898048 | C,A | 0.11 | 1.21 (0.99-1.49) | 0.07 | 1.17 (0.93-1.47) | 1.96 (0.79-4.84) | 0.14 |
| rs2117466 | 110898052 | G,A | 0.11 | 1.22 (0.99-1.5) | 0.06 | 1.18 (0.94-1.48) | 1.96 (0.79-4.84) | 0.14 |
| rs10979236 | 110898716 | A,G | 0.11 | 1.23 (1-1.51) | 0.05 | 1.19 (0.95-1.5) | 1.96 (0.79-4.86) | 0.12 |
| rs1836458 | 110898887 | A,C | 0.31 | 0.93 (0.81-1.07) | 0.31 | 0.88 (0.73-1.06) | 0.93 (0.68-1.27) | 0.40 |
| rs10979237 | 110898964 | G,A | 0.11 | 1.22 (1-1.5) | 0.05 | 1.19 (0.95-1.5) | 1.77 (0.78-4.01) | 0.13 |
| rs12379956 | 110899161 | A,G | 0.11 | 1.21 (0.98-1.49) | 0.07 | 1.17 (0.93-1.47) | 1.95 (0.79-4.84) | 0.15 |
| rs12379962 | 110899191 | A,G | 0.11 | 1.22 (1-1.51) | 0.05 | 1.18 (0.94-1.49) | 1.96 (0.79-4.85) | 0.13 |
| rs627610 | 110899291 | C,A | 0.19 | 0.99 (0.84-1.16) | 0.87 | 1.02 (0.84-1.24) | 0.87 (0.54-1.4) | 0.80 |
| rs10979238 | 110899409 | G,A | 0.10 | 1.21 (0.98-1.5) | 0.07 | 1.21 (0.96-1.53) | 1.48 (0.61-3.62) | 0.19 |
| rs10979239 | 110899675 | C,G | 0.11 | 1.2 (0.97-1.47) | 0.09 | 1.17 (0.93-1.47) | 1.7 (0.71-4.06) | 0.21 |
| rs1434840 | 110899937 | C,A | 0.11 | 1.18 (0.96-1.45) | 0.11 | 1.17 (0.93-1.46) | 1.58 (0.65-3.8) | 0.26 |
| rs641002 | 110900018 | G,A | 0.25 | 0.95 (0.82-1.1) | 0.49 | 0.9 (0.75-1.08) | 1.02 (0.7-1.49) | 0.50 |
| rs1434841 | 110900034 | G,A | 0.11 | 1.19 (0.97-1.46) | 0.10 | 1.17 (0.93-1.48) | 1.58 (0.65-3.8) | 0.24 |
| rs1434842 | 110900060 | A,G | 0.11 | 1.18 (0.96-1.45) | 0.11 | 1.17 (0.93-1.46) | 1.58 (0.65-3.8) | 0.27 |
| rs1836459 | 110900115 | G,A | 0.11 | 1.19 (0.97-1.46) | 0.10 | 1.17 (0.93-1.48) | 1.58 (0.65-3.8) | 0.24 |
| rs35338365 | 110900335 | G,A | 0.11 | 1.19 (0.97-1.46) | 0.10 | 1.17 (0.93-1.48) | 1.58 (0.65-3.8) | 0.24 |
| rs10979242 | 110900392 | G,A | 0.11 | 1.19 (0.97-1.46) | 0.10 | 1.17 (0.93-1.48) | 1.58 (0.65-3.8) | 0.24 |
| rs10979243 | 110900418 | G,A | 0.11 | 1.18 (0.96-1.46) | 0.11 | 1.17 (0.93-1.47) | 1.58 (0.65-3.8) | 0.26 |
| rs10979244 | 110900593 | T,A | 0.11 | 1.19 (0.97-1.46) | 0.10 | 1.17 (0.93-1.48) | 1.58 (0.65-3.8) | 0.24 |
| rs10979245 | 110900744 | G,A | 0.11 | 1.19 (0.97-1.46) | 0.10 | 1.17 (0.93-1.48) | 1.58 (0.65-3.8) | 0.24 |
| rs10979246 | 110901072 | G,C | 0.11 | 1.18 (0.96-1.45) | 0.11 | 1.17 (0.93-1.46) | 1.58 (0.65-3.8) | 0.27 |
| rs2571501 | 110902296 | A,T | 0.23 | 1.07 (0.92-1.24) | 0.38 | 1.05 (0.87-1.27) | 1.2 (0.79-1.83) | 0.65 |
| rs10979247 | 110903322 | A,G | 0.11 | 1.18 (0.96-1.45) | 0.11 | 1.16 (0.93-1.46) | 1.57 (0.65-3.79) | 0.27 |
| rs12380087 | 110903380 | C,A | 0.11 | 1.18 (0.96-1.45) | 0.11 | 1.16 (0.93-1.46) | 1.57 (0.65-3.79) | 0.27 |
| rs7853556 | 110903440 | A,G | 0.28 | 0.92 (0.8-1.06) | 0.23 | 0.91 (0.76-1.1) | 0.85 (0.6-1.2) | 0.49 |
| rs10979248 | 110904528 | A,G | 0.05 | 1.1 (0.83-1.45) | 0.51 | #N/A | #N/A | #N/A |
| rs537744 | 110904645 | C,G | 0.27 | 0.94 (0.81-1.08) | 0.37 | 0.86 (0.72-1.04) | 1.02 (0.72-1.46) | 0.26 |
| rs567032 | 110905570 | G,A | 0.33 | 0.99 (0.86-1.13) | 0.86 | 0.93 (0.77-1.13) | 1.05 (0.76-1.43) | 0.68 |
| rs567960 | 110905663 | A,G | 0.48 | 1.01 (0.89-1.15) | 0.83 | 0.99 (0.8-1.23) | 1.03 (0.8-1.33) | 0.95 |
| rs479410 | 110905747 | A,T | 0.20 | 1.01 (0.86-1.19) | 0.87 | 1.05 (0.87-1.28) | 0.9 (0.55-1.45) | 0.75 |
| rs637397 | 110905812 | G,A | 0.42 | 0.88 (0.77-1) | 0.05 | 0.74 (0.61-0.9) | 0.82 (0.63-1.07) | 0.01 |
| rs59244432 | 110906257 | A,G | 0.08 | 0.95 (0.75-1.19) | 0.65 | 0.94 (0.73-1.2) | 1.02 (0.37-2.84) | 0.87 |
| rs651808 | 110906666 | A,G | 0.27 | 1.09 (0.94-1.26) | 0.24 | 1.05 (0.87-1.27) | 1.27 (0.88-1.84) | 0.43 |
| rs651829 | 110906677 | A,G | 0.44 | 1 (0.88-1.14) | 1.00 | 0.86 (0.7-1.05) | 1.04 (0.8-1.36) | 0.17 |
| rs10816634 | 110907407 | A,G | 0.11 | 0.87 (0.71-1.07) | 0.18 | 0.83 (0.66-1.05) | 1.09 (0.43-2.75) | 0.28 |
| rs10979249 | 110907520 | C,A | 0.27 | 0.95 (0.82-1.09) | 0.44 | 0.87 (0.72-1.05) | 1.03 (0.73-1.44) | 0.29 |
| rs10979251 | 110909319 | A,G | 0.11 | 0.9 (0.73-1.1) | 0.29 | 0.85 (0.68-1.06) | 1.3 (0.53-3.17) | 0.28 |
| rs10816635 | 110910044 | G,A | 0.14 | 0.96 (0.8-1.15) | 0.66 | 0.93 (0.75-1.15) | 1.06 (0.59-1.92) | 0.76 |
| rs602325 | 110910506 | T,A | 0.31 | 1.06 (0.92-1.21) | 0.41 | 1.06 (0.88-1.28) | 1.12 (0.82-1.53) | 0.71 |
| rs539270 | 110910792 | A,G | 0.43 | 0.95 (0.83-1.08) | 0.41 | 0.89 (0.73-1.09) | 0.92 (0.71-1.19) | 0.52 |
| rs10512365 | 110911118 | G,A | 0.15 | 0.92 (0.77-1.1) | 0.37 | 0.89 (0.72-1.09) | 1 (0.56-1.79) | 0.53 |
| rs542808 | 110911155 | G,A | 0.25 | 1.07 (0.93-1.24) | 0.36 | 1.02 (0.84-1.23) | 1.28 (0.87-1.88) | 0.45 |
| rs28496063 | 110911169 | G,A | 0.15 | 0.92 (0.77-1.1) | 0.35 | 0.88 (0.72-1.09) | 1 (0.56-1.79) | 0.50 |
| rs28550561 | 110911197 | A,G | 0.16 | 0.97 (0.82-1.15) | 0.73 | 0.95 (0.78-1.16) | 1.03 (0.59-1.8) | 0.87 |
| rs566510 | 110911435 | A,G | 0.27 | 0.96 (0.83-1.1) | 0.54 | 0.87 (0.72-1.04) | 1.1 (0.76-1.58) | 0.22 |
| rs10115125 | 110911908 | G,C | 0.15 | 0.92 (0.77-1.1) | 0.37 | 0.88 (0.71-1.08) | 1.05 (0.58-1.89) | 0.45 |
| rs633082 | 110912805 | C,G | 0.29 | 1.03 (0.9-1.19) | 0.66 | 1 (0.83-1.21) | 1.11 (0.79-1.56) | 0.82 |
| rs634454 | 110913154 | C,A | 0.05 | 1.05 (0.79-1.4) | 0.72 | #N/A | #N/A | #N/A |
| rs635667 | 110913378 | A,G | 0.30 | 1.01 (0.88-1.15) | 0.93 | 0.96 (0.8-1.16) | 1.08 (0.78-1.49) | 0.76 |
| rs677872 | 110914540 | G,A | 0.26 | 0.94 (0.81-1.08) | 0.39 | 0.83 (0.69-1) | 1.13 (0.78-1.65) | 0.08 |
| rs681015 | 110915234 | A,G | 0.22 | 0.96 (0.83-1.13) | 0.64 | 1 (0.83-1.21) | 0.83 (0.54-1.29) | 0.71 |
| rs586559 | 110915881 | A,G | 0.31 | 0.96 (0.84-1.1) | 0.59 | 0.9 (0.75-1.08) | 1.01 (0.74-1.38) | 0.50 |
| rs614851 | 110917627 | A,G | 0.30 | 0.99 (0.86-1.14) | 0.90 | 0.98 (0.82-1.18) | 0.99 (0.71-1.38) | 0.98 |
| rs10816636 | 110918637 | A,C | 0.11 | 0.88 (0.72-1.07) | 0.20 | 0.83 (0.67-1.04) | 1.15 (0.49-2.73) | 0.25 |
| rs34499892 | 110920289 | G,A | 0.01 | 1.29 (0.55-3.06) | 0.55 | #N/A | #N/A | #N/A |
| rs7850979 | 110920305 | G,A | 0.17 | 0.98 (0.82-1.15) | 0.77 | 0.99 (0.81-1.2) | 0.9 (0.52-1.56) | 0.93 |
| rs7866211 | 110920309 | A,G | 0.17 | 0.98 (0.82-1.15) | 0.77 | 0.99 (0.81-1.2) | 0.9 (0.52-1.56) | 0.93 |
| rs113741411 | 110920364 | A,T | 0.49 | 1.06 (0.94-1.21) | 0.34 | 0.94 (0.76-1.17) | 1.14 (0.88-1.47) | 0.25 |
| rs1434843 | 110920807 | G,A | 0.16 | 0.94 (0.79-1.11) | 0.45 | 0.94 (0.77-1.15) | 0.85 (0.47-1.53) | 0.75 |
| rs676083 | 110922092 | G,A | 0.35 | 1 (0.88-1.15) | 0.94 | 0.9 (0.74-1.09) | 1.11 (0.83-1.49) | 0.28 |
| rs10512363 | 110922828 | A,G | 0.09 | 0.89 (0.71-1.11) | 0.31 | 0.89 (0.7-1.14) | 0.8 (0.31-2.04) | 0.59 |
| rs525803 | 110923883 | A,G | 0.24 | 1.01 (0.87-1.17) | 0.88 | 0.9 (0.75-1.09) | 1.36 (0.9-2.06) | 0.13 |
| rs837993 | 110924767 | A,C | 0.24 | 1.04 (0.89-1.21) | 0.62 | 0.94 (0.78-1.13) | 1.38 (0.92-2.09) | 0.19 |
| rs500708 | 110925107 | A,G | 0.24 | 1.05 (0.91-1.22) | 0.51 | 0.94 (0.78-1.13) | 1.47 (0.97-2.23) | 0.10 |
| rs630611 | 110925407 | A,C | 0.09 | 1.04 (0.83-1.3) | 0.75 | 1.02 (0.8-1.31) | 1.21 (0.48-3) | 0.91 |
| rs563471 | 110925919 | A,G | 0.38 | 0.97 (0.86-1.11) | 0.70 | 0.84 (0.69-1.02) | 1.04 (0.79-1.37) | 0.11 |
| rs837994 | 110926669 | C,A | 0.09 | 1.04 (0.83-1.3) | 0.75 | 1 (0.78-1.29) | 1.39 (0.54-3.59) | 0.79 |
| rs116897613 | 110926751 | G,A | 0.01 | 1.09 (0.54-2.2) | 0.82 | #N/A | #N/A | #N/A |
| rs7030021 | 110927072 | C,A | 0.04 | 1.11 (0.81-1.54) | 0.51 | #N/A | #N/A | #N/A |
| rs13290910 | 110927519 | G,A | 0.18 | 0.93 (0.79-1.09) | 0.37 | 0.86 (0.71-1.05) | 1.1 (0.68-1.81) | 0.28 |
| rs510082 | 110927520 | G,A | 0.22 | 1.03 (0.88-1.2) | 0.75 | 0.89 (0.73-1.07) | 1.7 (1.05-2.74) | 0.02 |
| rs511142 | 110927695 | A,G | 0.22 | 1.03 (0.88-1.2) | 0.72 | 0.92 (0.76-1.11) | 1.45 (0.93-2.25) | 0.13 |
| rs671618 | 110927732 | C,A | 0.38 | 0.97 (0.85-1.11) | 0.66 | 0.83 (0.68-1.01) | 1.04 (0.79-1.38) | 0.08 |
| rs687992 | 110929030 | A,G | 0.39 | 0.98 (0.86-1.11) | 0.71 | 0.83 (0.69-1.01) | 1.05 (0.8-1.38) | 0.10 |
| rs7038715 | 110929199 | G,A | 0.26 | 0.98 (0.85-1.13) | 0.82 | 0.93 (0.77-1.13) | 1.06 (0.74-1.51) | 0.69 |
| rs118156642 | 110929289 | A,T | 0.01 | 3.11 (1.14-8.49) | 0.02 | #N/A | #N/A | #N/A |
| rs7027772 | 110929605 | A,G | 0.15 | 0.97 (0.82-1.16) | 0.75 | 0.89 (0.73-1.1) | 1.37 (0.76-2.46) | 0.28 |
| rs593924 | 110929785 | A,T | 0.37 | 1.08 (0.94-1.23) | 0.27 | 0.98 (0.81-1.18) | 1.25 (0.94-1.66) | 0.21 |
| rs573308 | 110929842 | G,C | 0.32 | 1.04 (0.91-1.19) | 0.55 | 0.96 (0.79-1.15) | 1.21 (0.88-1.65) | 0.34 |
| rs527246 | 110932294 | G,A | 0.25 | 1.03 (0.89-1.2) | 0.67 | 0.95 (0.79-1.14) | 1.31 (0.88-1.94) | 0.29 |
| rs626290 | 110932412 | G,A | 0.26 | 0.89 (0.77-1.02) | 0.10 | 0.95 (0.79-1.14) | 0.71 (0.5-1.01) | 0.16 |
| rs477917 | 110936038 | G,C | 0.43 | 0.96 (0.84-1.08) | 0.48 | 0.87 (0.71-1.07) | 0.94 (0.73-1.22) | 0.41 |
| rs509311 | 110937131 | A,C | 0.44 | 0.95 (0.83-1.07) | 0.38 | 0.84 (0.68-1.02) | 0.92 (0.72-1.19) | 0.22 |
| rs10816637 | 110937650 | C,A | 0.44 | 0.93 (0.82-1.06) | 0.29 | 0.82 (0.67-1.01) | 0.9 (0.7-1.17) | 0.17 |
| rs1434844 | 110938877 | T,A | 0.18 | 0.98 (0.83-1.16) | 0.83 | 0.92 (0.76-1.12) | 1.22 (0.72-2.06) | 0.52 |
| rs78952179 | 110939082 | A,G | 0.01 | 1.6 (0.82-3.13) | 0.16 | #N/A | #N/A | #N/A |
| rs10979261 | 110939138 | A,G | 0.17 | 0.94 (0.79-1.11) | 0.46 | 0.88 (0.72-1.07) | 1.17 (0.67-2.05) | 0.35 |
| rs1434846 | 110939308 | A,G | 0.18 | 0.98 (0.83-1.16) | 0.83 | 0.92 (0.76-1.12) | 1.22 (0.72-2.06) | 0.52 |
| rs503238 | 110940013 | G,A | 0.41 | 0.93 (0.82-1.06) | 0.29 | 0.85 (0.7-1.04) | 0.9 (0.7-1.18) | 0.29 |
| rs10979262 | 110940990 | T,A | 0.20 | 1 (0.85-1.18) | 1.00 | 0.96 (0.79-1.17) | 1.16 (0.7-1.92) | 0.76 |
| rs12380632 | 110941095 | A,G | 0.30 | 0.94 (0.82-1.08) | 0.39 | 0.9 (0.75-1.08) | 0.95 (0.68-1.33) | 0.52 |
| rs558016 | 110941382 | G,A | 0.21 | 0.94 (0.81-1.1) | 0.46 | 1.01 (0.84-1.23) | 0.73 (0.47-1.13) | 0.35 |
| rs666303 | 110941390 | G,A | 0.29 | 1.04 (0.91-1.2) | 0.54 | 1.04 (0.86-1.25) | 1.1 (0.79-1.53) | 0.82 |
| rs77889939 | 110942176 | T,A | 0.22 | 1.03 (0.89-1.19) | 0.72 | 1.04 (0.86-1.26) | 1.03 (0.7-1.53) | 0.93 |
| rs683466 | 110942879 | G,A | 0.10 | 1.13 (0.92-1.39) | 0.25 | 1.04 (0.81-1.32) | 1.99 (0.9-4.39) | 0.21 |
| rs10979263 | 110943815 | A,G | 0.38 | 0.97 (0.85-1.1) | 0.60 | 0.92 (0.76-1.12) | 0.96 (0.73-1.27) | 0.70 |
| rs10979264 | 110944986 | G,A | 0.01 | 1.14 (0.67-1.94) | 0.64 | #N/A | #N/A | #N/A |
| rs607019 | 110945296 | G,A | 0.10 | 1.16 (0.94-1.43) | 0.16 | 1.07 (0.84-1.35) | 2.34 (0.97-5.61) | 0.12 |
| rs607056 | 110945330 | G,A | 0.11 | 1.13 (0.93-1.37) | 0.22 | 1.08 (0.84-1.38) | 1.44 (0.81-2.53) | 0.40 |
| rs10759273 | 110946296 | A,G | 0.33 | 0.99 (0.87-1.14) | 0.94 | 0.95 (0.79-1.15) | 1.03 (0.77-1.38) | 0.81 |
| rs559836 | 110946380 | G,A | 0.10 | 1.16 (0.94-1.43) | 0.16 | 1.07 (0.84-1.35) | 2.34 (0.97-5.61) | 0.12 |
| rs10816640 | 110946857 | G,A | 0.12 | 1.01 (0.83-1.22) | 0.93 | 1.01 (0.8-1.26) | 1.04 (0.53-2.03) | 0.99 |
| rs553333 | 110947155 | G,A | 0.35 | 1.04 (0.92-1.19) | 0.53 | 0.95 (0.79-1.15) | 1.17 (0.88-1.56) | 0.36 |
| rs864145 | 110948023 | G,C | 0.10 | 1.16 (0.94-1.43) | 0.18 | 1.06 (0.83-1.35) | 2.33 (0.97-5.6) | 0.13 |
| rs7875207 | 110948812 | G,A | 0.01 | 0.49 (0.2-1.2) | 0.11 | #N/A | #N/A | #N/A |
| rs10816642 | 110950488 | A,G | 0.13 | 1.05 (0.87-1.27) | 0.60 | 1.09 (0.88-1.35) | 0.93 (0.48-1.79) | 0.71 |
| rs7022714 | 110950572 | G,A | 0.20 | 1.04 (0.88-1.22) | 0.64 | 1.06 (0.87-1.29) | 1.03 (0.65-1.62) | 0.86 |
| rs13295544 | 110953139 | G,A | 0.37 | 1.07 (0.94-1.22) | 0.32 | 1.17 (0.96-1.42) | 1.07 (0.81-1.42) | 0.28 |
| rs28450518 | 110953584 | T,A | 0.40 | 0.87 (0.77-0.99) | 0.03 | 0.96 (0.79-1.17) | 0.73 (0.56-0.94) | 0.04 |
| rs669329 | 110954331 | G,A | 0.22 | 1.07 (0.92-1.24) | 0.41 | 1.11 (0.91-1.34) | 1.04 (0.69-1.57) | 0.59 |
| rs550891 | 110954663 | C,A | 0.18 | 0.94 (0.8-1.1) | 0.42 | 0.92 (0.75-1.12) | 0.92 (0.59-1.44) | 0.69 |
| rs59860864 | 110954925 | C,A | 0.31 | 1.09 (0.96-1.25) | 0.19 | 1 (0.83-1.21) | 1.32 (0.97-1.8) | 0.18 |
| rs13287026 | 110955043 | G,A | 0.05 | 1.06 (0.79-1.42) | 0.71 | #N/A | #N/A | #N/A |
| rs556399 | 110955288 | G,A | 0.21 | 1.05 (0.9-1.22) | 0.52 | 1.11 (0.91-1.35) | 0.97 (0.64-1.47) | 0.54 |
| rs34979687 | 110955816 | A,G | 0.31 | 1.09 (0.96-1.25) | 0.19 | 1.03 (0.85-1.24) | 1.29 (0.94-1.75) | 0.28 |
| rs638978 | 110956479 | G,A | 0.10 | 1.06 (0.85-1.32) | 0.62 | 1.02 (0.8-1.3) | 1.52 (0.55-4.24) | 0.71 |
| rs75802750 | 110956500 | G,A | 0.11 | 1.07 (0.88-1.31) | 0.49 | 1.13 (0.9-1.42) | 0.8 (0.36-1.78) | 0.47 |
| rs625647 | 110957231 | A,C | 0.22 | 1.03 (0.89-1.2) | 0.69 | 1.14 (0.94-1.38) | 0.85 (0.56-1.28) | 0.25 |
| rs507584 | 110957372 | T,A | 0.09 | 1.1 (0.88-1.38) | 0.38 | 1.08 (0.84-1.38) | 1.54 (0.55-4.3) | 0.60 |
| rs538688 | 110958479 | A,C | 0.09 | 1.11 (0.89-1.38) | 0.37 | 1.07 (0.84-1.36) | 1.67 (0.61-4.57) | 0.53 |
| rs10979270 | 110959027 | A,G | 0.02 | 0.76 (0.45-1.28) | 0.30 | #N/A | #N/A | #N/A |
| rs569795 | 110959560 | G,A | 0.25 | 1.02 (0.88-1.18) | 0.77 | 1.09 (0.9-1.31) | 0.92 (0.63-1.34) | 0.55 |
| rs569859 | 110959588 | A,G | 0.27 | 1 (0.87-1.16) | 0.96 | 1.04 (0.86-1.25) | 0.95 (0.67-1.35) | 0.86 |
| rs581561 | 110960080 | C,A | 0.16 | 0.96 (0.81-1.14) | 0.63 | 0.93 (0.76-1.14) | 1.04 (0.61-1.77) | 0.75 |
| rs507242 | 110969263 | A,G | 0.12 | 1.02 (0.83-1.24) | 0.88 | 0.98 (0.79-1.23) | 1.31 (0.57-2.98) | 0.80 |
| rs838013 | 110969574 | A,G | 0.09 | 1.12 (0.89-1.39) | 0.33 | 1.08 (0.84-1.37) | 1.71 (0.63-4.68) | 0.49 |
| rs838012 | 110969777 | G,A | 0.11 | 1.14 (0.93-1.4) | 0.19 | 1.1 (0.87-1.37) | 1.79 (0.79-4.06) | 0.29 |
| rs10816648 | 110971140 | A,G | 0.04 | 1.12 (0.8-1.56) | 0.50 | #N/A | #N/A | #N/A |
| rs35517812 | 110971386 | G,A | 0.36 | 1.06 (0.93-1.21) | 0.39 | 1.04 (0.86-1.26) | 1.14 (0.86-1.51) | 0.66 |
| rs485572 | 110972062 | C,G | 0.10 | 1.11 (0.89-1.39) | 0.37 | 1.07 (0.84-1.36) | 1.82 (0.61-5.37) | 0.49 |
| rs539221 | 110972292 | A,C | 0.33 | 0.95 (0.83-1.08) | 0.44 | 1 (0.82-1.21) | 0.86 (0.64-1.16) | 0.59 |
| rs10979274 | 110973879 | A,G | 0.06 | 0.91 (0.7-1.18) | 0.48 | 0.92 (0.68-1.24) | 0.77 (0.28-2.14) | 0.76 |
| rs609982 | 110974043 | C,G | 0.25 | 1.04 (0.9-1.21) | 0.57 | 1.09 (0.9-1.31) | 0.99 (0.67-1.46) | 0.66 |
| rs73998 | 110974433 | C,A | 0.09 | 1.12 (0.9-1.41) | 0.31 | 1.11 (0.87-1.42) | 1.39 (0.49-3.96) | 0.58 |
| rs10979275 | 110975315 | G,A | 0.02 | 1.08 (0.68-1.71) | 0.75 | #N/A | #N/A | #N/A |
| rs548698 | 110975463 | C,A | 0.09 | 1.11 (0.89-1.39) | 0.37 | 1.07 (0.84-1.36) | 1.84 (0.62-5.46) | 0.47 |
| rs838001 | 110975498 | A,C | 0.44 | 1.07 (0.94-1.21) | 0.29 | 1.27 (1.04-1.56) | 1.09 (0.84-1.41) | 0.06 |
| rs838002 | 110975878 | A,G | 0.11 | 1.06 (0.86-1.31) | 0.57 | 1.09 (0.86-1.37) | 0.95 (0.41-2.18) | 0.76 |
| rs10979276 | 110975982 | A,G | 0.11 | 1.06 (0.86-1.31) | 0.57 | 1.09 (0.86-1.37) | 0.95 (0.41-2.18) | 0.76 |
| rs73519684 | 110976133 | A,C | 0.35 | 1.04 (0.92-1.19) | 0.52 | 1.13 (0.93-1.36) | 1.02 (0.77-1.36) | 0.45 |
| rs498727 | 110976981 | A,G | 0.45 | 1.07 (0.95-1.22) | 0.27 | 1.34 (1.09-1.64) | 1.09 (0.85-1.41) | 0.01 |
| rs501345 | 110977231 | A,G | 0.45 | 0.93 (0.82-1.05) | 0.24 | 1 (0.82-1.23) | 0.85 (0.66-1.09) | 0.32 |
| rs656048 | 110977391 | A,G | 0.40 | 1.11 (0.97-1.26) | 0.12 | 1.29 (1.06-1.57) | 1.13 (0.87-1.48) | 0.04 |
| rs7026619 | 110978841 | A,C | 0.37 | 0.98 (0.86-1.12) | 0.77 | 0.9 (0.75-1.1) | 1.02 (0.77-1.34) | 0.51 |
| rs672586 | 110978859 | A,G | 0.34 | 1.05 (0.92-1.19) | 0.51 | 1.13 (0.94-1.37) | 1.02 (0.76-1.36) | 0.41 |
| rs4246873 | 110980594 | G,A | 0.33 | 1.03 (0.9-1.18) | 0.71 | 0.98 (0.81-1.18) | 1.11 (0.81-1.52) | 0.72 |
| rs62567152 | 110982252 | G,A | 0.13 | 1.02 (0.85-1.24) | 0.80 | 0.94 (0.76-1.16) | 2.43 (0.94-6.27) | 0.12 |
| rs519144 | 110986403 | G,A | 0.17 | 1.01 (0.85-1.19) | 0.93 | 1.09 (0.89-1.33) | 0.72 (0.41-1.26) | 0.32 |
| rs10979283 | 110987236 | C,A | 0.08 | 1.01 (0.79-1.28) | 0.94 | #N/A | #N/A | #N/A |
| rs7043432 | 110988346 | A,G | 0.38 | 0.89 (0.78-1.02) | 0.09 | 0.85 (0.7-1.03) | 0.82 (0.62-1.08) | 0.18 |
| rs837999 | 110989319 | C,A | 0.11 | 1.17 (0.95-1.44) | 0.14 | 1.12 (0.89-1.4) | 2.11 (0.8-5.55) | 0.21 |
| rs10979285 | 110989866 | G,A | 0.02 | 1.3 (0.78-2.16) | 0.32 | #N/A | #N/A | #N/A |
| rs868895 | 110989880 | C,A | 0.22 | 1.07 (0.92-1.25) | 0.38 | 1.11 (0.92-1.34) | 1.05 (0.68-1.61) | 0.57 |
| rs1888913 | 110990323 | C,A | 0.34 | 1.14 (0.99-1.31) | 0.06 | 1.16 (0.96-1.41) | 1.27 (0.94-1.72) | 0.17 |
| rs75594954 | 110999079 | A,G | 0.05 | 0.95 (0.72-1.25) | 0.71 | #N/A | #N/A | #N/A |
| rs35034713 | 111000704 | A,G | 0.08 | 1.17 (0.93-1.48) | 0.19 | 1.26 (0.98-1.62) | 0.54 (0.16-1.87) | 0.11 |
| rs7847854 | 111002172 | G,A | 0.47 | 1 (0.88-1.13) | 0.98 | 0.87 (0.71-1.08) | 1.01 (0.79-1.3) | 0.30 |
| rs74938381 | 111002999 | C,A | 0.01 | 0.84 (0.39-1.84) | 0.67 | #N/A | #N/A | #N/A |
| rs1360533 | 111004738 | A,G | 0.38 | 0.94 (0.82-1.07) | 0.33 | 0.82 (0.68-1) | 0.95 (0.72-1.26) | 0.12 |
| rs1556472 | 111005974 | T,A | 0.05 | 0.95 (0.71-1.28) | 0.74 | #N/A | #N/A | #N/A |
| rs4644328 | 111008699 | A,G | 0.22 | 0.9 (0.78-1.05) | 0.17 | 0.93 (0.77-1.13) | 0.75 (0.51-1.11) | 0.33 |
| rs4382542 | 111008909 | G,A | 0.22 | 0.9 (0.77-1.04) | 0.16 | 0.94 (0.78-1.14) | 0.73 (0.49-1.09) | 0.28 |
| rs1332299 | 111012416 | A,G | 0.39 | 0.98 (0.86-1.11) | 0.73 | 1 (0.82-1.22) | 0.94 (0.72-1.23) | 0.89 |
| rs1332300 | 111012582 | G,C | 0.28 | 0.92 (0.8-1.06) | 0.24 | 0.93 (0.77-1.12) | 0.84 (0.6-1.17) | 0.51 |
| rs10979291 | 111014868 | G,A | 0.10 | 1.03 (0.83-1.28) | 0.79 | 1.09 (0.87-1.38) | 0.56 (0.2-1.59) | 0.40 |
| rs1332302 | 111015811 | T,A | 0.34 | 1.1 (0.96-1.26) | 0.16 | 1.05 (0.87-1.27) | 1.28 (0.95-1.72) | 0.28 |
| rs7034951 | 111016395 | A,G | 0.28 | 0.93 (0.81-1.06) | 0.28 | 0.96 (0.79-1.15) | 0.81 (0.58-1.14) | 0.48 |
| rs1412433 | 111017507 | A,G | 0.29 | 0.97 (0.84-1.11) | 0.62 | 0.95 (0.79-1.15) | 0.95 (0.68-1.32) | 0.86 |
| rs2900448 | 111017606 | A,G | 0.02 | 0.76 (0.49-1.18) | 0.22 | #N/A | #N/A | #N/A |
| rs7043626 | 111018343 | A,C | 0.38 | 0.92 (0.81-1.05) | 0.23 | 0.81 (0.67-0.98) | 0.93 (0.7-1.23) | 0.10 |
| rs1537286 | 111021175 | G,A | 0.22 | 0.9 (0.77-1.04) | 0.15 | 0.95 (0.79-1.15) | 0.7 (0.47-1.05) | 0.22 |
| rs7030526 | 111022525 | A,G | 0.48 | 0.86 (0.76-0.98) | 0.02 | 0.91 (0.74-1.12) | 0.74 (0.58-0.96) | 0.06 |
| rs10739255 | 111023899 | G,A | 0.27 | 1.2 (1.04-1.39) | 0.01 | 1.25 (1.04-1.51) | 1.32 (0.92-1.91) | 0.04 |
| rs10739256 | 111024123 | A,G | 0.28 | 1.2 (1.04-1.38) | 0.01 | 1.22 (1.01-1.47) | 1.38 (0.96-1.98) | 0.05 |
| rs10759275 | 111024438 | A,C | 0.28 | 1.19 (1.03-1.37) | 0.02 | 1.22 (1.01-1.47) | 1.35 (0.94-1.94) | 0.06 |
| rs10979297 | 111024683 | T,A | 0.08 | 1.36 (1.07-1.73) | 0.01 | 1.32 (1.02-1.71) | 2.78 (0.74-10.36) | 0.03 |
| rs1758617 | 111025649 | G,A | 0.26 | 1.19 (1.03-1.38) | 0.02 | 1.22 (1.02-1.48) | 1.35 (0.93-1.97) | 0.05 |
| rs2778252 | 111026158 | A,G | 0.13 | 0.9 (0.75-1.09) | 0.29 | 0.96 (0.78-1.2) | 0.59 (0.3-1.14) | 0.28 |
| rs13283710 | 111026582 | G,A | 0.15 | 0.87 (0.73-1.05) | 0.14 | 0.95 (0.78-1.17) | 0.42 (0.2-0.88) | 0.06 |
| rs3117888 | 111027827 | G,A | 0.42 | 1.08 (0.95-1.23) | 0.24 | 1.06 (0.87-1.3) | 1.17 (0.9-1.53) | 0.50 |
| rs2778253 | 111028359 | C,G | 0.25 | 1.21 (1.05-1.41) | 0.01 | 1.23 (1.02-1.48) | 1.42 (0.96-2.09) | 0.04 |
| rs2778254 | 111028362 | C,A | 0.25 | 1.21 (1.05-1.41) | 0.01 | 1.23 (1.02-1.48) | 1.42 (0.96-2.09) | 0.04 |
| rs3117889 | 111028604 | G,C | 0.32 | 1.12 (0.98-1.29) | 0.09 | 1.1 (0.91-1.33) | 1.3 (0.95-1.78) | 0.23 |
| rs3119743 | 111028887 | A,T | 0.31 | 1.12 (0.97-1.28) | 0.11 | 1.09 (0.9-1.31) | 1.28 (0.94-1.76) | 0.27 |
| rs1330591 | 111029103 | A,G | 0.31 | 1.01 (0.88-1.16) | 0.86 | 0.93 (0.77-1.12) | 1.13 (0.83-1.55) | 0.44 |
| rs2482603 | 111029969 | A,G | 0.18 | 0.87 (0.73-1.02) | 0.09 | 0.92 (0.76-1.12) | 0.58 (0.34-0.98) | 0.11 |
| rs3117890 | 111031148 | A,G | 0.49 | 1.04 (0.92-1.18) | 0.54 | 0.97 (0.78-1.21) | 1.09 (0.84-1.4) | 0.62 |
| rs645119 | 111031550 | A,G | 0.13 | 0.89 (0.73-1.08) | 0.23 | 0.97 (0.78-1.2) | 0.51 (0.25-1.02) | 0.15 |
| rs2778255 | 111032311 | G,A | 0.20 | 1.25 (1.06-1.46) | 6.74E-03 | 1.21 (0.99-1.46) | 1.72 (1.07-2.78) | 0.02 |
| rs1323397 | 111034788 | A,G | 0.06 | 1.49 (1.12-1.99) | 5.88E-03 | #N/A | #N/A | #N/A |
| rs9299149 | 111034825 | C,A | 0.23 | 0.89 (0.77-1.03) | 0.12 | 0.94 (0.78-1.14) | 0.69 (0.47-1.03) | 0.18 |
| rs7039993 | 111034920 | G,C | 0.45 | 0.97 (0.86-1.1) | 0.65 | 1.08 (0.88-1.33) | 0.92 (0.72-1.19) | 0.39 |
| rs10759277 | 111035445 | A,G | 0.46 | 0.96 (0.85-1.09) | 0.57 | 1.12 (0.91-1.38) | 0.91 (0.71-1.17) | 0.18 |
| rs1575449 | 111037191 | A,G | 0.15 | 1.14 (0.95-1.37) | 0.15 | 1.14 (0.93-1.4) | 1.31 (0.68-2.52) | 0.36 |
| rs1323374 | 111037829 | T,A | 0.45 | 0.93 (0.81-1.05) | 0.23 | 0.92 (0.75-1.13) | 0.86 (0.66-1.11) | 0.48 |
| rs10979300 | 111040235 | G,A | 0.12 | 1.12 (0.92-1.36) | 0.25 | 1.12 (0.9-1.39) | 1.24 (0.58-2.66) | 0.52 |
| rs1323376 | 111042771 | G,A | 0.23 | 0.91 (0.78-1.05) | 0.20 | 0.94 (0.78-1.14) | 0.77 (0.52-1.13) | 0.37 |
| rs1323377 | 111043009 | A,G | 0.23 | 0.91 (0.79-1.06) | 0.22 | 0.94 (0.78-1.14) | 0.78 (0.53-1.15) | 0.43 |
| rs10979301 | 111044933 | A,G | 0.19 | 0.9 (0.77-1.06) | 0.20 | 0.92 (0.76-1.12) | 0.76 (0.48-1.21) | 0.41 |
| rs12552629 | 111047312 | A,G | 0.46 | 0.89 (0.79-1.01) | 0.06 | 0.89 (0.72-1.09) | 0.79 (0.62-1.02) | 0.18 |
| rs7852572 | 111047463 | G,A | 0.08 | 1.35 (1.06-1.72) | 0.01 | 1.29 (1-1.68) | 2.99 (0.82-10.98) | 0.04 |
| rs72747953 | 111050043 | G,A | 0.01 | 1.78 (0.79-4) | 0.15 | #N/A | #N/A | #N/A |
| rs7021201 | 111050366 | A,G | 0.19 | 1.19 (1.01-1.4) | 0.03 | 1.17 (0.97-1.43) | 1.5 (0.92-2.44) | 0.10 |
| rs10979302 | 111050704 | A,C | 0.01 | 0.94 (0.53-1.69) | 0.84 | #N/A | #N/A | #N/A |
| rs12353336 | 111050810 | G,A | 0.35 | 0.9 (0.79-1.03) | 0.12 | 0.87 (0.72-1.06) | 0.84 (0.63-1.11) | 0.27 |
| rs10117055 | 111051418 | G,A | 0.18 | 1.2 (1.01-1.42) | 0.03 | 1.22 (1-1.49) | 1.34 (0.8-2.25) | 0.10 |
| rs10114572 | 111051611 | A,G | 0.18 | 1.2 (1.02-1.42) | 0.03 | 1.23 (1.01-1.5) | 1.35 (0.81-2.25) | 0.09 |
| rs10117687 | 111051626 | T,A | 0.34 | 0.89 (0.78-1.02) | 0.09 | 0.83 (0.69-1.01) | 0.85 (0.64-1.12) | 0.14 |
| rs73653749 | 111051751 | A,G | 0.13 | 1.22 (1.01-1.46) | 0.04 | 1.22 (0.99-1.51) | 1.43 (0.72-2.84) | 0.12 |
| rs4978678 | 111053537 | A,C | 0.29 | 0.89 (0.77-1.02) | 0.09 | 0.88 (0.73-1.06) | 0.8 (0.58-1.11) | 0.23 |
| rs56125520 | 111054295 | A,C | 0.06 | 0.93 (0.71-1.21) | 0.60 | #N/A | #N/A | #N/A |
| rs7046291 | 111054302 | G,C | 0.43 | 1.01 (0.89-1.14) | 0.91 | 1.08 (0.88-1.32) | 0.99 (0.77-1.28) | 0.68 |
| rs10979304 | 111055670 | A,G | 0.01 | 0.94 (0.53-1.69) | 0.84 | #N/A | #N/A | #N/A |
| rs55896475 | 111056782 | G,A | 0.16 | 0.99 (0.83-1.17) | 0.89 | 0.99 (0.81-1.21) | 0.98 (0.54-1.77) | 0.99 |
| rs13300950 | 111057524 | A,G | 0.07 | 0.87 (0.67-1.12) | 0.27 | #N/A | #N/A | #N/A |
| rs77922938 | 111060196 | G,A | 0.01 | 0.78 (0.37-1.62) | 0.50 | #N/A | #N/A | #N/A |
| rs1323380 | 111060921 | C,A | 0.14 | 0.96 (0.8-1.15) | 0.67 | 1.06 (0.86-1.31) | 0.52 (0.26-1.05) | 0.15 |
| rs1885973 | 111061706 | T,A | 0.45 | 1.04 (0.91-1.18) | 0.56 | 1.03 (0.84-1.27) | 1.08 (0.83-1.4) | 0.84 |
| rs10979312 | 111064186 | A,C | 0.20 | 0.9 (0.77-1.06) | 0.20 | 0.96 (0.79-1.17) | 0.7 (0.45-1.08) | 0.26 |
| rs1323381 | 111064438 | G,A | 0.14 | 0.95 (0.79-1.14) | 0.60 | 1.06 (0.86-1.3) | 0.43 (0.2-0.92) | 0.06 |
| rs1407849 | 111064598 | A,T | 0.45 | 0.98 (0.86-1.11) | 0.71 | 1.07 (0.87-1.32) | 0.93 (0.72-1.21) | 0.48 |
| rs1407850 | 111064801 | A,G | 0.15 | 1.22 (1.02-1.45) | 0.03 | 1.22 (0.99-1.5) | 1.48 (0.8-2.73) | 0.10 |
| rs1323383 | 111065424 | A,G | 0.12 | 1.43 (1.16-1.75) | 5.86E-04 | 1.38 (1.11-1.73) | 2.72 (1.06-6.98) | 2.13E-03 |
| rs117936512 | 111066476 | C,A | 0.15 | 1.24 (1.03-1.49) | 0.02 | 1.23 (1-1.52) | 1.58 (0.84-2.96) | 0.07 |
| rs17543333 | 111066833 | A,C | 0.15 | 0.96 (0.8-1.14) | 0.62 | 1.05 (0.86-1.29) | 0.53 (0.28-1.03) | 0.13 |
| rs16912868 | 111067924 | A,G | 0.50 | 0.96 (0.85-1.09) | 0.57 | 0.98 (0.79-1.22) | 0.93 (0.72-1.2) | 0.83 |
| rs116991065 | 111070042 | A,G | 0.17 | 0.94 (0.8-1.11) | 0.47 | 1.02 (0.84-1.25) | 0.65 (0.38-1.1) | 0.25 |
| rs2093680 | 111070724 | A,G | 0.36 | 1.12 (0.98-1.28) | 0.09 | 1.04 (0.86-1.26) | 1.33 (1-1.77) | 0.14 |
| rs79831337 | 111070857 | A,G | 0.08 | 1.3 (1.02-1.66) | 0.03 | 1.25 (0.97-1.62) | 2.74 (0.74-10.18) | 0.07 |
| rs1407851 | 111071459 | G,C | 0.12 | 1.31 (1.07-1.59) | 8.45E-03 | 1.2 (0.96-1.51) | 2.82 (1.26-6.35) | 0.01 |
| rs56020002 | 111071733 | G,A | 0.15 | 0.9 (0.76-1.08) | 0.25 | 1.01 (0.83-1.23) | 0.43 (0.22-0.84) | 0.04 |
| rs10979314 | 111072429 | G,A | 0.02 | 1.16 (0.77-1.74) | 0.48 | #N/A | #N/A | #N/A |
| rs10979315 | 111072586 | G,A | 0.48 | 1.18 (1.04-1.33) | 9.03E-03 | 1.33 (1.08-1.64) | 1.37 (1.07-1.76) | 0.01 |
| rs12349823 | 111072668 | A,G | 0.49 | 1.19 (1.05-1.34) | 6.11E-03 | 1.3 (1.05-1.61) | 1.4 (1.1-1.8) | 0.01 |
| rs12336489 | 111073103 | A,G | 0.38 | 0.87 (0.77-0.99) | 0.03 | 0.88 (0.72-1.06) | 0.75 (0.58-0.98) | 0.09 |
| rs12336573 | 111073347 | A,G | 0.36 | 0.88 (0.77-1) | 0.04 | 0.87 (0.72-1.05) | 0.77 (0.59-1.02) | 0.13 |
| rs10283642 | 111074243 | G,A | 0.19 | 0.99 (0.84-1.16) | 0.89 | 1.01 (0.83-1.23) | 0.9 (0.55-1.48) | 0.90 |
| rs10739257 | 111074457 | C,A | 0.41 | 0.9 (0.79-1.03) | 0.13 | 0.97 (0.79-1.18) | 0.79 (0.6-1.04) | 0.21 |
| rs62570194 | 111075953 | A,G | 0.03 | 0.82 (0.54-1.25) | 0.36 | #N/A | #N/A | #N/A |
| rs7033858 | 111077461 | G,A | 0.27 | 1.04 (0.9-1.2) | 0.59 | 1.07 (0.89-1.29) | 1.03 (0.72-1.46) | 0.77 |
| rs1923964 | 111081694 | G,C | 0.41 | 1.03 (0.9-1.17) | 0.67 | 1.08 (0.89-1.32) | 1.03 (0.79-1.34) | 0.72 |
| rs4978345 | 111082271 | C,A | 0.20 | 1.08 (0.92-1.27) | 0.33 | 1 (0.83-1.22) | 1.5 (0.93-2.44) | 0.24 |
| rs62570196 | 111086170 | A,G | 0.02 | 0.97 (0.58-1.6) | 0.89 | #N/A | #N/A | #N/A |
| rs62570197 | 111086510 | G,A | 0.02 | 0.98 (0.63-1.51) | 0.92 | #N/A | #N/A | #N/A |
| rs77284504 | 111086758 | A,T | 0.06 | 0.97 (0.74-1.28) | 0.85 | #N/A | #N/A | #N/A |
| rs10816659 | 111092798 | A,G | 0.50 | 1.07 (0.94-1.22) | 0.28 | 1.13 (0.91-1.4) | 1.15 (0.89-1.48) | 0.48 |
| rs2417854 | 111095847 | C,G | 0.47 | 0.94 (0.83-1.07) | 0.34 | 0.93 (0.75-1.15) | 0.89 (0.69-1.14) | 0.63 |
| rs1323389 | 111098510 | G,A | 0.46 | 1.07 (0.94-1.21) | 0.31 | 1.12 (0.91-1.38) | 1.13 (0.87-1.46) | 0.50 |
| rs16936302 | 111100826 | A,G | 0.06 | 1.01 (0.77-1.32) | 0.95 | #N/A | #N/A | #N/A |
| rs12550936 | 111106217 | C,A | 0.33 | 0.95 (0.82-1.09) | 0.46 | 0.89 (0.74-1.08) | 0.97 (0.7-1.34) | 0.50 |

**Supplemental Table 3. Association statistics and effect estimates for 9q31.2 CVS using imputed data.**

| **Locus** | **Coordinate** | **r^2^ with rs676256** | **OR** | **95% CI** | **P-value** |
| --- | --- | --- | --- | --- | --- |
| rs837983 | 110881453 | 0.95 | 0.90 | 0.88-0.92 | 1.85 x 10^-25^ |
| rs680138 | 110882980 | 0.96 | 0.90 | 0.88-0.92 | 4.77 x 10^-25^ |
| rs630965 | 110885479 | 0.96 | 0.90 | 0.88-0.92 | 1.67 x 10^-25^ |
| rs631475 | 110885650 | 0.96 | 0.90 | 0.88-0.92 | 1.73 x 10^-25^ |
| rs497006 | 110885781 | 0.96 | 0.90 | 0.88-0.92 | 4.30 x 10^-25^ |
| rs519679 | 110885947 | 0.96 | 0.90 | 0.88-0.92 | 5.31 x 10^-25^ |
| rs520613 | 110886052 | 0.96 | 0.90 | 0.88-0.92 | 6.18 x 10^-25^ |
| rs522463 | 110886254 | 0.96 | 0.90 | 0.88-0.92 | 5.38 x 10^-25^ |
| rs525142 | 110886534 | 0.96 | 0.90 | 0.88-0.92 | 6.55 x 10^-25^ |
| rs527071 | 110886745 | 0.96 | 0.90 | 0.88-0.92 | 3.92 x 10^-25^ |
| rs548980 | 110886840 | 0.97 | 0.90 | 0.88-0.92 | 1.86 x 10^-25^ |
| rs648354 | 110887106 | 0.96 | 0.90 | 0.88-0.92 | 6.09 x 10^-25^ |
| rs662694 | 110887996 | 0.96 | 0.90 | 0.88-0.92 | 4.49 x 10^-25^ |
| rs471467 | 110888113 | 0.97 | 0.90 | 0.88-0.92 | 2.63 x 10^-25^ |
| rs472483 | 110888260 | 0.96 | 0.90 | 0.88-0.92 | 6.88 x 10^-25^ |
| rs865686 | 110888478 | 0.96 | 0.90 | 0.88-0.92 | 3.91 x 10^-25^ |
| rs857609 | 110888677 | 0.95 | 0.90 | 0.88-0.92 | 2.56 x 10^-25^ |
| rs857610 | 110888809 | 0.96 | 0.90 | 0.88-0.92 | 4.04 x 10^-25^ |
| rs1618654 | 110888866 | 0.94 | 0.90 | 0.88-0.92 | 2.67 x 10^-25^ |
| rs510294 | 110892787 | 0.98 | 0.90 | 0.88-0.92 | 1.01 x 10^-24^ |
| rs7862747 | 110892899 | 0.99 | 0.90 | 0.88-0.92 | 2.67 x 10^-25^ |
| rs628931 | 110893030 | 0.99 | 0.90 | 0.88-0.92 | 1.08 x 10^-24^ |
| rs10639168 | 110893102 | 0.99 | 0.90 | 0.88-0.92 | 1.90 x 10^-24^ |
| rs5899787 | 110893551 | 0.99 | 0.90 | 0.88-0.92 | 1.45 x 10^-24^ |
| rs34138847 | 110893720 | 0.99 | 0.90 | 0.88-0.92 | 2.00 x 10^-24^ |
| rs659713 | 110893949 | 1 | 0.90 | 0.88-0.92 | 7.82 x 10^-25^ |
| rs676256 | 110895353 | 1 | 0.90 | 0.88-0.92 | 1.10 x 10^-25^ |
| rs3119744 | 110895863 | 1 | 0.90 | 0.88-0.92 | 3.28 x 10^-25^ |

**Supplemental Table 4A. Association of rs676256 and rs10816625 with risk of breast cancer stratified by lymph node status.**

| **Locus** | **Population** | **Controls** | **Cases** | **OR^1^** | **95% CI** | ***P*-value^2^** | **OR^1^** | **95% CI** | ***P*-value^2^** | ***P*_het_^3^** |
| --- | --- | --- | --- | --- | --- | --- | --- | --- | --- | --- |
|  | Caucasian |  |  | **Node negative tumours** | | | **Node positive tumours** | | |  |
| rs10816625 |  | 40,551 | 18,245 | 11,371 | 1.15 | 1.09-1.21 | 5.20 x 10^-08^ | 1.07 | 1.01-1.14 | 0.02 | 0.03 |
| rs13294895 |  | 40,551 | 18,247 | 11,371 | 1.08 | 1.05-1.12 | 1.58 x 10^-06^ | 1.12 | 1.08-1.17 | 5.06 x 10^-09^ | 0.14 |
| rs676256 |  | 40,551 | 18,244 | 11,368 | 0.89 | 0.86-0.91 | 1.72 x 10^-19^ | 0.91 | 0.88-0.94 | 3.91 x 10^-09^ | 0.12 |
|  | Asian |  |  | **Node negative tumours** | | | **Node positive tumours** | | |  |
| rs10816625 |  | 4,741 | 1,815 | 1,257 | 1.11 | 1.02-1.21 | 0.01 | 1.11 | 1.01-1.23 | 0.03 | 0.91 |
| rs13294895 |  | 4,742 | 1,813 | 1,257 | 1.04 | 0.82-1.33 | 0.74 | 1.00 | 0.77-1.31 | 0.99 | 0.75 |
| rs676256 |  | 4,742 | 1,815 | 1,257 | 0.98 | 0.80-1.18 | 0.80 | 1.09 | 0.89-1.34 | 0.39 | 0.38 |

1. Stratum specific ORs estimated using polytomous logistic regression.

2. Stratum specific *P*-values computed using Wald tests.

3. *P*-value for heterogeneity in effect estimates between strata calculated using case-only logistic regression.

**Supplemental Table 4B. Association of rs676256 and rs10816625 with risk of ER-negative breast cancer stratified by lymph node status.**

| **Locus** | **Population** | **Controls** | **Cases** | **OR^1^** | **95% CI** | ***P*-value^2^** | **OR^1^** | **95% CI** | ***P*-value^2^** | ***P*_het_^3^** |
| --- | --- | --- | --- | --- | --- | --- | --- | --- | --- | --- |
|  | Caucasian |  |  | **Node negative tumours** | | | **Node positive tumours** | | |  |
| rs10816625 |  | 39,522 | 3,088 | 2,190 | 1.05 | 0.94-1.17 | 0.42 | 1.11 | 0.98-1.26 | 0.10 | 0.47 |
| rs13294895 |  | 39,522 | 3,090 | 2,190 | 1.01 | 0.94-1.08 | 0.78 | 1.05 | 0.96-1.13 | 0.28 | 0.53 |
| rs676256 |  | 39,527 | 3,090 | 2,188 | 0.99 | 0.93-1.04 | 0.59 | 0.96 | 0.90-1.03 | 0.24 | 0.54 |
|  | Asian |  |  | **Node negative tumours** | | | **Node positive tumours** | | |  |
| rs10816625 |  | 4,741 | 530 | 390 | 1.08 | 0.95-1.24 | 0.24 | 1.17 | 1.00-1.37 | 0.05 | 0.43 |
| rs13294895 |  | 4,742 | 529 | 390 | 0.78 | 0.50-1.21 | 0.27 | 0.90 | 0.58-1.42 | 0.66 | 0.72 |
| rs676256 |  | 4,742 | 530 | 390 | 0.97 | 0.71-1.33 | 0.84 | 1.23 | 0.90-1.69 | 0.20 | 0.31 |

1. Stratum specific ORs estimated using polytomous logistic regression.

2. Stratum specific *P*-values computed using Wald tests.

3. *P*-value for heterogeneity in effect estimates between strata calculated using case-only logistic regression.

**Supplemental Table 5. Association of rs676256 and rs10816625 with breast cancer stratified by morphology.**

| **Locus** | **Population** | **Controls** | **Cases** | **OR^1^** | **95% CI** | ***P*-value^2^** | **OR^1^** | **95% CI** | ***P*-value^2^** | ***P*_het_^3^** |
| --- | --- | --- | --- | --- | --- | --- | --- | --- | --- | --- |
|  | Caucasian |  |  | **Ductal tumours** | | | **Lobular tumours** | | |  |
| rs10816625 |  | 34,319 | 23,532 | 4,146 | 1.09 | 1.04-1.14 | 6.50 x 10^-04^ | 1.18 | 1.07-1.29 | 4.15 x 10^-04^ | 0.1 |
| rs13294895 |  | 34,317 | 23,533 | 4,146 | 1.08 | 1.04-1.11 | 3.81 x 10^-06^ | 1.12 | 1.06-1.19 | 1.03 x 10^-04^ | 0.12 |
| rs676256 |  | 34,318 | 23,528 | 4,146 | 0.91 | 0.89-0.93 | 2.73 x 10^-14^ | 0.85 | 0.81-0.89 | 2.20 x 10^-11^ | 0.006 |
|  | Asian |  |  | **Ductal tumours** | | | **Lobular tumours** | | |  |
| rs10816625 |  | 4,744 | 3,181 | 112 | 1.13 | 1.06-1.22 | 5.57 x 10^-04^ | 1.35 | 1.03-1.78 | 0.03 | 0.10 |
| rs13294895 |  | 4,745 | 3,179 | 112 | 1.08 | 0.88-1.31 | 0.47 | 1.07 | 0.47-2.45 | 0.87 | 0.98 |
| rs676256 |  | 4,745 | 3,181 | 112 | 0.94 | 0.80-1.12 | 0.49 | 1.59 | 0.92-2.73 | 0.09 | 0.18 |

1. Stratum specific ORs estimated using polytomous logistic regression.

2. Stratum specific *P*-values computed using Wald tests.

3. *P*-value for heterogeneity in effect estimates between strata calculated using case-only logistic regression.

**Supplemental Table 6A. Association of rs676256 and rs10816625 with breast cancer stratified by tumour grade.**

| **Locus** | **Population** | **Controls** | **Cases^1^** | **Grade** | **OR^2^** | **95% CI** | ***P*-value^3^** | ***P*_trend_^4^** |
| --- | --- | --- | --- | --- | --- | --- | --- | --- |
| rs10816625 | Caucasian | 39,762 | 6,176 | 1 | 1.13 | 1.05-1.22 | 0.002 |  |
|  |  |  | 14,060 | 2 | 1.12 | 1.06-1.19 | 4.00 x 10^-05^ |  |
|  |  |  | 9,094 | 3 | 1.04 | 0.97-1.11 | 0.26 | 0.05 |
| rs13294895 |  | 39,763 | 6,176 | 1 | 1.07 | 1.02-1.13 | 0.004 |  |
|  |  |  | 14,060 | 2 | 1.10 | 1.07-1.14 | 4.52 x 10^-08^ |  |
|  |  |  | 9,095 | 3 | 1.08 | 1.03-1.12 | 8.21 x 10^-04^ | 0.75 |
| rs676256 |  | 39,763 | 6,175 | 1 | 0.88 | 0.84-0.92 | 3.2 x 10^-10^ |  |
|  |  |  | 14,055 | 2 | 0.87 | 0.84-0.89 | 2.50 x 10^-22^ |  |
|  |  |  | 9,095 | 3 | 0.94 | 0.91-0.97 | 2.08 x 10^-04^ | 0.004 |
| rs10816625 | Asian | 4,488 | 420 | 1 | 1.04 | 0.90-1.22 | 0.58 |  |
|  |  |  | 1,353 | 2 | 1.10 | 1.00-1.21 | 0.05 |  |
|  |  |  | 1,032 | 3 | 1.22 | 1.10-1.36 | 1.82 x 10^-04^ | 0.02 |
| rs13294895 |  | 4,489 | 420 | 1 | 0.89 | 0.56-1.42 | 0.62 |  |
|  |  |  | 1,352 | 2 | 1.14 | 0.87-1.49 | 0.34 |  |
|  |  |  | 1,032 | 3 | 1.03 | 0.76-1.39 | 0.85 | 0.89 |
| rs676256 |  | 4,489 | 420 | 1 | 0.98 | 0.71-1.37 | 0.93 |  |
|  |  |  | 1,353 | 2 | 1.04 | 0.84-1.29 | 0.71 |  |
|  |  |  | 1,032 | 3 | 0.81 | 0.63-1.06 | 0.12 | 0.12 |
|  |  |  |  |  |  |  |  |  |

1. Maximum total number of cases for each stratum.

2. Stratum specific ORs estimated using polytomous logistic regression.

3. Stratum specific *P*-values computed using Wald tests.

4. *P*-value for linear trend in effect estimates across strata calculated using case-only logistic regression.

**Supplemental Table 6B.**

**Association of rs676256 and rs10816625 with ER-negative breast cancer stratified by tumour grade.**

| **Locus** | **Population** | **Controls** | **Cases^1^** | **Grade** | **OR^2^** | **95% CI** | ***P*-value^3^** | ***P*_trend_^4^** |
| --- | --- | --- | --- | --- | --- | --- | --- | --- |
| rs10816625 | Caucasian | 39,762 | 6,176 | 1 | 1.13 | 1.05-1.22 | 0.002 |  |
|  |  |  | 14,060 | 2 | 1.12 | 1.06-1.19 | 4.00 x 10^-05^ |  |
|  |  |  | 9,094 | 3 | 1.04 | 0.97-1.11 | 0.26 | 0.05 |
| rs13294895 |  | 39,763 | 6,176 | 1 | 1.07 | 1.02-1.13 | 0.004 |  |
|  |  |  | 14,060 | 2 | 1.10 | 1.07-1.14 | 4.52 x 10^-08^ |  |
|  |  |  | 9,095 | 3 | 1.08 | 1.03-1.12 | 8.21 x 10^-04^ | 0.75 |
| rs676256 |  | 39,763 | 6,175 | 1 | 0.88 | 0.84-0.92 | 3.2 x 10^-10^ |  |
|  |  |  | 14,055 | 2 | 0.87 | 0.84-0.89 | 2.50 x 10^-22^ |  |
|  |  |  | 9,095 | 3 | 0.94 | 0.91-0.97 | 2.08 x 10^-04^ | 0.004 |
|  |  |  |  |  |  |  |  |  |

1. Maximum total number of cases for each stratum.

2. Stratum specific ORs estimated using polytomous logistic regression.

3. Stratum specific *P*-values computed using Wald tests.

4. *P*-value for linear trend in effect estimates across strata calculated using case-only logistic regression.

**Supplemental Table 7. Association of rs13294895, rs10816625 and rs676256 with all breast cancers and ER-negative breast cancers in Europeans, stratified by age at diagnosis.**

| **Locus** | **Controls** | **Cases^1^** | **Age Group** | **OR^2^** | **95% CI** | ***P*-value^3^** | ***P*_trend_^4^** |
| --- | --- | --- | --- | --- | --- | --- | --- |
| **rs10816625** |  | **All cases** |  |  |  |  |  |
|  | 32,709 | 2,596 | <40 | 1.14 | 1.02-1.29 | 0.02 |  |
|  |  | 7,421 | 40-49 | 1.20 | 1.12-1.29 | 8.69 x 10^-07^ |  |
|  |  | 11,356 | 50-59 | 1.08 | 1.02-1.15 | 0.01 |  |
|  |  | 9,498 | 60-69 | 1.10 | 1.03-1.18 | 0.004 |  |
|  |  | 3,779 | ≥70 | 1.11 | 1.01-1.22 | 0.03 | 0.06 |
|  |  | **ER-negative cases** |  |  |  |  |  |
|  | 27,294 | 510 | <40 | 1.19 | 0.95-1.48 | 0.13 |  |
|  |  | 1,095 | 40-49 | 1.25 | 1.06-1.47 | 0.009 |  |
|  |  | 1,543 | 50-59 | 0.99 | 0.85-1.16 | 0.91 |  |
|  |  | 1,070 | 60-69 | 0.91 | 0.76-1.10 | 0.34 |  |
|  |  | 448 | ≥70 | 1.10 | 0.87-1.38 | 0.42 | 0.14 |
| **rs13294895** |  | **All cases** |  |  |  |  |  |
|  | 32,709 | 2,597 | <40 | 1.06 | 0.98-1.14 | 0.14 |  |
|  |  | 7,421 | 40-49 | 1.13 | 1.08-1.19 | 2.46 x 10^-07^ |  |
|  |  | 11.356 | 50-59 | 1.14 | 1.09-1.18 | 1.49 x 10^-10^ |  |
|  |  | 9,499 | 60-69 | 1.06 | 1.02-1.11 | 0.06 |  |
|  |  | 3,779 | ≥70 | 1.00 | 0.94-1.07 | 0.90 | 0.87 |
|  |  | **ER-negative cases** |  |  |  |  |  |
|  | 27,295 | 511 | <40 | 0.90 | 0.77-1.04 | 0.16 |  |
|  |  | 1,095 | 40-49 | 0.98 | 0.88-1.09 | 0.69 |  |
|  |  | 1,543 | 50-59 | 0.98 | 0.89-1.08 | 0.70 |  |
|  |  | 1,070 | 60-69 | 1.10 | 0.98-1.23 | 0.11 |  |
|  |  | 448 | ≥70 | 1.06 | 0.91-1.23 | 0.44 | 0.66 |
| **rs676256** |  | **All cases** |  |  |  |  |  |
|  | 32,710 | 2,596 | <40 | 0.92 | 0.86-0.97 | 0.005 |  |
|  |  | 7,421 | 40-49 | 0.87 | 0.83-0.90 | 2.17 x 10^-13^ |  |
|  |  | 11,355 | 50-59 | 0.87 | 0.84-0.93 | 1.74 x 10^-17^ |  |
|  |  | 9,497 | 60-69 | 0.93 | 0.90-0.96 | 3.35 x 10^-05^ |  |
|  |  | 3,776 | ≥70 | 0.94 | 0.90-0.99 | 0.02 | 0.34 |
|  |  | **ER-negative cases** |  |  |  |  |  |
|  | 27,295 | 511 | <40 | 1.00 | 0.89-1.12 | 0.99 |  |
|  |  | 1,095 | 40-49 | 1.05 | 1.02-1.07 | 0.75 |  |
|  |  | 1,543 | 50-59 | 1.03 | 0.96-1.11 | 0.40 |  |
|  |  | 1,070 | 60-69 | 0.98 | 0.89-1.07 | 0.65 |  |
|  |  | 446 | ≥70 | 0.95 | 0.85-1.07 | 0.43 | 0.54 |

1. Maximum total number of cases for each stratum.

2. Stratum specific ORs estimated using polytomous logistic regression.

3. Stratum specific *P*-values computed using Wald tests.

4. P-value for linear trend in effect estimates across strata calculated using case-only logistic regression.

**Supplemental Table 8. Association of rs10816625, rs13294895 and rs676256 with breast cancer risk according to either family history of breast cancer or bilaterality.**

| **Locus** | **Case Group** | **MAF^1^** | **Subjects^2^** | ***P*-value** | **OR** | **95%CI** |
| --- | --- | --- | --- | --- | --- | --- |
| rs10816625 | Sporadic | 0.063 | 0.068 | 32,176 | 20,396 | 9.70 x 10^-04^ | 1.09 | 1.04-1.15 |
|  | Familial | 0.062 | 0.070 | 35,514 | 7,605 | 3.42 x 10^-03^ | 1.12 | 1.04-1.20 |
|  | Bilateral | 0.061 | 0.068 | 28,517 | 1,769 | 1.04 x 10^-01^ | 1.12 | 0.98-1.29 |
| rs13294895 | Sporadic | 0.173 | 0.183 | 32,174 | 20,397 | 6.66 x 10^-04^ | 1.06 | 1.02-1.10 |
|  | Familial | 0.174 | 0.185 | 35,512 | 7,605 | 4.40 x 10^-05^ | 1.10 | 1.05-1.16 |
|  | Bilateral | 0.174 | 0.190 | 28,516 | 1,769 | 2.56 x 10^-03^ | 1.15 | 1.05-1.25 |
| rs676256 | Sporadic | 0.382 | 0.356 | 32,175 | 20,394 | 7.47 x 10^-13^ | 0.91 | 0.88-0.93 |
|  | Familial | 0.383 | 0.349 | 35,513 | 7,605 | 9.50 x 10^-13^ | 0.87 | 0.84-0.90 |
|  | Bilateral | 0.383 | 0.349 | 28,516 | 1,769 | 8.93 x 10^-04^ | 0.88 | 0.82-0.95 |

1. Minor allele frequency in controls and in cases.

2. Number of controls and cases in each group.
